# Supplementary material for: Predicting soft tissue thicknesses overlying the iliac crests and greater trochanters of younger and older adults
Source: PLoS One. 2023 Mar 14;18(3):e0283012. doi: 10.1371/journal.pone.0283012 (PMC10013917; doi:10.1371/journal.pone.0283012)
Supplement: S1 File — (PDF) [file pone.0283012.s001.pdf]

Regression

Notes

Output Created		22-NOV-2017 13:53...
Comments		
Input	Data	F:\Claudia Regression\Danielle FIX\Generation-Validation Groups\SPSS Data_REGRESSION_G groups final_YOUNG_no 0fm488+0mt203.sav
	Active Dataset	DataSet2
	Filter	<none>
	Weight	<none>
	Split File	<none>
	N of Rows in Working Data File	74
Missing Value Handling	Definition of Missing	User-defined missing values are treated as missing.
	Cases Used	Statistics are based on cases with no missing values for any variable used.

Notes

Syntax		REGRESSION<br>/MISSING LISTWISE<br>/STATISTICS COEFF OUTS BCOV R ANOVA COLLIN TOL<br>/CRITERIA=PIN(.05) POUT(.10)<br>/NOORIGIN<br>/DEPENDENT ICSofttissueRcm<br>/METHOD=STEPWISE Age SexCode0F1M Heightm BodyMasskg waist_circumference waist_breadth_ML<br>waist_breadth_AP suprailiac_SF abdomen_SF ant_pelvis_length lat_pelvis_length_right AVG_lat_pelvis<br>pelvis_circumference hip_circumference hip_breadth_ML rightmed.thigh_length rightmid.thigh_length<br><br>upper_thigh_circumference_R midthigh_circumference_R upperthigh_AP_breadth_R maxthigh_ML_breadth_R<br><br>maxthigh_AP_breadth_R ant_midthigh_SF_R post_midthigh_SF_R<br>/SCATTERPLOT=(*ZRESID ,*ZPRED)<br>/RESIDUALS HISTOGRAM(ZRESID) NORMPROB(ZRESID)<br>/CASEWISE PLOT(ZRESID) OUTLIERS(3)<br>/SAVE ZPRED MAHAL COOK ZRESID.
Resources	Processor Time	00:00:00.47
	Elapsed Time	00:00:00.35
	Memory Required	18468 bytes
	Additional Memory Required for Residual Plots	728 bytes

Notes

Variables Created or Modified	ZPR_8	Standardized Predicted Value
	ZRE_8	Standardized Residual
	MAH_8	Mahalanobis Distance
	COO_8	Cook's Distance

Variables Entered/Removed[a]

Model	Variables Entered	Variables Removed	Method
1	abdomen_SF	.	Stepwise (Criteria: Probability-of-F-to-enter <= .050, Probability-of-F-to-remove >= .100).
2	ant_mid-thigh_SF_R	.	Stepwise (Criteria: Probability-of-F-to-enter <= .050, Probability-of-F-to-remove >= .100).
3	upper_thigh_circumference_R	.	Stepwise (Criteria: Probability-of-F-to-enter <= .050, Probability-of-F-to-remove >= .100).
4	Height (m)	.	Stepwise (Criteria: Probability-of-F-to-enter <= .050, Probability-of-F-to-remove >= .100).

a. Dependent Variable: ICSoft tissue R (cm)

Model Summary[e]

Model	R	R Square	Adjusted R Square	Std. Error of the Estimate
1	.695[a]	.483	.476	.89612
2	.762[b]	.581	.569	.81277
3	.803[c]	.644	.629	.75401
4	.849[d]	.720	.704	.67322

a. Predictors: (Constant), abdomen_SF

b. Predictors: (Constant), abdomen_SF, ant_mid-thigh_SF_R

c. Predictors: (Constant), abdomen_SF, ant_mid-thigh_SF_R, upper_thigh_circumference_R

d. Predictors: (Constant), abdomen_SF, ant_mid-thigh_SF_R, upper_thigh_circumference_R, Height (m)

e. Dependent Variable: ICSoft tissue R (cm)

ANOVA[a]

Model		Sum of Squares	df	Mean Square	F	Sig.
1	Regression	54.057	1	54.057	67.316	.000[b]
	Residual	57.818	72	.803		
	Total	111.876	73			
2	Regression	64.974	2	32.487	49.178	.000[c]
	Residual	46.902	71	.661		
	Total	111.876	73			
3	Regression	72.078	3	24.026	42.260	.000[d]
	Residual	39.797	70	.569		
	Total	111.876	73			
4	Regression	80.603	4	20.151	44.461	.000[e]
	Residual	31.273	69	.453		
	Total	111.876	73			

a. Dependent Variable: ICSoft tissue R (cm)

b. Predictors: (Constant), abdomen_SF

c. Predictors: (Constant), abdomen_SF, ant_mid-thigh_SF_R

d. Predictors: (Constant), abdomen_SF, ant_mid-thigh_SF_R, upper_thigh_circumference_R

e. Predictors: (Constant), abdomen_SF, ant_mid-thigh_SF_R, upper_thigh_circumference_R, Height (m)

Coefficients[a]

Model		Unstandardized Coefficients B	Std. Error	Standardized Coefficients Beta	t
1	(Constant)	.823	.228		3.607
	abdomen_SF	.087	.011	.695	8.205
2	(Constant)	.608	.214		2.848
	abdomen_SF	.056	.012	.447	4.560
	ant_mid-thigh_SF_R	.042	.010	.399	4.065
3	(Constant)	-3.821	1.268		-3.012
	abdomen_SF	.043	.012	.345	3.606
	ant_mid-thigh_SF_R	.039	.010	.362	3.948
	upper_thigh_circumference_R	.078	.022	.283	3.535
4	(Constant)	1.871	1.734		1.079
	abdomen_SF	.046	.011	.370	4.325
	ant_mid-thigh_SF_R	.021	.010	.198	2.197
	upper_thigh_circumference_R	.106	.021	.381	5.081
	Height (m)	-4.116	.949	-.314	-4.337

Coefficients[a]

Model		Sig.	Collinearity Statistics Tolerance	VIF
1	(Constant)	.001		
	abdomen_SF	.000	1.000	1.000
2	(Constant)	.006		
	abdomen_SF	.000	.614	1.630
	ant_mid-thigh_SF_R	.000	.614	1.630
3	(Constant)	.004		
	abdomen_SF	.001	.557	1.796
	ant_mid-thigh_SF_R	.000	.606	1.651
	upper_thigh_circumference_R	.001	.792	1.263
4	(Constant)	.284		
	abdomen_SF	.000	.554	1.804
	ant_mid-thigh_SF_R	.031	.499	2.003
	upper_thigh_circumference_R	.000	.720	1.388
	Height (m)	.000	.771	1.297

a. Dependent Variable: ICSoft tissue R (cm)

Excluded Variables[a]

Model		Beta In	t	Sig.	Partial Correlation	Collinearity. Tolerance
1	Age	.008[b]	.090	.928	.011	1.000
	Sex Code (0=F,1=M)	-.293[b]	-3.748	.000	-.406	.996
	Height (m)	-.293[b]	-3.739	.000	-.406	.989
	Body Mass (kg)	-.072[b]	-.822	.414	-.097	.935
	waist_circumference	-.182[b]	-1.915	.060	-.222	.766
	waist_breadth_M-L	-.242[b]	-2.790	.007	-.314	.872
	waist_breadth_A-P	-.092[b]	-.906	.368	-.107	.704
	suprailiac_SF	.215[b]	1.343	.184	.157	.276
	ant_pelvis_length	.075[b]	.879	.383	.104	.992
	lat_pelvis_length_right	.008[b]	.097	.923	.012	.990
	AVG_lat_pelvis	.002[b]	.029	.977	.003	.983
	pelvis_circumference	-.031[b]	-.272	.787	-.032	.559
	hip_circumference	.194[b]	2.063	.043	.238	.779
	hip_breadth_M-L	.155[b]	1.691	.095	.197	.830
	right med. thigh_length	-.202[b]	-2.455	.017	-.280	.991
	right mid. thigh_length	.156[b]	1.875	.065	.217	.998
	upper_thigh_circumference_R	.319[b]	3.656	.000	.398	.803
	mid-thigh_circumference_R	.163[b]	1.766	.082	.205	.816
	upperthigh_A-P_breadth_R	.302[b]	3.367	.001	.371	.781
	max-thigh_M-L_breadth_R	.070[b]	.816	.417	.096	.970
	max-thigh_A-P_breadth_R	.103[b]	1.180	.242	.139	.934
	ant_mid-thigh_SF_R	.399[b]	4.065	.000	.435	.614
	post_mid-thigh_SF_R	.287[b]	3.152	.002	.350	.768
2	Age	-.007[c]	-.095	.924	-.011	.997
	Sex Code (0=F,1=M)	-.154[c]	-1.534	.130	-.180	.572
	Height (m)	-.204[c]	-2.529	.014	-.289	.848
	Body Mass (kg)	.044[c]	.518	.606	.062	.825
	waist_circumference	-.017[c]	-.167	.868	-.020	.594

Excluded Variables[a]

Model		Collinearity Statistics VIF	Minimum Tolerance
1	Age	1.000	1.000
	Sex Code (0=F,1=M)	1.004	.996
	Height (m)	1.011	.989
	Body Mass (kg)	1.069	.935
	waist_circumference	1.306	.766
	waist_breadth_M-L	1.147	.872
	waist_breadth_A-P	1.421	.704
	suprailiac_SF	3.628	.276
	ant_pelvis_length	1.008	.992
	lat_pelvis_length_right	1.010	.990
	AVG_lat_pelvis	1.017	.983
	pelvis_circumference	1.790	.559
	hip_circumference	1.283	.779
	hip_breadth_M-L	1.204	.830
	right med. thigh_length	1.009	.991
	right mid. thigh_length	1.002	.998
	upper_thigh_circumference_R	1.246	.803
	mid-thigh_circumference_R	1.225	.816
	upperthigh_A-P_breadth_R	1.281	.781
	max-thigh_M-L_breadth_R	1.030	.970
	max-thigh_A-P_breadth_R	1.071	.934
	ant_mid-thigh_SF_R	1.630	.614
	post_mid-thigh_SF_R	1.303	.768
2	Age	1.003	.612
	Sex Code (0=F,1=M)	1.747	.353
	Height (m)	1.180	.526
	Body Mass (kg)	1.212	.512
	waist_circumference	1.683	.365

Excluded Variables[a]

Model		Beta In	t	Sig.	Partial Correlation	Collinearity. Tolerance
	waist_breadth_M-L	-.091[c]	-.936	.353	-.111	.629
	waist_breadth_A-P	.025[c]	.257	.798	.031	.638
	suprailiac_SF	.296[c]	2.049	.044	.238	.271
	ant_pelvis_length	.093[c]	1.202	.233	.142	.989
	lat_pelvis_length_right	.023[c]	.296	.768	.035	.988
	AVG_lat_pelvis	.020[c]	.257	.798	.031	.980
	pelvis_circumference	.031[c]	.294	.770	.035	.547
	hip_circumference	.166[c]	1.941	.056	.226	.774
	hip_breadth_M-L	.064[c]	.723	.472	.086	.765
	right med. thigh_length	-.106[c]	-1.299	.198	-.153	.873
	right mid. thigh_length	.077[c]	.957	.342	.114	.923
	upper_thigh_circumference_R	.283[c]	3.535	.001	.389	.792
	mid-thigh_circumference_R	.178[c]	2.150	.035	.249	.815
	upperthigh_A-P_breadth_R	.275[c]	3.375	.001	.374	.776
	max-thigh_M-L_breadth_R	.035[c]	.446	.657	.053	.958
	max-thigh_A-P_breadth_R	.180[c]	2.275	.026	.262	.892
	post_mid-thigh_SF_R	-.012[c]	-.081	.936	-.010	.254
3	Age	.007[d]	.097	.923	.012	.994
	Sex Code (0=F,1=M)	-.274[d]	-2.927	.005	-.332	.525
	Height (m)	-.314[d]	-4.337	.000	-.463	.771
	Body Mass (kg)	-.390[d]	-3.495	.001	-.388	.351
	waist_circumference	-.307[d]	-2.846	.006	-.324	.397
	waist_breadth_M-L	-.305[d]	-3.193	.002	-.359	.492
	waist_breadth_A-P	-.230[d]	-2.175	.033	-.253	.430
	suprailiac_SF	.172[d]	1.211	.230	.144	.249
	ant_pelvis_length	.058[d]	.792	.431	.095	.968
	lat_pelvis_length_right	-.012[d]	-.169	.866	-.020	.969
	AVG_lat_pelvis	-.021[d]	-.292	.771	-.035	.955
	pelvis_circumference	-.308[d]	-2.574	.012	-.296	.328
	hip_circumference	-.105[d]	-.864	.391	-.103	.345

Excluded Variables[a]

Model		Collinearity Statistics VIF	Collinearity Statistics Minimum Tolerance
	waist_breadth_M-L	1.590	.397
	waist_breadth_A-P	1.568	.399
	suprailiac_SF	3.687	.215
	ant_pelvis_length	1.011	.612
	lat_pelvis_length_right	1.012	.607
	AVG_lat_pelvis	1.020	.602
	pelvis_circumference	1.829	.376
	hip_circumference	1.291	.540
	hip_breadth_M-L	1.308	.565
	right med. thigh_length	1.145	.541
	right mid. thigh_length	1.083	.568
	upper_thigh_circumference_R	1.263	.557
	mid-thigh_circumference_R	1.227	.529
	upperthigh_A-P_breadth_R	1.289	.541
	max-thigh_M-L_breadth_R	1.044	.606
	max-thigh_A-P_breadth_R	1.122	.547
	post_mid-thigh_SF_R	3.934	.203
3	Age	1.006	.556
	Sex Code (0=F,1=M)	1.907	.326
	Height (m)	1.297	.499
	Body Mass (kg)	2.848	.337
	waist_circumference	2.518	.357
	waist_breadth_M-L	2.031	.385
	waist_breadth_A-P	2.327	.395
	suprailiac_SF	4.010	.215
	ant_pelvis_length	1.033	.553
	lat_pelvis_length_right	1.032	.555
	AVG_lat_pelvis	1.047	.553
	pelvis_circumference	3.049	.328
	hip_circumference	2.895	.345

Excluded Variables[a]

Model		Beta In	t	Sig.	Partial Correlation	Collinearity. Tolerance
	hip_breadth_M-L	-.145[d]	-1.481	.143	-.176	.521
	right med. thigh_length	-.178[d]	-2.338	.022	-.271	.827
	right mid. thigh_length	.040[d]	.527	.600	.063	.904
	mid-thigh_circumference_R	-.216[d]	-1.445	.153	-.171	.224
	upperthigh_A-P_breadth_R	.131[d]	1.001	.320	.120	.296
	max-thigh_M-L_breadth_R	-.228[d]	-2.466	.016	-.285	.554
	max-thigh_A-P_breadth_R	-.040[d]	-.353	.725	-.043	.401
	post_mid-thigh_SF_R	.053[d]	.370	.713	.045	.250
4	Age	-.014[e]	-.216	.829	-.026	.988
	Sex Code (0=F,1=M)	-.068[e]	-.615	.541	-.074	.333
	Body Mass (kg)	.039[e]	.184	.854	.022	.092
	waist_circumference	-.055[e]	-.425	.672	-.051	.243
	waist_breadth_M-L	-.093[e]	-.790	.432	-.095	.295
	waist_breadth_A-P	-.053[e]	-.484	.630	-.059	.344
	suprailiac_SF	.140[e]	1.097	.277	.132	.248
	ant_pelvis_length	.079[e]	1.219	.227	.146	.963
	lat_pelvis_length_right	.049[e]	.743	.460	.090	.926
	AVG_lat_pelvis	.041[e]	.619	.538	.075	.910
	pelvis_circumference	-.078[e]	-.593	.555	-.072	.239
	hip_circumference	.204[e]	1.614	.111	.192	.247
	hip_breadth_M-L	.066[e]	.645	.521	.078	.387
	right med. thigh_length	.054[e]	.564	.574	.068	.443
	right mid. thigh_length	.082[e]	1.213	.229	.146	.886
	mid-thigh_circumference_R	-.105[e]	-.763	.448	-.092	.215
	upperthigh_A-P_breadth_R	.080[e]	.680	.499	.082	.293
	max-thigh_M-L_breadth_R	-.164[e]	-1.926	.058	-.227	.535
	max-thigh_A-P_breadth_R	-.030[e]	-.300	.765	-.036	.401
	post_mid-thigh_SF_R	-.109[e]	-.819	.415	-.099	.231

Excluded Variables[a]

Model		Collinearity Statistics VIF	Collinearity Statistics Minimum Tolerance
	hip_breadth_M-L	1.919	.521
	right med. thigh_length	1.209	.521
	right mid. thigh_length	1.106	.540
	mid-thigh_circumference_R	4.473	.217
	upperthigh_A-P_breadth_R	3.375	.296
	max-thigh_M-L_breadth_R	1.804	.458
	max-thigh_A-P_breadth_R	2.492	.356
	post_mid-thigh_SF_R	4.000	.197
4	Age	1.012	.499
	Sex Code (0=F,1=M)	3.005	.323
	Body Mass (kg)	10.925	.092
	waist_circumference	4.118	.243
	waist_breadth_M-L	3.395	.295
	waist_breadth_A-P	2.907	.344
	suprailiac_SF	4.024	.213
	ant_pelvis_length	1.038	.499
	lat_pelvis_length_right	1.080	.499
	AVG_lat_pelvis	1.099	.499
	pelvis_circumference	4.193	.239
	hip_circumference	4.052	.247
	hip_breadth_M-L	2.586	.387
	right med. thigh_length	2.256	.413
	right mid. thigh_length	1.128	.457
	mid-thigh_circumference_R	4.655	.215
	upperthigh_A-P_breadth_R	3.410	.281
	max-thigh_M-L_breadth_R	1.869	.452
	max-thigh_A-P_breadth_R	2.493	.343
	post_mid-thigh_SF_R	4.336	.197

a. Dependent Variable: ICSoft tissue R (cm)

b. Predictors in the Model: (Constant), abdomen_SF

c. Predictors in the Model: (Constant), abdomen_SF, ant_mid-thigh_SF_R

d. Predictors in the Model: (Constant), abdomen_SF, ant_mid-thigh_SF_R, upper_thigh_circumference_R

e. Predictors in the Model: (Constant), abdomen_SF, ant_mid-thigh_SF_R, upper_thigh_circumference_R, Height (m)

Coefficient Correlations[a]

Model			abdomen_SF	ant_mid-thigh_SF_R	upper_thigh_circumference_R
1	Correlations	abdomen_SF	1.000		
	Covariances	abdomen_SF	.000		
2	Correlations	abdomen_SF	1.000	-.622	
		ant_mid-thigh_SF_R	-.622	1.000	
	Covariances	abdomen_SF	.000	-7.927E-5	
		ant_mid-thigh_SF_R	-7.927E-5	.000	
3	Correlations	abdomen_SF	1.000	-.553	-.304
		ant_mid-thigh_SF_R	-.553	1.000	-.115
		upper_thigh_circumference_R	-.304	-.115	1.000
	Covariances	abdomen_SF	.000	-6.418E-5	-8.026E-5
		ant_mid-thigh_SF_R	-6.418E-5	9.515E-5	-2.480E-5
		upper_thigh_circumference_R	-8.026E-5	-2.480E-5	.000
4	Correlations	abdomen_SF	1.000	-.530	-.269
		ant_mid-thigh_SF_R	-.530	1.000	-.225
		upper_thigh_circumference_R	-.269	-.225	1.000
		Height (m)	-.068	.419	-.301
	Covariances	abdomen_SF	.000	-5.408E-5	-5.945E-5
		ant_mid-thigh_SF_R	-5.408E-5	9.202E-5	-4.492E-5
		upper_thigh_circumference_R	-5.945E-5	-4.492E-5	.000
		Height (m)	-.001	.004	-.006

Coefficient Correlations[a]

Model			Height (m)
1	Correlations	abdomen_SF	
	Covariances	abdomen_SF	
2	Correlations	abdomen_SF	
		ant_mid-thigh_SF_R	
	Covariances	abdomen_SF	
		ant_mid-thigh_SF_R	
3	Correlations	abdomen_SF	
		ant_mid-thigh_SF_R	
		upper_thigh_circumference_R	
	Covariances	abdomen_SF	
		ant_mid-thigh_SF_R	
		upper_thigh_circumference_R	
4	Correlations	abdomen_SF	-.068
		ant_mid-thigh_SF_R	.419
		upper_thigh_circumference_R	-.301
		Height (m)	1.000
	Covariances	abdomen_SF	-.001
		ant_mid-thigh_SF_R	.004
		upper_thigh_circumference_R	-.006
		Height (m)	.901

a. Dependent Variable: ICSoft tissue R (cm)

Collinearity Diagnostics[a]

Model	Dimension	Eigenvalue	Condition Index	Variance Proportions (Constant)	abdomen_SF	ant_mid-thigh_SF_R
1	1	1.890	1.000	.06	.06	
	2	.110	4.139	.94	.94	
2	1	2.769	1.000	.02	.02	.02
	2	.147	4.344	.79	.02	.44
	3	.085	5.719	.19	.97	.54
3	1	3.710	1.000	.00	.01	.01
	2	.201	4.293	.01	.07	.34
	3	.086	6.551	.00	.85	.64
	4	.002	40.572	.99	.07	.01
4	1	4.660	1.000	.00	.00	.01
	2	.249	4.323	.00	.08	.24
	3	.087	7.326	.00	.83	.56
	4	.003	41.456	.08	.05	.07
	5	.001	61.678	.92	.03	.13

Collinearity Diagnostics[a]

Model	Dimension	Variance Proportions upper_thigh_circumference_R	Height (m)
1	1		
	2		
2	1		
	2		
	3		
3	1	.00	
	2	.00	
	3	.00	
	4	1.00	
4	1	.00	.00
	2	.00	.00
	3	.00	.00
	4	.99	.17
	5	.01	.83

a. Dependent Variable: ICSoft tissue R (cm)

Residuals Statistics[a]

	Minimum	Maximum	Mean	Std. Deviation	N
Predicted Value	.6388	5.2932	2.4884	1.05079	74
Std. Predicted Value	-1.760	2.669	.000	1.000	74
Standard Error of Predicted Value	.087	.345	.168	.050	74
Adjusted Predicted Value	.6187	5.4333	2.4875	1.05041	74
Residual	-1.56506	1.49018	.00000	.65452	74
Std. Residual	-2.325	2.214	.000	.972	74
Stud. Residual	-2.363	2.446	.001	1.007	74
Deleted Residual	-1.63890	1.81992	.00093	.70323	74
Stud. Deleted Residual	-2.447	2.541	.001	1.022	74
Mahal. Distance	.238	18.199	3.946	3.307	74
Cook's Distance	.000	.265	.015	.033	74
Centered Leverage Value	.003	.249	.054	.045	74

a. Dependent Variable: ICSoft tissue R (cm)

Charts

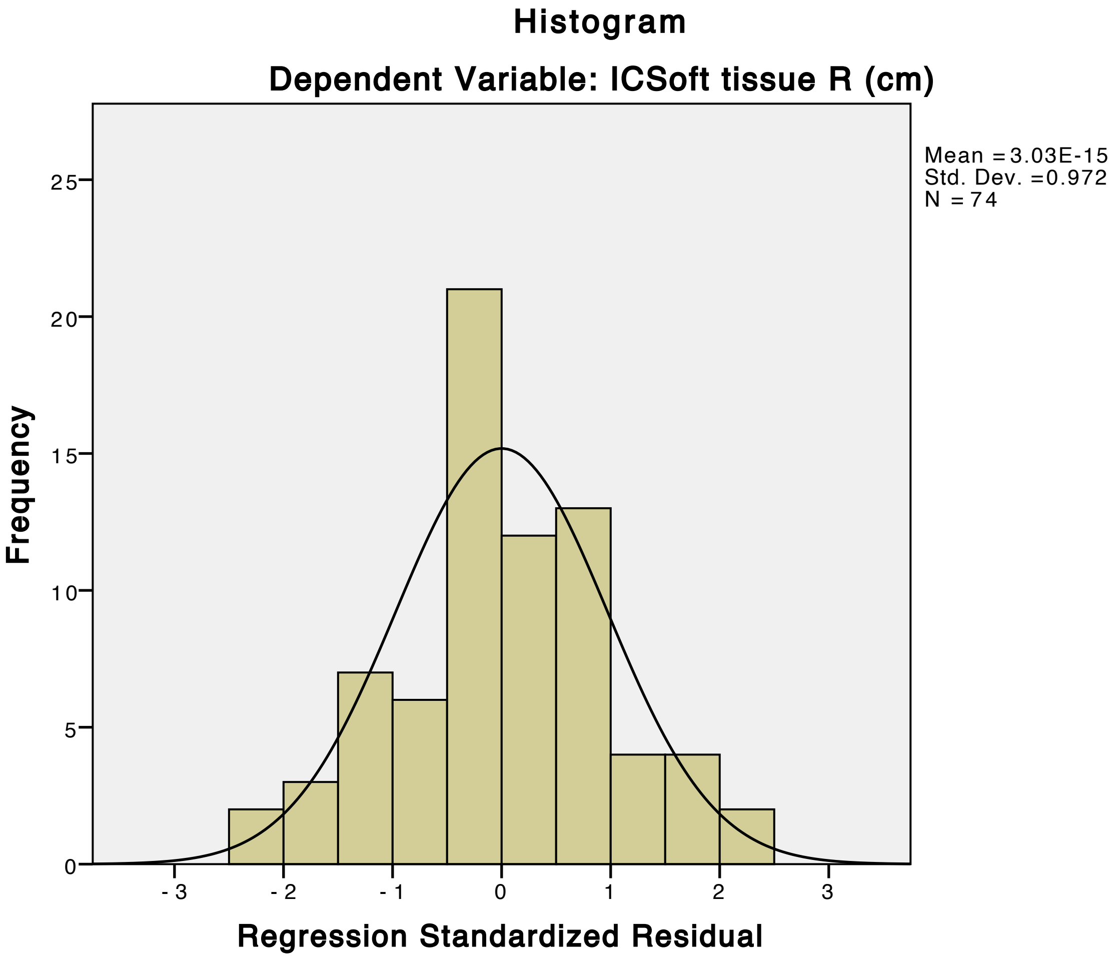
Histogram
Dependent Variable: ICSoft tissue R (cm)
Mean =3.03E-15
Std. Dev. =0.972
N =74
Frequency
0
5
10
15
20
25
-3
-2
-1
0
1
2
3
Regression Standardized Residual

Normal P-P Plot of Regression Standardized Residual

Dependent Variable: ICSoft tissue R (cm)

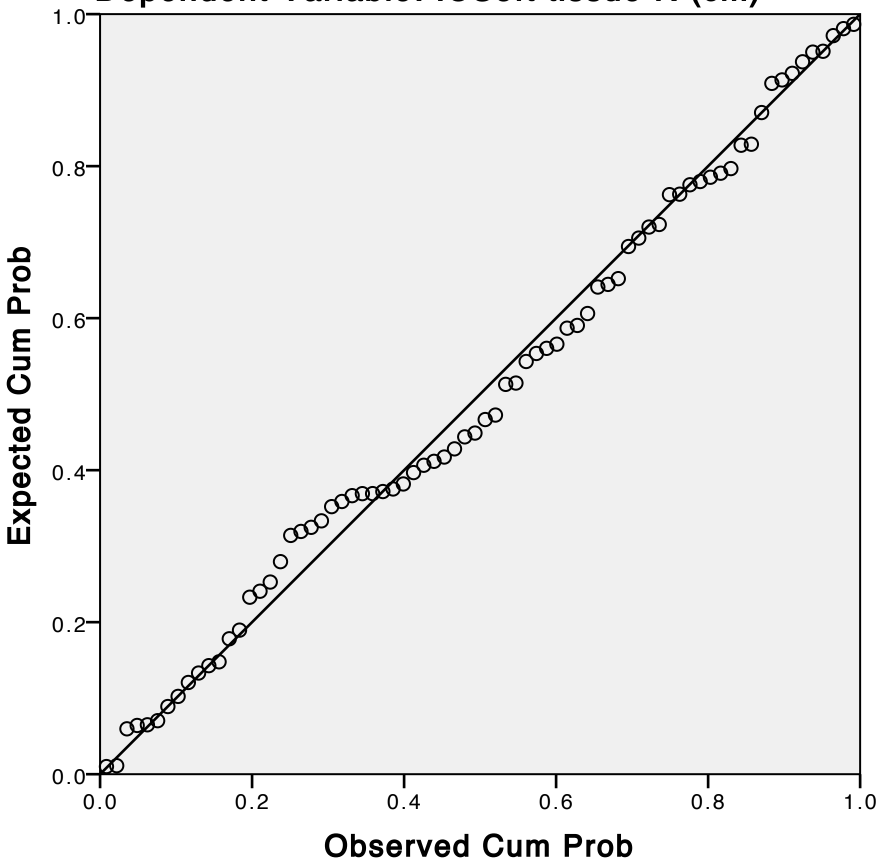

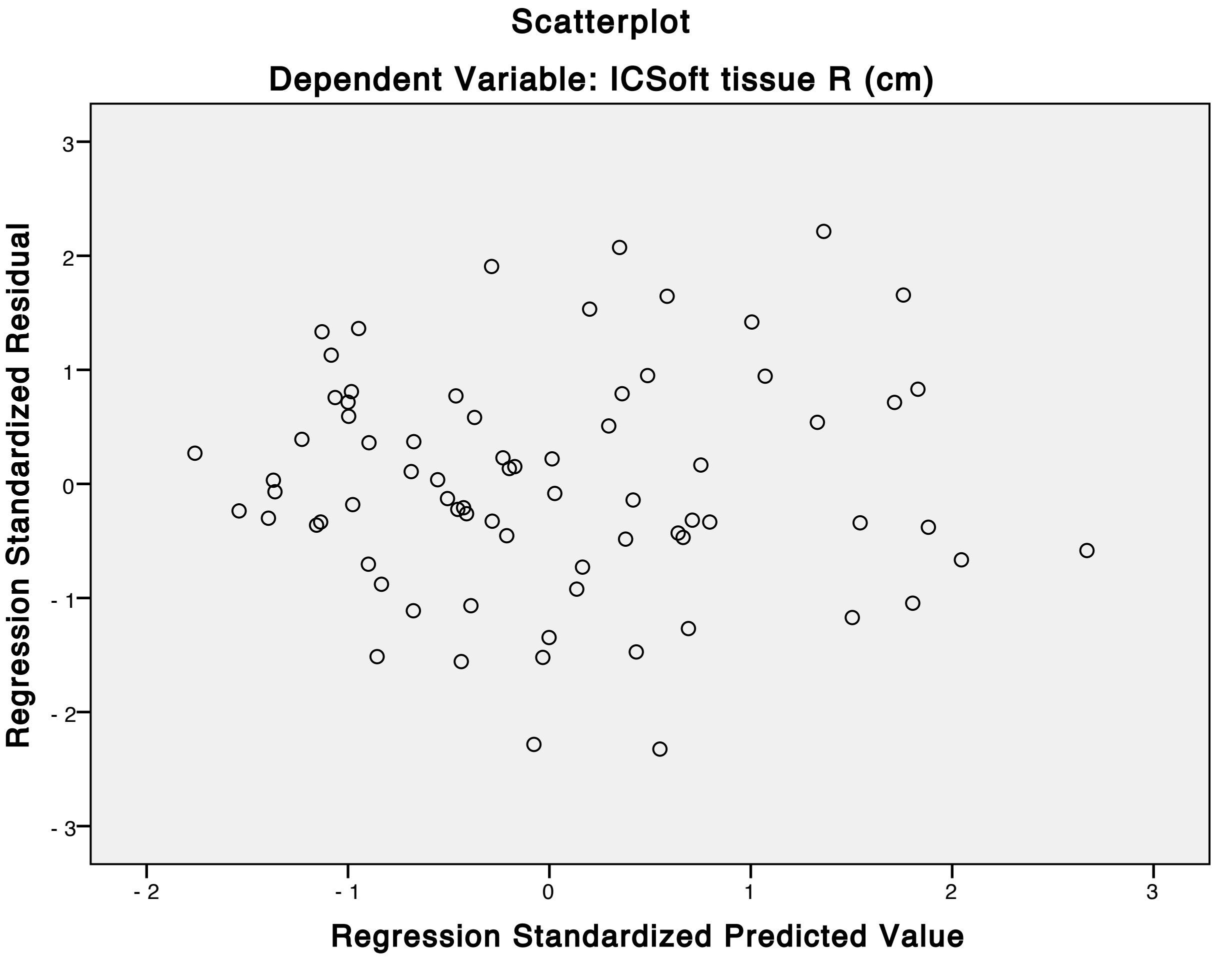
Scatterplot
Dependent Variable: ICSoft tissue R (cm)
Regression Standardized Residual
3
2
1
0
- 1
- 2
- 3
- 2
- 1
0
1
2
3
Regression Standardized Predicted Value

Regression

Notes

Output Created		21-NOV-2017 11:54...
Comments		
Input	Data	F:\Claudia Regression\Danielle FIX\Generation-Validation Groups\SPSS Data_REGRESSION_G groups final_YOUNG_no 0fm488+0mt203.sav
	Active Dataset	DataSet1
	Filter	<none>
	Weight	<none>
	Split File	AgeCode
	N of Rows in Working Data File	74
Missing Value Handling	Definition of Missing	User-defined missing values are treated as missing.
	Cases Used	Statistics are based on cases with no missing values for any variable used.

Notes

Syntax		REGRESSION<br>/MISSING LISTWISE<br>/STATISTICS COEFF OUTS BCOV R ANOVA COLLIN TOL<br>/CRITERIA=PIN(.05) POUT(.10)<br>/NOORIGIN<br>/DEPENDENT IC_SofttissueLcm<br>/METHOD=STEPWISE SexCode0F1M Age Heightm BodyMasskg BMI waist_circumference waist_breadth_ML<br>waist_breadth_AP suprailiac_SF abdomen_SF ant_pelvis_length lat_pelvis_length_left AVG_lat_pelvis<br>pelvis_circumference hip_circumference hip_breadth_ML pelvis_breadth_AP leftlat.thigh_length<br>leftmed.thigh_length leftmid.thigh_length upper_thigh_circumference_L upper_thigh_AP_breadth_L<br><br>maxthigh_ML_breadth_L maxthigh_AP_breadth_L ant_midthigh_SF_L post_midthigh_SF_L<br>/SCATTERPLOT=(*ZRESID ,*ZPRED)<br>/RESIDUALS HISTOGRAM(ZRESID) NORMPROB(ZRESID)<br>/CASEWISE PLOT (ZRESID) OUTLIERS(3)<br>/SAVE ZPRED MAHAL COOK ZRESID.
Resources	Processor Time	00:00:00.53
	Elapsed Time	00:00:00.34
	Memory Required	20220 bytes
	Additional Memory Required for Residual Plots	712 bytes

Notes

Variables Created or Modified	ZPR_3	Standardized Predicted Value
	ZRE_3	Standardized Residual
	MAH_3	Mahalanobis Distance
	COO_3	Cook's Distance

AgeCode = .0

Variables Entered/Removed[a,b]

Model	Variables Entered	Variables Removed	Method
1	ant_mid-thigh_SF_L	.	Stepwise (Criteria: Probability-of-F-to-enter <= .050, Probability-of-F-to-remove >= .100).
2	abdomen_SF	.	Stepwise (Criteria: Probability-of-F-to-enter <= .050, Probability-of-F-to-remove >= .100).
3	Height (m)	.	Stepwise (Criteria: Probability-of-F-to-enter <= .050, Probability-of-F-to-remove >= .100).
4	upper_thigh_circumference_L	.	Stepwise (Criteria: Probability-of-F-to-enter <= .050, Probability-of-F-to-remove >= .100).

a. AgeCode = .0

b. Dependent Variable: IC_Soft tissue L (cm)

Model Summary[a,f]

Model	R	R Square	Adjusted R Square	Std. Error of the Estimate
1	.710[b]	.504	.497	.77505
2	.776[c]	.602	.591	.69908
3	.819[d]	.671	.657	.64027
4	.856[e]	.733	.718	.58052

a. AgeCode = .0

b. Predictors: (Constant), ant_mid-thigh_SF_L

c. Predictors: (Constant), ant_mid-thigh_SF_L, abdomen_SF

d. Predictors: (Constant), ant_mid-thigh_SF_L, abdomen_SF, Height (m)

e. Predictors: (Constant), ant_mid-thigh_SF_L, abdomen_SF, Height (m), upper_thigh_circumference_L

f. Dependent Variable: IC_Soft tissue L (cm)

ANOVA[a,b]

Model		Sum of Squares	df	Mean Square	F	Sig.
1	Regression	43.975	1	43.975	73.206	.000[c]
	Residual	43.251	72	.601		
	Total	87.226	73			
2	Regression	52.527	2	26.263	53.739	.000[d]
	Residual	34.699	71	.489		
	Total	87.226	73			
3	Regression	58.530	3	19.510	47.592	.000[e]
	Residual	28.696	70	.410		
	Total	87.226	73			
4	Regression	63.973	4	15.993	47.458	.000[f]
	Residual	23.253	69	.337		
	Total	87.226	73			

a. AgeCode = .0

b. Dependent Variable: IC_Soft tissue L (cm)

c. Predictors: (Constant), ant_mid-thigh_SF_L

d. Predictors: (Constant), ant_mid-thigh_SF_L, abdomen_SF

e. Predictors: (Constant), ant_mid-thigh_SF_L, abdomen_SF, Height (m)

f. Predictors: (Constant), ant_mid-thigh_SF_L, abdomen_SF, Height (m), upper_thigh_circumference_L

Coefficients[a,b]

Model		Unstandardized Coefficients B	Unstandardized Coefficients Std. Error	Standardized Coefficients Beta	t
1	(Constant)	.819	.178		4.609
	ant_mid-thigh_SF_L	.067	.008	.710	8.556
2	(Constant)	.438	.185		2.373
	ant_mid-thigh_SF_L	.043	.009	.452	4.656
	abdomen_SF	.045	.011	.406	4.183
3	(Constant)	6.260	1.531		4.089
	ant_mid-thigh_SF_L	.029	.009	.307	3.180
	abdomen_SF	.051	.010	.468	5.179
	Height (m)	-3.314	.866	-.287	-3.827
4	(Constant)	3.921	1.505		2.605
	ant_mid-thigh_SF_L	.017	.009	.184	1.987
	abdomen_SF	.044	.009	.402	4.809
	Height (m)	-4.474	.837	-.387	-5.348
	upper_thigh_circumference_L	.078	.019	.303	4.019

Coefficients[a,b]

Model		Sig.	Collinearity Statistics Tolerance	Collinearity Statistics VIF
1	(Constant)	.000		
	ant_mid-thigh_SF_L	.000	1.000	1.000
2	(Constant)	.020		
	ant_mid-thigh_SF_L	.000	.595	1.680
	abdomen_SF	.000	.595	1.680
3	(Constant)	.000		
	ant_mid-thigh_SF_L	.002	.504	1.984
	abdomen_SF	.000	.576	1.736
	Height (m)	.000	.837	1.194
4	(Constant)	.011		
	ant_mid-thigh_SF_L	.051	.449	2.226
	abdomen_SF	.000	.554	1.806
	Height (m)	.000	.738	1.356
	upper_thigh_circumference_L	.000	.680	1.471

a. AgeCode = .0

b. Dependent Variable: IC_Soft tissue L (cm)

Excluded Variables[a,b]

Model		Beta In	t	Sig.	Partial Correlation	Collinearity.
						Tolerance
1	Sex Code (0=F,1=M)	.000[c]	-.001	.999	.000	.690
	Age	-.003[c]	-.033	.974	-.004	.999
	Height (m)	-.217[c]	-2.523	.014	-.287	.865
	Body Mass (kg)	.065[c]	.772	.443	.091	.991
	BMI	.262[c]	3.309	.001	.366	.966
	waist_circumference	.139[c]	1.699	.094	.198	1.000
	waist_breadth_M-L	.064[c]	.756	.452	.089	.978
	waist_breadth_A-P	.156[c]	1.897	.062	.220	.979
	suprailiac_SF	.346[c]	3.926	.000	.422	.740
	abdomen_SF	.406[c]	4.183	.000	.445	.595
	ant_pelvis_length	.054[c]	.649	.518	.077	.991
	lat_pelvis_length_left	.049[c]	.593	.555	.070	.998
	AVG_lat_pelvis	.045[c]	.540	.591	.064	.999
	pelvis_circumference	.185[c]	2.153	.035	.248	.891
	hip_circumference	.171[c]	1.964	.053	.227	.878
	hip_breadth_M-L	.059[c]	.633	.528	.075	.791
	pelvis_breadth_A-P	.240[c]	2.909	.005	.326	.915
	left lat. thigh_length	-.227[c]	-2.612	.011	-.296	.841
	left med. thigh_length	-.148[c]	-1.722	.089	-.200	.908
	left mid. thigh_length	-.031[c]	-.356	.723	-.042	.930
	upper_thigh_circumference_L	.239[c]	2.726	.008	.308	.821
	upper_thigh_A-P_breadth_L	.328[c]	3.957	.000	.425	.835
	max-thigh_M-L_breadth_L	.082[c]	.952	.344	.112	.936
	max-thigh_A-P_breadth_L	.129[c]	1.568	.121	.183	.997
	post_mid-thigh_SF_L	.063[c]	.380	.705	.045	.250
2	Sex Code (0=F,1=M)	-.213[d]	-2.170	.033	-.251	.550
	Age	.014[d]	.181	.857	.022	.996

Excluded Variables[a,b]

Model		Collinearity Statistics VIF	Minimum Tolerance
1	Sex Code (0=F,1=M)	1.449	.690
	Age	1.001	.999
	Height (m)	1.156	.865
	Body Mass (kg)	1.009	.991
	BMI	1.035	.966
	waist_circumference	1.000	1.000
	waist_breadth_M-L	1.022	.978
	waist_breadth_A-P	1.022	.979
	suprailiac_SF	1.351	.740
	abdomen_SF	1.680	.595
	ant_pelvis_length	1.009	.991
	lat_pelvis_length_left	1.002	.998
	AVG_lat_pelvis	1.001	.999
	pelvis_circumference	1.123	.891
	hip_circumference	1.139	.878
	hip_breadth_M-L	1.264	.791
	pelvis_breadth_A-P	1.093	.915
	left lat. thigh_length	1.189	.841
	left med. thigh_length	1.101	.908
	left mid. thigh_length	1.075	.930
	upper_thigh_circumference_L	1.217	.821
	upper_thigh_A-P_breadth_L	1.198	.835
	max-thigh_M-L_breadth_L	1.069	.936
	max-thigh_A-P_breadth_L	1.003	.997
	post_mid-thigh_SF_L	3.997	.250
2	Sex Code (0=F,1=M)	1.819	.329
	Age	1.004	.593

Excluded Variables[a,b]

Model		Beta In	t	Sig.	Partial Correlation	Collinearity. Tolerance
	Height (m)	-.287[d]	-3.827	.000	-.416	.837
	Body Mass (kg)	-.076[d]	-.927	.357	-.110	.826
	BMI	.147[d]	1.739	.086	.204	.764
	waist_circumference	-.098[d]	-1.014	.314	-.120	.600
	waist_breadth_M-L	-.192[d]	-2.087	.041	-.242	.634
	waist_breadth_A-P	-.047[d]	-.501	.618	-.060	.637
	suprailiac_SF	.154[d]	1.075	.286	.127	.274
	ant_pelvis_length	.067[d]	.893	.375	.106	.990
	lat_pelvis_length_left	-.005[d]	-.060	.952	-.007	.969
	AVG_lat_pelvis	.000[d]	-.002	.998	.000	.978
	pelvis_circumference	-.036[d]	-.353	.725	-.042	.545
	hip_circumference	.064[d]	.748	.457	.089	.775
	hip_breadth_M-L	-.003[d]	-.031	.975	-.004	.766
	pelvis_breadth_A-P	.132[d]	1.561	.123	.183	.772
	left lat. thigh_length	-.280[d]	-3.689	.000	-.403	.825
	left med. thigh_length	-.218[d]	-2.864	.006	-.324	.876
	left mid. thigh_length	.002[d]	.026	.979	.003	.921
	upper_thigh_circumference_L	.164[d]	1.962	.054	.228	.771
	upper_thigh_A-P_breadth_L	.213[d]	2.351	.022	.271	.645
	max-thigh_M-L_breadth_L	.029[d]	.368	.714	.044	.910
	max-thigh_A-P_breadth_L	.034[d]	.434	.666	.052	.899
	post_mid-thigh_SF_L	.118[d]	.784	.436	.093	.248
3	Sex Code (0=F,1=M)	.018[e]	.147	.884	.018	.329
	Age	-.009[e]	-.125	.901	-.015	.989
	Body Mass (kg)	.344[e]	3.126	.003	.352	.346
	BMI	.246[e]	3.210	.002	.360	.708
	waist_circumference	.218[e]	1.895	.062	.222	.342
	waist_breadth_M-L	.053[e]	.454	.651	.055	.349
	waist_breadth_A-P	.169[e]	1.705	.093	.201	.465
	suprailiac_SF	.179[e]	1.372	.175	.163	.273

Excluded Variables[a,b]

Model		Collinearity Statistics VIF	Minimum Tolerance
	Height (m)	1.194	.504
	Body Mass (kg)	1.210	.496
	BMI	1.310	.471
	waist_circumference	1.666	.357
	waist_breadth_M-L	1.578	.386
	waist_breadth_A-P	1.571	.387
	suprailiac_SF	3.651	.220
	ant_pelvis_length	1.011	.594
	lat_pelvis_length_left	1.032	.578
	AVG_lat_pelvis	1.022	.583
	pelvis_circumference	1.836	.364
	hip_circumference	1.290	.526
	hip_breadth_M-L	1.305	.549
	pelvis_breadth_A-P	1.295	.503
	left lat. thigh_length	1.212	.503
	left med. thigh_length	1.142	.523
	left mid. thigh_length	1.086	.553
	upper_thigh_circumference_L	1.296	.559
	upper_thigh_A-P_breadth_L	1.551	.460
	max-thigh_M-L_breadth_L	1.099	.579
	max-thigh_A-P_breadth_L	1.112	.537
	post_mid-thigh_SF_L	4.027	.202
3	Sex Code (0=F,1=M)	3.037	.322
	Age	1.011	.504
	Body Mass (kg)	2.894	.346
	BMI	1.412	.470
	waist_circumference	2.926	.316
	waist_breadth_M-L	2.865	.346
	waist_breadth_A-P	2.151	.374
	suprailiac_SF	3.660	.219

Excluded Variables[a,b]

Model		Beta In	t	Sig.	Partial Correlation	Collinearity. Tolerance
	ant_pelvis_length	.099[e]	1.444	.153	.171	.976
	lat_pelvis_length_left	.067[e]	.926	.358	.111	.907
	AVG_lat_pelvis	.069[e]	.971	.335	.116	.919
	pelvis_circumference	.250[e]	2.266	.027	.263	.366
	hip_circumference	.342[e]	3.992	.000	.433	.526
	hip_breadth_M-L	.254[e]	2.773	.007	.317	.513
	pelvis_breadth_A-P	.246[e]	3.200	.002	.359	.700
	left lat. thigh_length	-.178[e]	-2.069	.042	-.242	.604
	left med. thigh_length	-.065[e]	-.672	.504	-.081	.513
	left mid. thigh_length	.082[e]	1.103	.274	.132	.854
	upper_thigh_circumference_L	.303[e]	4.019	.000	.436	.680
	upper_thigh_A-P_breadth_L	.272[e]	3.369	.001	.376	.628
	max-thigh_M-L_breadth_L	.103[e]	1.392	.168	.165	.856
	max-thigh_A-P_breadth_L	.141[e]	1.872	.065	.220	.802
	post_mid-thigh_SF_L	-.056[e]	-.386	.700	-.046	.223
4	Sex Code (0=F,1=M)	-.035[f]	-.317	.752	-.038	.325
	Age	.011[f]	.181	.857	.022	.983
	Body Mass (kg)	-.073[f]	-.358	.721	-.043	.093
	BMI	-.029[f]	-.200	.842	-.024	.192
	waist_circumference	.005[f]	.043	.966	.005	.256
	waist_breadth_M-L	-.100[f]	-.896	.374	-.108	.310
	waist_breadth_A-P	-.026[f]	-.245	.807	-.030	.342
	suprailiac_SF	.075[f]	.608	.545	.074	.259
	ant_pelvis_length	.062[f]	.969	.336	.117	.953
	lat_pelvis_length_left	.025[f]	.369	.714	.045	.883
	AVG_lat_pelvis	.032[f]	.487	.628	.059	.899
	pelvis_circumference	.035[f]	.280	.780	.034	.257
	hip_circumference	.194[f]	1.537	.129	.183	.238
	hip_breadth_M-L	.114[f]	1.156	.252	.139	.397
	pelvis_breadth_A-P	.150[f]	1.876	.065	.222	.584

Excluded Variables[a,b]

Model		Collinearity Statistics VIF	Minimum Tolerance
	ant_pelvis_length	1.025	.504
	lat_pelvis_length_left	1.102	.504
	AVG_lat_pelvis	1.088	.503
	pelvis_circumference	2.734	.341
	hip_circumference	1.900	.448
	hip_breadth_M-L	1.950	.370
	pelvis_breadth_A-P	1.429	.498
	left lat. thigh_length	1.656	.480
	left med. thigh_length	1.951	.490
	left mid. thigh_length	1.171	.442
	upper_thigh_circumference_L	1.471	.449
	upper_thigh_A-P_breadth_L	1.592	.456
	max-thigh_M-L_breadth_L	1.169	.486
	max-thigh_A-P_breadth_L	1.247	.504
	post_mid-thigh_SF_L	4.488	.202
4	Sex Code (0=F,1=M)	3.081	.287
	Age	1.017	.448
	Body Mass (kg)	10.768	.093
	BMI	5.213	.184
	waist_circumference	3.908	.256
	waist_breadth_M-L	3.229	.310
	waist_breadth_A-P	2.925	.342
	suprailiac_SF	3.857	.218
	ant_pelvis_length	1.050	.448
	lat_pelvis_length_left	1.132	.449
	AVG_lat_pelvis	1.112	.449
	pelvis_circumference	3.892	.257
	hip_circumference	4.195	.238
	hip_breadth_M-L	2.517	.369
	pelvis_breadth_A-P	1.712	.449

Excluded Variables[a,b]

Model		Beta In	t	Sig.	Partial Correlation	Collinearity... Tolerance
	left lat. thigh_length	-.129[f]	-1.614	.111	-.192	.588
	left med. thigh_length	-.059[f]	-.671	.504	-.081	.512
	left mid. thigh_length	.062[f]	.917	.362	.111	.849
	upper_thigh_A-P_breadth_L	.102[f]	.932	.355	.112	.323
	max-thigh_M-L_breadth_L	-.019[f]	-.249	.804	-.030	.693
	max-thigh_A-P_breadth_L	-.094[f]	-.986	.328	-.119	.427
	post_mid-thigh_SF_L	-.034[f]	-.254	.800	-.031	.222

Excluded Variables[a,b]

Model		Collinearity Statistics VIF	Minimum Tolerance
	left lat. thigh_length	1.701	.438
	left med. thigh_length	1.951	.444
	left mid. thigh_length	1.178	.405
	upper_thigh_A-P_breadth_L	3.092	.323
	max-thigh_M-L_breadth_L	1.443	.448
	max-thigh_A-P_breadth_L	2.343	.362
	post_mid-thigh_SF_L	4.496	.190

a. AgeCode = .0

b. Dependent Variable: IC_Soft tissue L (cm)

c. Predictors in the Model: (Constant), ant_mid-thigh_SF_L

d. Predictors in the Model: (Constant), ant_mid-thigh_SF_L, abdomen_SF

e. Predictors in the Model: (Constant), ant_mid-thigh_SF_L, abdomen_SF, Height (m)

f. Predictors in the Model: (Constant), ant_mid-thigh_SF_L, abdomen_SF, Height (m), upper_thigh_circumference_L

Coefficient Correlations[a,b]

Model			ant_mid-thigh_SF_L	abdomen_SF	Height (m)
1	Correlations	ant_mid-thigh_SF_L	1.000		
	Covariances	ant_mid-thigh_SF_L	6.166E-5		
2	Correlations	ant_mid-thigh_SF_L	1.000	-.636	
		abdomen_SF	-.636	1.000	
	Covariances	ant_mid-thigh_SF_L	8.429E-5	-6.229E-5	
		abdomen_SF	-6.229E-5	.000	
3	Correlations	ant_mid-thigh_SF_L	1.000	-.646	.392
		abdomen_SF	-.646	1.000	-.179
		Height (m)	.392	-.179	1.000
	Covariances	ant_mid-thigh_SF_L	8.350E-5	-5.862E-5	.003
		abdomen_SF	-5.862E-5	9.854E-5	-.002
		Height (m)	.003	-.002	.750
4	Correlations	ant_mid-thigh_SF_L	1.000	-.533	.461
		abdomen_SF	-.533	1.000	-.097
		Height (m)	.461	-.097	1.000
		upper_thigh_circumference_L	-.329	-.197	-.345
	Covariances	ant_mid-thigh_SF_L	7.701E-5	-4.296E-5	.003
		abdomen_SF	-4.296E-5	8.428E-5	-.001
		Height (m)	.003	-.001	.700
		upper_thigh_circumference_L	-5.613E-5	-3.511E-5	-.006

Coefficient Correlations[a,b]

Model			upper_thigh_circumference_L
1	Correlations	ant_mid-thigh_SF_L	
	Covariances	ant_mid-thigh_SF_L	
2	Correlations	ant_mid-thigh_SF_L	
		abdomen_SF	
	Covariances	ant_mid-thigh_SF_L	
		abdomen_SF	
3	Correlations	ant_mid-thigh_SF_L	
		abdomen_SF	
		Height (m)	
	Covariances	ant_mid-thigh_SF_L	
		abdomen_SF	
		Height (m)	
4	Correlations	ant_mid-thigh_SF_L	-.329
		abdomen_SF	-.197
		Height (m)	-.345
		upper_thigh_circumference_L	1.000
	Covariances	ant_mid-thigh_SF_L	-5.613E-5
		abdomen_SF	-3.511E-5
		Height (m)	-.006
		upper_thigh_circumference_L	.000

a. AgeCode = .0

b. Dependent Variable: IC_Soft tissue L (cm)

Collinearity Diagnostics[a,b]

Model	Dimension	Eigenvalue	Condition Index	Variance Proportions (Constant)	ant_mid-thigh_SF_L	abdomen_SF
1	1	1.862	1.000	.07	.07	
	2	.138	3.675	.93	.93	
2	1	2.777	1.000	.02	.02	.02
	2	.141	4.433	.85	.37	.03
	3	.081	5.851	.13	.61	.95
3	1	3.701	1.000	.00	.01	.01
	2	.216	4.143	.00	.23	.08
	3	.082	6.716	.00	.60	.89
	4	.001	55.150	1.00	.17	.02
4	1	4.671	1.000	.00	.00	.00
	2	.243	4.386	.00	.20	.10
	3	.083	7.525	.00	.54	.84
	4	.002	44.782	.10	.12	.03
	5	.001	62.042	.90	.13	.03

Collinearity Diagnostics[a,b]

Model	Dimension	Variance Proportions Height (m)	upper_thigh_circumference_L
1	1		
	2		
2	1		
	2		
	3		
3	1	.00	
	2	.00	
	3	.00	
	4	1.00	
4	1	.00	.00
	2	.00	.00
	3	.00	.00
	4	.17	.99
	5	.83	.01

a. AgeCode = .0

b. Dependent Variable: IC_Soft tissue L (cm)

Residuals Statistics[a,b]

	Minimum	Maximum	Mean	Std. Deviation	N
Predicted Value	.4306	4.2801	2.1309	.93613	74
Std. Predicted Value	-1.816	2.296	.000	1.000	74
Standard Error of Predicted Value	.080	.289	.145	.042	74
Adjusted Predicted Value	.4103	4.1595	2.1290	.92876	74
Residual	-1.33386	1.40718	.00000	.56439	74
Std. Residual	-2.298	2.424	.000	.972	74
Stud. Residual	-2.374	2.460	.001	1.008	74
Deleted Residual	-1.42356	1.44933	.00191	.60721	74
Stud. Deleted Residual	-2.459	2.557	.001	1.022	74
Mahal. Distance	.392	17.151	3.946	3.144	74
Cook's Distance	.000	.164	.015	.027	74
Centered Leverage Value	.005	.235	.054	.043	74

a. AgeCode = .0

b. Dependent Variable: IC_Soft tissue L (cm)

Charts

Histogram

Dependent Variable: IC_Soft tissue L (cm)

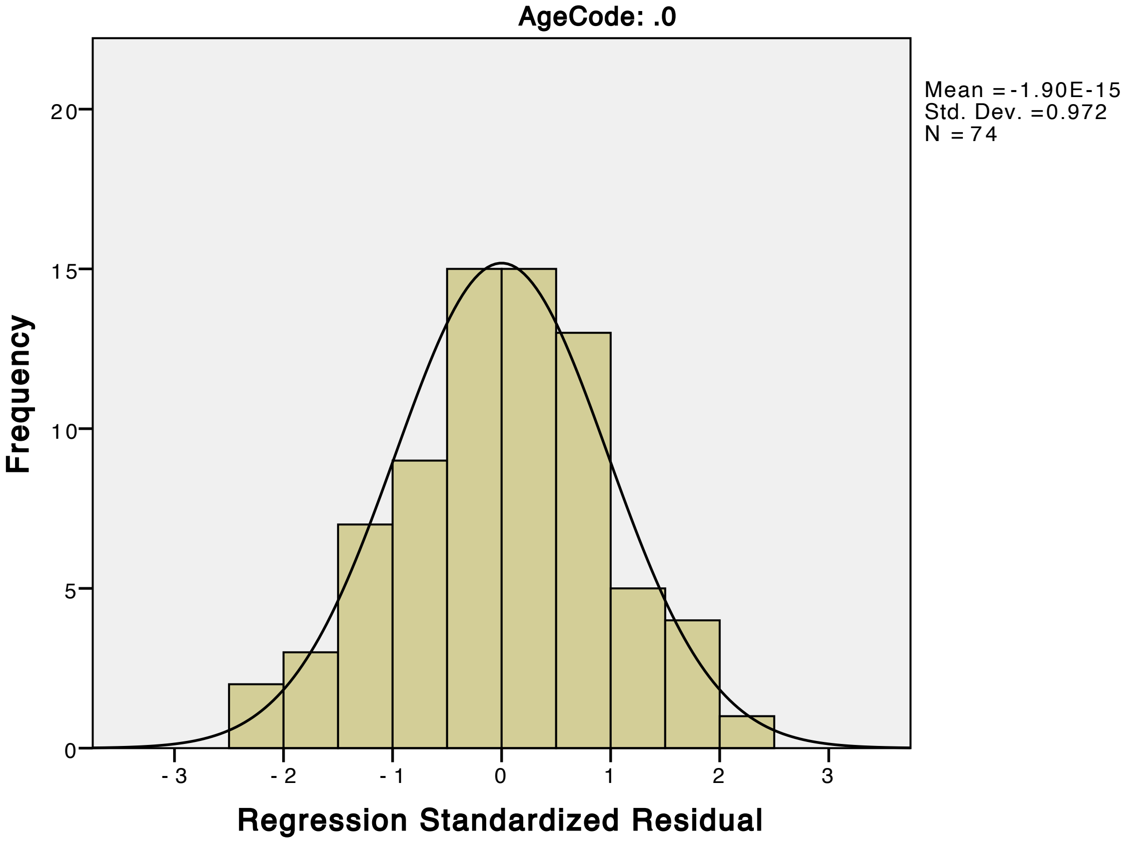

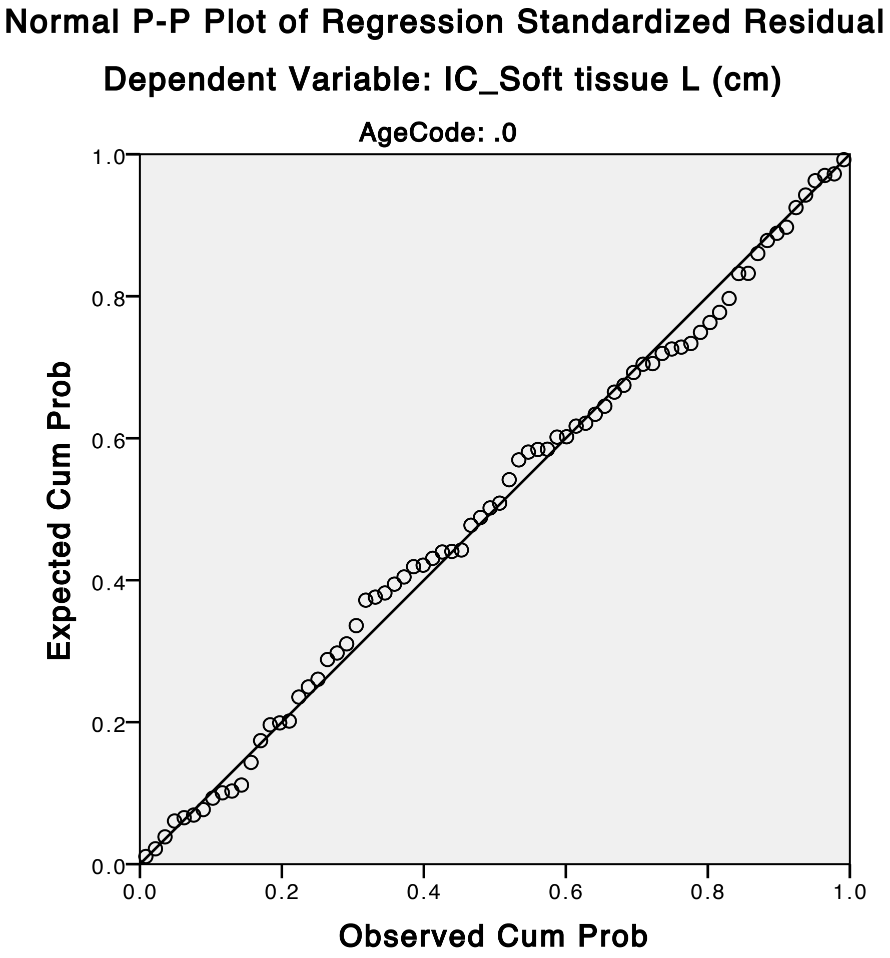
Normal P-P Plot of Regression Standardized Residual
Dependent Variable: IC_Soft tissue L (cm)
AgeCode: .0
1.0
0.8
0.6
0.4
0.2
0.0
Expected Cum Prob
0.0
0.2
0.4
0.6
0.8
1.0
Observed Cum Prob

Scatterplot

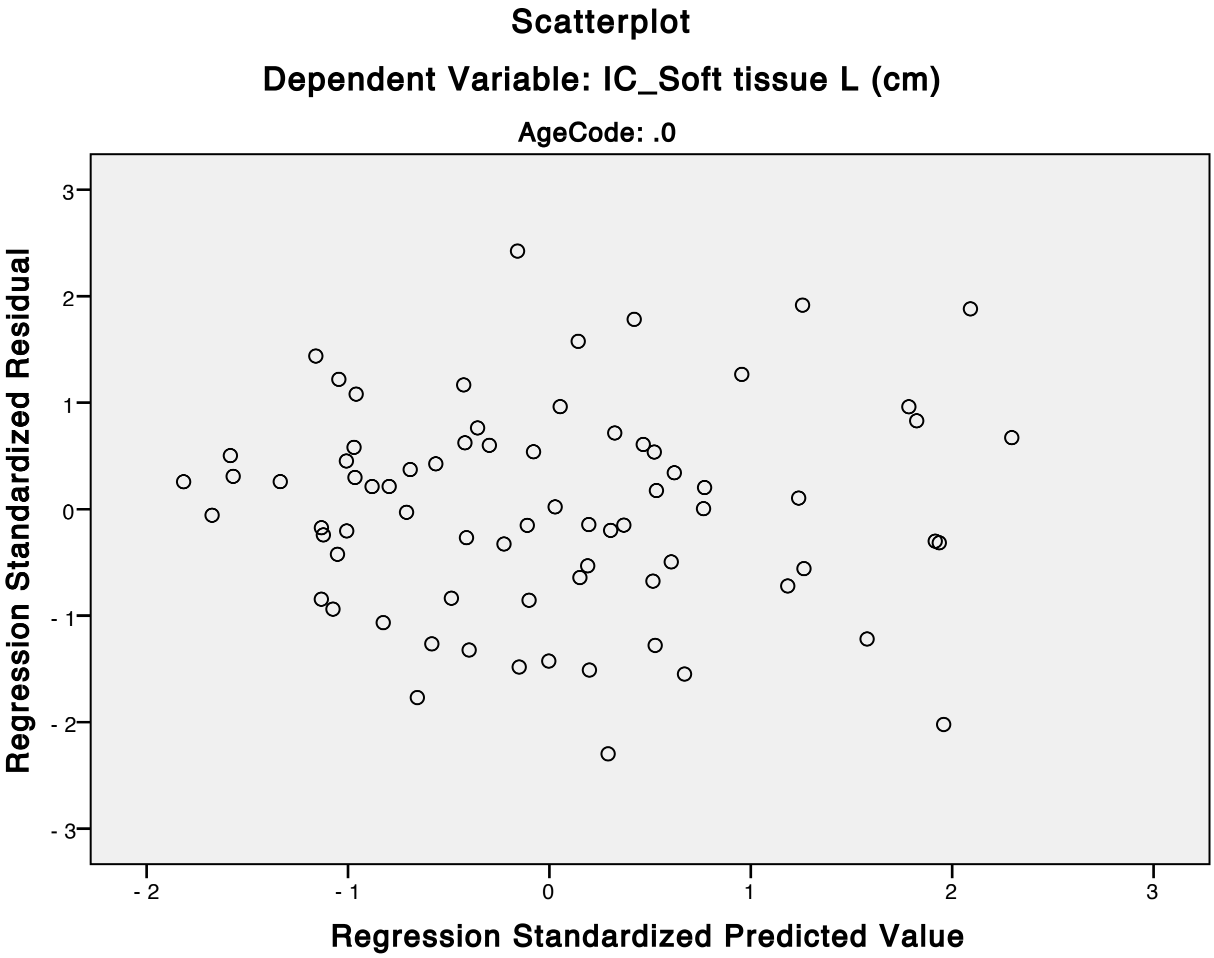

Regression

```
 REGRESSION
  /MISSING LISTWISE
  /STATISTICS COEFF OUTS BCOV R ANOVA COLLIN TOL
  /CRITERIA=PIN(.05) POUT(.10)
  /NOORIGIN
  /DEPENDENT GT_SoftTissueLcm
  /METHOD=ENTER ant_midthigh_SF_LSexCode0F1M upper_thigh_circumference_Lhip_
breadth_ML
    post_midthigh_SF_L
  /SCATTERPLOT=(*ZRESID ,*ZPRED)
  /RESIDUALS HISTOGRAM(ZRESID) NORMPROB(ZRESID)
  /CASEWISE PLOT(ZRESID) OUTLIERS(3)
  /SAVE ZPRED MAHAL COOK ZRESID.
```

```
 REGRESSION
  /MISSING LISTWISE
  /STATISTICS COEFF OUTS BCOV R ANOVA COLLIN TOL
  /CRITERIA=PIN(.05) POUT(.10)
  /NOORIGIN
  /DEPENDENT GT_SoftTissueLcm
  /METHOD=STEPWISE ant_midthigh_SF_LSexCode0F1M upper_thigh_circumference_Lh
ip_breadth_ML
  /SCATTERPLOT=(*ZRESID ,*ZPRED)
  /RESIDUALS HISTOGRAM(ZRESID) NORMPROB(ZRESID)
  /CASEWISE PLOT(ZRESID) OUTLIERS(3)
  /SAVE ZPRED MAHAL COOK ZRESID.
```

Regression

Notes

Output Created		24-NOV-2017 12:08...
Comments		
Input	Data	C: \Users\User\Desktop\Danielle FIX\Generation-Validation Groups\SPSS Data_REGRESSION_G groups final_YOUNG_no 0fm488+0mt203.sav
	Active Dataset	DataSet1
	Filter	<none>
	Weight	<none>
	Split File	<none>
	N of Rows in Working Data File	74
Missing Value Handling	Definition of Missing	User-defined missing values are treated as missing.
	Cases Used	Statistics are based on cases with no missing values for any variable used.
Syntax		REGRESSION<br>/MISSING LISTWISE<br>/STATISTICS COEFF OUTS BCOV R ANOVA COLLIN TOL<br>/CRITERIA=PIN(.05) POUT(.10)<br>/NOORIGIN<br>/DEPENDENT GT_SoftTissueLcm<br>/METHOD=STEPWISE ant_midthigh_SF_L SexCode0F1M upper_thigh_circumference_L hip_breadth_ML<br>/SCATTERPLOT=(*ZRESID ,*ZPRED)<br>/RESIDUALS HISTOGRAM(ZRESID) NORMPROB(ZRESID)<br>/CASEWISE PLOT(ZRESID) OUTLIERS(3)<br>/SAVE ZPRED MAHAL COOK ZRESID.
Resources	Processor Time	00:00:00.87
	Elapsed Time	00:00:00.33

Notes

	Memory Required	3828 bytes
	Additional Memory Required for Residual Plots	888 bytes
Variables Created or Modified	ZPR_6	Standardized Predicted Value
	ZRE_6	Standardized Residual
	MAH_6	Mahalanobis Distance
	COO_6	Cook's Distance

Variables Entered/Removed[a]

Model	Variables Entered	Variables Removed	Method
1	ant_mid-thigh_SF_L	.	Stepwise (Criteria: Probability-of-F-to-enter <= .050, Probability-of-F-to-remove >= .100).
2	Sex Code (0=F,1=M)	.	Stepwise (Criteria: Probability-of-F-to-enter <= .050, Probability-of-F-to-remove >= .100).

Variables Entered/Removed[a]

Model	Variables Entered	Variables Removed	Method
3	upper_thigh_circumference_L	.	Stepwise (Criteria: Probability-of-F-to-enter <= .050, Probability-of-F-to-remove >= .100).
4	hip_breadth_M-L	.	Stepwise (Criteria: Probability-of-F-to-enter <= .050, Probability-of-F-to-remove >= .100).

a. Dependent Variable: GT_Soft Tissue L (cm)

Model Summary[e]

Model	R	R Square	Adjusted R Square	Std. Error of the Estimate
1	.796[a]	.633	.628	.92067
2	.836[b]	.699	.691	.83897
3	.894[c]	.799	.791	.69028
4	.908[d]	.824	.814	.65118

a. Predictors: (Constant), ant_mid-thigh_SF_L

b. Predictors: (Constant), ant_mid-thigh_SF_L, Sex Code (0=F,1=M)

c. Predictors: (Constant), ant_mid-thigh_SF_L, Sex Code (0=F,1=M), upper_thigh_circumference_L

d. Predictors: (Constant), ant_mid-thigh_SF_L, Sex Code (0=F,1=M), upper_thigh_circumference_L, hip_breadth_M-L

e. Dependent Variable: GT_Soft Tissue L (cm)

ANOVA[a]

Model		Sum of Squares	df	Mean Square	F	Sig.
1	Regression	105.184	1	105.184	124.093	.000[b]
	Residual	61.029	72	.848		
	Total	166.214	73			
2	Regression	116.239	2	58.120	82.572	.000[c]
	Residual	49.975	71	.704		
	Total	166.214	73			
3	Regression	132.860	3	44.287	92.944	.000[d]
	Residual	33.354	70	.476		
	Total	166.214	73			
4	Regression	136.955	4	34.239	80.745	.000[e]
	Residual	29.259	69	.424		
	Total	166.214	73			

a. Dependent Variable: GT_Soft Tissue L (cm)

b. Predictors: (Constant), ant_mid-thigh_SF_L

c. Predictors: (Constant), ant_mid-thigh_SF_L, Sex Code (0=F,1=M)

d. Predictors: (Constant), ant_mid-thigh_SF_L, Sex Code (0=F,1=M), upper_thigh_circumference_L

e. Predictors: (Constant), ant_mid-thigh_SF_L, Sex Code (0=F,1=M), upper_thigh_circumference_L, hip_breadth_M-L

Coefficients[a]

Model		Unstandardized Coefficients B	Std. Error	Standardized Coefficients Beta	t
1	(Constant)	1.409	.211		6.670
	ant_mid-thigh_SF_L	.104	.009	.796	11.140
2	(Constant)	2.314	.299		7.746
	ant_mid-thigh_SF_L	.081	.010	.623	7.951
	Sex Code (0=F,1=M)	-.930	.235	-.310	-3.963
3	(Constant)	-4.883	1.243		-3.928
	ant_mid-thigh_SF_L	.049	.010	.378	4.933
	Sex Code (0=F,1=M)	-1.392	.208	-.464	-6.681
	upper_thigh_circumference_L	.134	.023	.376	5.906
4	(Constant)	-1.347	1.634		-.824
	ant_mid-thigh_SF_L	.064	.011	.492	6.070
	Sex Code (0=F,1=M)	-1.123	.215	-.375	-5.229
	upper_thigh_circumference_L	.171	.024	.480	6.982
	hip_breadth_M-L	-.182	.059	-.237	-3.108

Coefficients[a]

Model		Sig.	Collinearity Statistics Tolerance	VIF
1	(Constant)	.000		
	ant_mid-thigh_SF_L	.000	1.000	1.000
2	(Constant)	.000		
	ant_mid-thigh_SF_L	.000	.690	1.449
	Sex Code (0=F,1=M)	.000	.690	1.449
3	(Constant)	.000		
	ant_mid-thigh_SF_L	.000	.488	2.048
	Sex Code (0=F,1=M)	.000	.593	1.686
	upper_thigh_circumference_L	.000	.706	1.417
4	(Constant)	.413		
	ant_mid-thigh_SF_L	.000	.388	2.579
	Sex Code (0=F,1=M)	.000	.497	2.013
	upper_thigh_circumference_L	.000	.539	1.855
	hip_breadth_M-L	.003	.439	2.276

a. Dependent Variable: GT_Soft Tissue L (cm)

Excluded Variables[a]

Model		Beta In	t	Sig.	Partial Correlation	Collinearity. Tolerance
1	Sex Code (0=F,1=M)	-.310[b]	-3.963	.000	-.426	.690
	upper_thigh_circumference_L	.217[b]	2.886	.005	.324	.821
	hip_breadth_M-L	-.136[b]	-1.710	.092	-.199	.791
2	upper_thigh_circumference_L	.376[c]	5.906	.000	.577	.706
	hip_breadth_M-L	.022[c]	.253	.801	.030	.575
3	hip_breadth_M-L	-.237[d]	-3.108	.003	-.350	.439

Excluded Variables[a]

Model		Collinearity Statistics VIF	Minimum Tolerance
1	Sex Code (0=F,1=M)	1.449	.690
	upper_thigh_circumference_L	1.217	.821
	hip_breadth_M-L	1.264	.791
2	upper_thigh_circumference_L	1.417	.488
	hip_breadth_M-L	1.738	.404
3	hip_breadth_M-L	2.276	.388

a. Dependent Variable: GT_Soft Tissue L (cm)

b. Predictors in the Model: (Constant), ant_mid-thigh_SF_L

c. Predictors in the Model: (Constant), ant_mid-thigh_SF_L, Sex Code (0=F,1=M)

d. Predictors in the Model: (Constant), ant_mid-thigh_SF_L, Sex Code (0=F,1=M), upper_thigh_circumference_L

Coefficient Correlations[a]

Model			ant_mid-thigh_SF_L	Sex Code (0=F,1=M)	upper_thigh_circumference_L
1	Correlations	ant_mid-thigh_SF_L	1.000		
	Covariances	ant_mid-thigh_SF_L	8.701E-5		
2	Correlations	ant_mid-thigh_SF_L	1.000	.556	
		Sex Code (0=F,1=M)	.556	1.000	
	Covariances	ant_mid-thigh_SF_L	.000	.001	
		Sex Code (0=F,1=M)	.001	.055	
3	Correlations	ant_mid-thigh_SF_L	1.000	.637	-.541
		Sex Code (0=F,1=M)	.637	1.000	-.375
		upper_thigh_circumference_L	-.541	-.375	1.000
	Covariances	ant_mid-thigh_SF_L	.000	.001	.000
		Sex Code (0=F,1=M)	.001	.043	-.002
		upper_thigh_circumference_L	.000	-.002	.001
4	Correlations	ant_mid-thigh_SF_L	1.000	.702	-.201
		Sex Code (0=F,1=M)	.702	1.000	-.104
		upper_thigh_circumference_L	-.201	-.104	1.000
		hip_breadth_M-L	-.454	-.403	-.486
	Covariances	ant_mid-thigh_SF_L	.000	.002	-5.201E-5
		Sex Code (0=F,1=M)	.002	.046	-.001
		upper_thigh_circumference_L	-5.201E-5	-.001	.001
		hip_breadth_M-L	.000	-.005	-.001

Coefficient Correlations[a]

Model			hip_breadth_M-L
1	Correlations	ant_mid-thigh_SF_L	
	Covariances	ant_mid-thigh_SF_L	
2	Correlations	ant_mid-thigh_SF_L	
		Sex Code (0=F,1=M)	
	Covariances	ant_mid-thigh_SF_L	
		Sex Code (0=F,1=M)	
3	Correlations	ant_mid-thigh_SF_L	
		Sex Code (0=F,1=M)	
		upper_thigh_circumference_L	
	Covariances	ant_mid-thigh_SF_L	
		Sex Code (0=F,1=M)	
		upper_thigh_circumference_L	
4	Correlations	ant_mid-thigh_SF_L	-.454
		Sex Code (0=F,1=M)	-.403
		upper_thigh_circumference_L	-.486
		hip_breadth_M-L	1.000
	Covariances	ant_mid-thigh_SF_L	.000
		Sex Code (0=F,1=M)	-.005
		upper_thigh_circumference_L	-.001
		hip_breadth_M-L	.003

a. Dependent Variable: GT_Soft Tissue L (cm)

Collinearity Diagnostics[a]

Model	Dimension	Eigenvalue	Condition Index	Variance Proportions (Constant)	ant_mid-thigh_SF_L	Sex Code (0=F,1=M)
1	1	1.862	1.000	.07	.07	
	2	.138	3.675	.93	.93	
2	1	2.336	1.000	.02	.02	.04
	2	.601	1.972	.00	.11	.36
	3	.063	6.081	.98	.87	.60
3	1	3.313	1.000	.00	.01	.02
	2	.605	2.340	.00	.07	.33
	3	.080	6.422	.01	.70	.55
	4	.002	41.785	.99	.23	.10
4	1	4.301	1.000	.00	.00	.01
	2	.607	2.662	.00	.05	.28
	3	.089	6.944	.00	.58	.48
	4	.002	47.127	.36	.13	.05
	5	.001	66.363	.63	.24	.17

Collinearity Diagnostics[a]

Model	Dimension	Variance Proportions upper_thigh_circumference_L	hip_breadth_M-L
1	1		
	2		
2	1		
	2		
	3		
3	1	.00	
	2	.00	
	3	.01	
	4	.99	
4	1	.00	.00
	2	.00	.00
	3	.00	.00
	4	.87	.02
	5	.13	.98

a. Dependent Variable: GT_Soft Tissue L (cm)

Residuals Statistics[a]

	Minimum	Maximum	Mean	Std. Deviation	N
Predicted Value	1.0910	7.2606	3.4370	1.36971	74
Std. Predicted Value	-1.713	2.792	.000	1.000	74
Standard Error of Predicted Value	.113	.307	.165	.037	74
Adjusted Predicted Value	1.0865	7.4034	3.4333	1.37285	74
Residual	-1.55596	1.41727	.00000	.63309	74
Std. Residual	-2.389	2.176	.000	.972	74
Stud. Residual	-2.454	2.305	.003	1.010	74
Deleted Residual	-1.64145	1.58973	.00377	.68413	74
Stud. Deleted Residual	-2.550	2.382	.002	1.024	74
Mahal. Distance	1.228	15.223	3.946	2.389	74
Cook's Distance	.000	.129	.016	.026	74
Centered Leverage Value	.017	.209	.054	.033	74

a. Dependent Variable: GT_Soft Tissue L (cm)

Charts

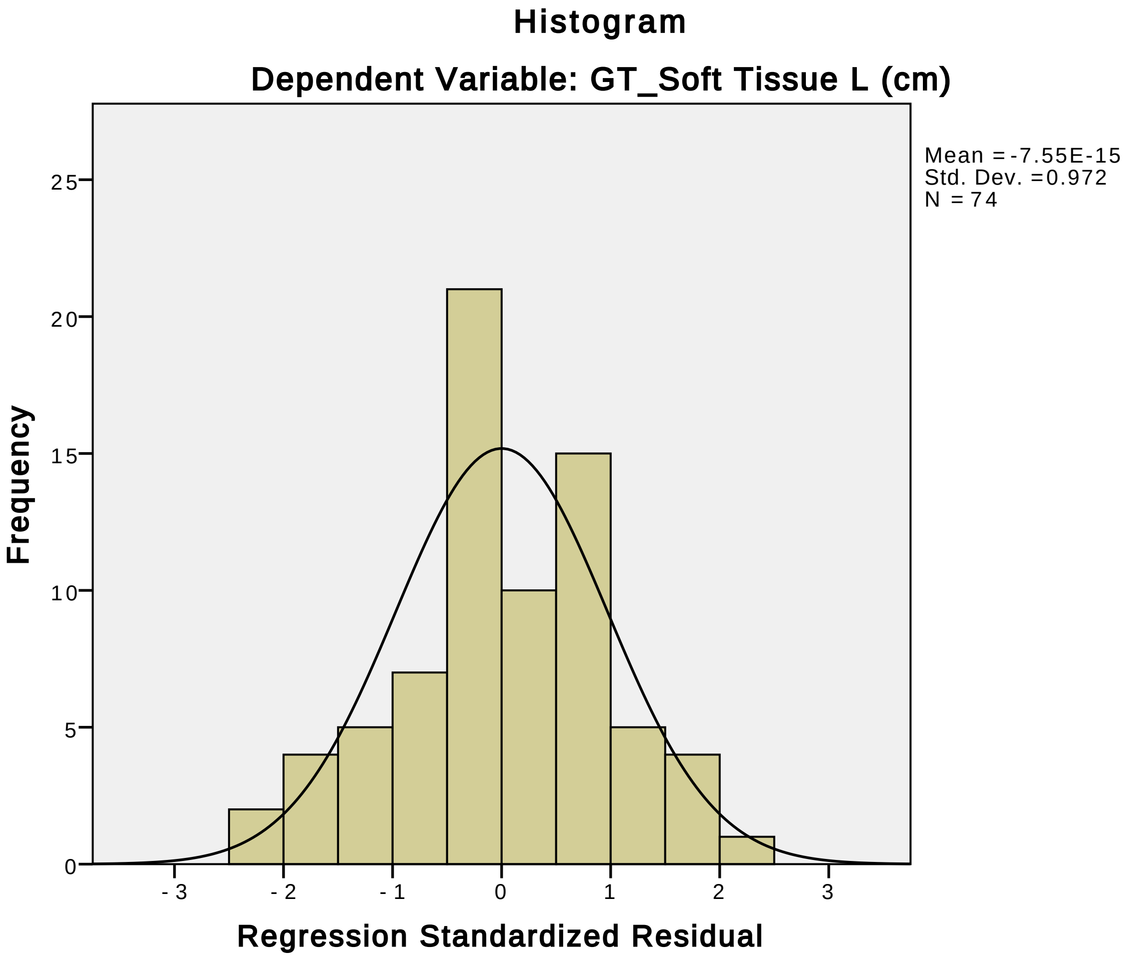
Histogram
Dependent Variable: GT_Soft Tissue L (cm)
Mean =-7.55E-15
Std. Dev. =0.972
N = 74
Frequency
Regression Standardized Residual
25
20
15
10
5
0
-3
-2
-1
0
1
2
3

Normal P-P Plot of Regression Standardized Residual

Dependent Variable: GT_Soft Tissue L (cm)

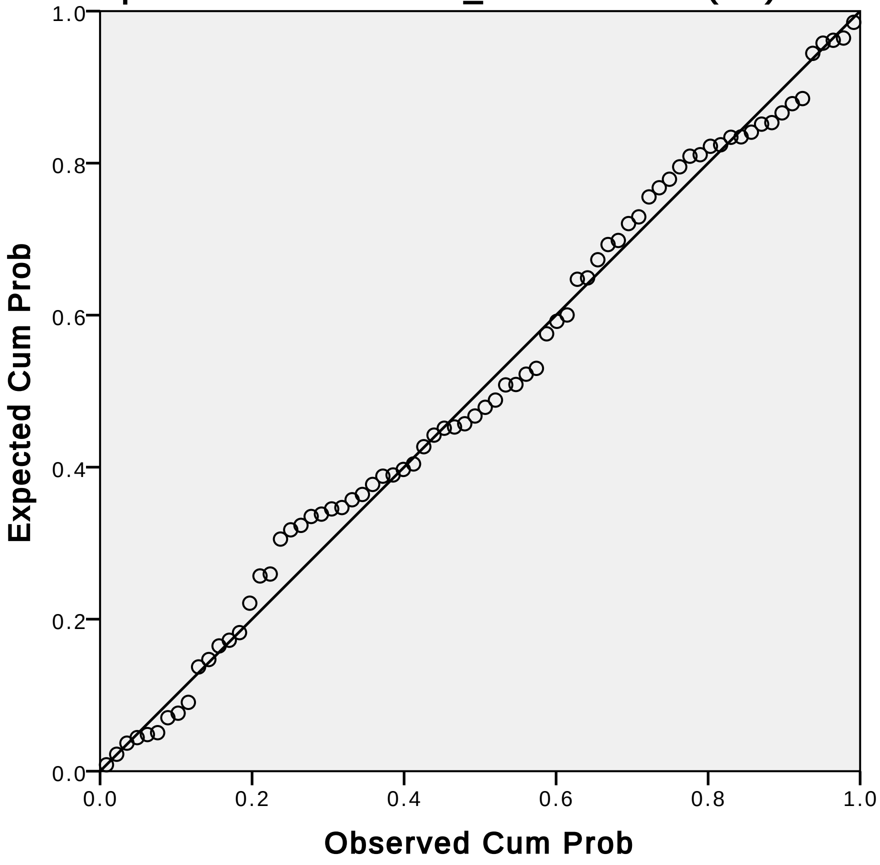

Scatterplot

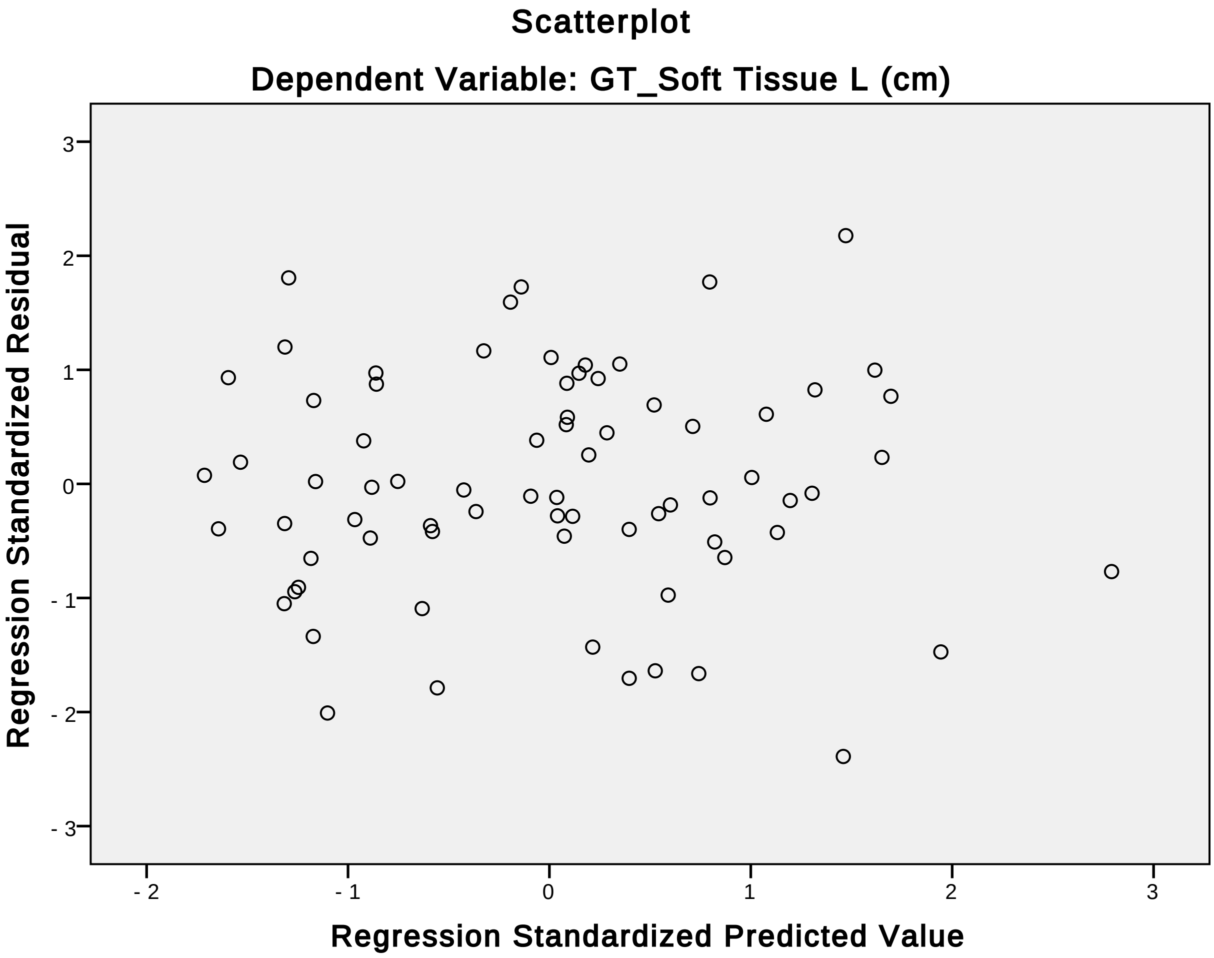


```
REGRESSION
  /MISSING LISTWISE
  /STATISTICS COEFF OUTS BCOV R ANOVA COLLIN TOL
  /CRITERIA=PIN(.05) POUT(.10)
  /NOORIGIN
  /DEPENDENT GT_SoftTissueRcm
  /METHOD=STEPWISE SexCode0F1M Heightm ant_midthigh_SF_Rupper_thigh_circumfer
ence_R
  /SCATTERPLOT=(*ZRESID ,*ZPRED)
  /RESIDUALS HISTOGRAM(ZRESID) NORMPROB(ZRESID)
  /CASEWISE PLOT(ZRESID) OUTLIERS(3)
  /SAVE ZPRED MAHAL COOK ZRESID.
```

Regression

Notes

Output Created		24-NOV-2017 12:12...
Comments		
Input	Data	C: \Users\User\Desktop\Danielle FIX\Generation-Validation Groups\SPSS Data_REGRESSION_G groups final_YOUNG_no 0fm488+0mt203.sav
	Active Dataset	DataSet1
	Filter	<none>
	Weight	<none>
	Split File	<none>
	N of Rows in Working Data File	74
Missing Value Handling	Definition of Missing	User-defined missing values are treated as missing.
	Cases Used	Statistics are based on cases with no missing values for any variable used.
Syntax		REGRESSION<br>/MISSING LISTWISE<br>/STATISTICS COEFF OUTS BCOV R ANOVA COLLIN TOL<br>/CRITERIA=PIN(.05) POUT(.10)<br>/NOORIGIN<br>/DEPENDENT GT_SoftTissueRcm<br>/METHOD=STEPWISE SexCode0F1M Heightm ant_midthigh_SF_R upper_thigh_circumference_R<br>/SCATTERPLOT=(*ZRESID ,*ZPRED)<br>/RESIDUALS HISTOGRAM(ZRESID) NORMPROB(ZRESID)<br>/CASEWISE PLOT(ZRESID) OUTLIERS(3)<br>/SAVE ZPRED MAHAL COOK ZRESID.
Resources	Processor Time	00:00:00.48
	Elapsed Time	00:00:00.32

Notes

	Memory Required	3908 bytes
	Additional Memory Required for Residual Plots	888 bytes
Variables Created or Modified	ZPR_7	Standardized Predicted Value
	ZRE_7	Standardized Residual
	MAH_7	Mahalanobis Distance
	COO_7	Cook's Distance

Variables Entered/Removed[a]

Model	Variables Entered	Variables Removed	Method
1	ant_mid-thigh_SF_R	.	Stepwise (Criteria: Probability-of-F-to-enter <= .050, Probability-of-F-to-remove >= .100).
2	upper_thigh_circumference_R	.	Stepwise (Criteria: Probability-of-F-to-enter <= .050, Probability-of-F-to-remove >= .100).

Variables Entered/Removed[a]

Model	Variables Entered	Variables Removed	Method
3	Sex Code (0=F,1=M)	.	Stepwise (Criteria: Probability-of-F-to-enter <= .050, Probability-of-F-to-remove >= .100).
4	Height (m)	.	Stepwise (Criteria: Probability-of-F-to-enter <= .050, Probability-of-F-to-remove >= .100).

a. Dependent Variable: GT_Soft Tissue R (cm)

Model Summary[e]

Model	R	R Square	Adjusted R Square	Std. Error of the Estimate
1	.727[a]	.529	.523	1.06724
2	.783[b]	.613	.602	.97475
3	.872[c]	.760	.749	.77316
4	.884[d]	.782	.769	.74245

a. Predictors: (Constant), ant_mid-thigh_SF_R

b. Predictors: (Constant), ant_mid-thigh_SF_R, upper_thigh_circumference_R

c. Predictors: (Constant), ant_mid-thigh_SF_R, upper_thigh_circumference_R, Sex Code (0=F,1=M)

d. Predictors: (Constant), ant_mid-thigh_SF_R, upper_thigh_circumference_R, Sex Code (0=F,1=M), Height (m)

e. Dependent Variable: GT_Soft Tissue R (cm)

ANOVA[a]

Model		Sum of Squares	df	Mean Square	F	Sig.
1	Regression	92.185	1	92.185	80.935	.000[b]
	Residual	82.008	72	1.139		
	Total	174.193	73			
2	Regression	106.733	2	53.366	56.167	.000[c]
	Residual	67.460	71	.950		
	Total	174.193	73			
3	Regression	132.349	3	44.116	73.801	.000[d]
	Residual	41.844	70	.598		
	Total	174.193	73			
4	Regression	136.158	4	34.039	61.751	.000[e]
	Residual	38.035	69	.551		
	Total	174.193	73			

a. Dependent Variable: GT_Soft Tissue R (cm)

b. Predictors: (Constant), ant_mid-thigh_SF_R

c. Predictors: (Constant), ant_mid-thigh_SF_R, upper_thigh_circumference_R

d. Predictors: (Constant), ant_mid-thigh_SF_R, upper_thigh_circumference_R, Sex Code (0=F,1=M)

e. Predictors: (Constant), ant_mid-thigh_SF_R, upper_thigh_circumference_R, Sex Code (0=F,1=M), Height (m)

Coefficients[a]

Model		Unstandardized Coefficients B	Unstandardized Coefficients Std. Error	Standardized Coefficients Beta	t
1	(Constant)	1.932	.239		8.074
	ant_mid-thigh_SF_R	.097	.011	.727	8.996
2	(Constant)	-4.265	1.599		-2.668
	ant_mid-thigh_SF_R	.082	.011	.617	7.807
	upper_thigh_circumference_R	.107	.027	.309	3.913
3	(Constant)	-6.176	1.301		-4.747
	ant_mid-thigh_SF_R	.038	.011	.284	3.523
	upper_thigh_circumference_R	.165	.023	.477	7.044
	Sex Code (0=F,1=M)	-1.522	.233	-.496	-6.546
4	(Constant)	-1.004	2.331		-.431
	ant_mid-thigh_SF_R	.037	.010	.278	3.591
	upper_thigh_circumference_R	.172	.023	.499	7.609
	Sex Code (0=F,1=M)	-1.095	.276	-.357	-3.961
	Height (m)	-3.397	1.292	-.208	-2.629

Coefficients[a]

Model		Sig.	Collinearity Statistics Tolerance	VIF
1	(Constant)	.000		
	ant_mid-thigh_SF_R	.000	1.000	1.000
2	(Constant)	.009		
	ant_mid-thigh_SF_R	.000	.873	1.146
	upper_thigh_circumference_R	.000	.873	1.146
3	(Constant)	.000		
	ant_mid-thigh_SF_R	.001	.527	1.899
	upper_thigh_circumference_R	.000	.748	1.337
	Sex Code (0=F,1=M)	.000	.597	1.674
4	(Constant)	.668		
	ant_mid-thigh_SF_R	.001	.526	1.900
	upper_thigh_circumference_R	.000	.736	1.359
	Sex Code (0=F,1=M)	.000	.390	2.563
	Height (m)	.011	.506	1.977

a. Dependent Variable: GT_Soft Tissue R (cm)

Excluded Variables[a]

Model		Beta In	t	Sig.	Partial Correlation	Collinearity. Tolerance
1	Sex Code (0=F,1=M)	-.294[b]	-3.229	.002	-.358	.697
	Height (m)	-.246[b]	-2.993	.004	-.335	.870
	upper_thigh_circumference_R	.309[b]	3.913	.000	.421	.873
2	Sex Code (0=F,1=M)	-.496[c]	-6.546	.000	-.616	.597
	Height (m)	-.392[c]	-5.582	.000	-.555	.774
3	Height (m)	-.208[d]	-2.629	.011	-.302	.506

Excluded Variables[a]

Model		Collinearity Statistics VIF	Minimum Tolerance
1	Sex Code (0=F,1=M)	1.434	.697
	Height (m)	1.149	.870
	upper_thigh_circumference_R	1.146	.873
2	Sex Code (0=F,1=M)	1.674	.527
	Height (m)	1.291	.694
3	Height (m)	1.977	.390

a. Dependent Variable: GT_Soft Tissue R (cm)

b. Predictors in the Model: (Constant), ant_mid-thigh_SF_R

c. Predictors in the Model: (Constant), ant_mid-thigh_SF_R, upper_thigh_circumference_R

d. Predictors in the Model: (Constant), ant_mid-thigh_SF_R, upper_thigh_circumference_R, Sex Code (0=F,1=M)

Coefficient Correlations[a]

Model			ant_mid-thigh_SF_R	upper_thigh_circumference_R	Sex Code (0=F,1=M)
1	Correlations	ant_mid-thigh_SF_R	1.000		
	Covariances	ant_mid-thigh_SF_R	.000		
2	Correlations	ant_mid-thigh_SF_R	1.000	-.357	
		upper_thigh_circumference_R	-.357	1.000	
	Covariances	ant_mid-thigh_SF_R	.000	.000	
		upper_thigh_circumference_R	.000	.001	
3	Correlations	ant_mid-thigh_SF_R	1.000	-.495	.630
		upper_thigh_circumference_R	-.495	1.000	-.379
		Sex Code (0=F,1=M)	.630	-.379	1.000
	Covariances	ant_mid-thigh_SF_R	.000	.000	.002
		upper_thigh_circumference_R	.000	.001	-.002
		Sex Code (0=F,1=M)	.002	-.002	.054
4	Correlations	ant_mid-thigh_SF_R	1.000	-.494	.492
		upper_thigh_circumference_R	-.494	1.000	-.229

Coefficient Correlations[a]

Model			Height (m)
1	Correlations	ant_mid-thigh_SF_R	
	Covariances	ant_mid-thigh_SF_R	
2	Correlations	ant_mid-thigh_SF_R	
		upper_thigh_circumference_R	
	Covariances	ant_mid-thigh_SF_R	
		upper_thigh_circumference_R	
3	Correlations	ant_mid-thigh_SF_R	
		upper_thigh_circumference_R	
		Sex Code (0=F,1=M)	
	Covariances	ant_mid-thigh_SF_R	
		upper_thigh_circumference_R	
		Sex Code (0=F,1=M)	
4	Correlations	ant_mid-thigh_SF_R	.029
		upper_thigh_circumference_R	-.127

Coefficient Correlations[a]

Model			ant_mid-thigh_SF_R	upper_thigh_circumference_R	Sex Code (0=F,1=M)
		Sex Code (0=F,1=M)	.492	-.229	1.000
		Height (m)	.029	-.127	-.589
	Covariances	ant_mid-thigh_SF_R	.000	.000	.001
		upper_thigh_circumference_R	.000	.001	-.001
		Sex Code (0=F,1=M)	.001	-.001	.076
		Height (m)	.000	-.004	-.210

Coefficient Correlations[a]

Model			Height (m)
		Sex Code (0=F,1=M)	-.589
		Height (m)	1.000
	Covariances	ant_mid-thigh_SF_R	.000
		upper_thigh_circumference_R	-.004
		Sex Code (0=F,1=M)	-.210
		Height (m)	1.670

a. Dependent Variable: GT_Soft Tissue R (cm)

Collinearity Diagnostics[a]

Model	Dimension	Eigenvalue	Condition Index	Variance Proportions (Constant)	ant_mid-thigh_SF_R	upper_thigh_circumference_R
1	1	1.855	1.000	.07	.07	
	2	.145	3.577	.93	.93	
2	1	2.814	1.000	.00	.03	.00
	2	.184	3.916	.00	.88	.00
	3	.002	34.047	.99	.09	1.00
3	1	3.302	1.000	.00	.01	.00
	2	.610	2.326	.00	.08	.00
	3	.085	6.240	.01	.73	.01
	4	.002	38.832	.99	.18	.99
4	1	4.287	1.000	.00	.01	.00
	2	.611	2.650	.00	.08	.00
	3	.098	6.599	.00	.71	.00
	4	.003	38.847	.05	.20	.95
	5	.001	74.480	.95	.01	.04

Collinearity Diagnostics[a]

Model	Dimension	Variance Proportions: Sex Code (0=F,1=M)	Variance Proportions: Height (m)
1	1		
	2		
2	1		
	2		
	3		
3	1	.02	
	2	.33	
	3	.56	
	4	.10	
4	1	.01	.00
	2	.21	.00
	3	.39	.00
	4	.00	.11
	5	.39	.89

a. Dependent Variable: GT_Soft Tissue R (cm)

Casewise Diagnostics[a]

Case Number	Std. Residual	GT_Soft Tissue R (cm)	Predicted Value	Residual
31	3.053	8.13	5.8636	2.26641

a. Dependent Variable: GT_Soft Tissue R (cm)

Residuals Statistics[a]

	Minimum	Maximum	Mean	Std. Deviation	N
Predicted Value	1.2423	8.0872	3.7723	1.36571	74
Std. Predicted Value	-1.852	3.159	.000	1.000	74
Standard Error of Predicted Value	.126	.347	.188	.045	74
Adjusted Predicted Value	1.3162	8.4571	3.7729	1.37710	74
Residual	-1.84773	2.26641	.00000	.72182	74
Std. Residual	-2.489	3.053	.000	.972	74
Stud. Residual	-2.595	3.189	.000	1.011	74
Deleted Residual	-2.00923	2.47275	-.00065	.78128	74
Stud. Deleted Residual	-2.712	3.428	-.001	1.035	74
Mahal. Distance	1.100	14.922	3.946	2.562	74
Cook's Distance	.000	.228	.017	.038	74
Centered Leverage Value	.015	.204	.054	.035	74

a. Dependent Variable: GT_Soft Tissue R (cm)

Charts

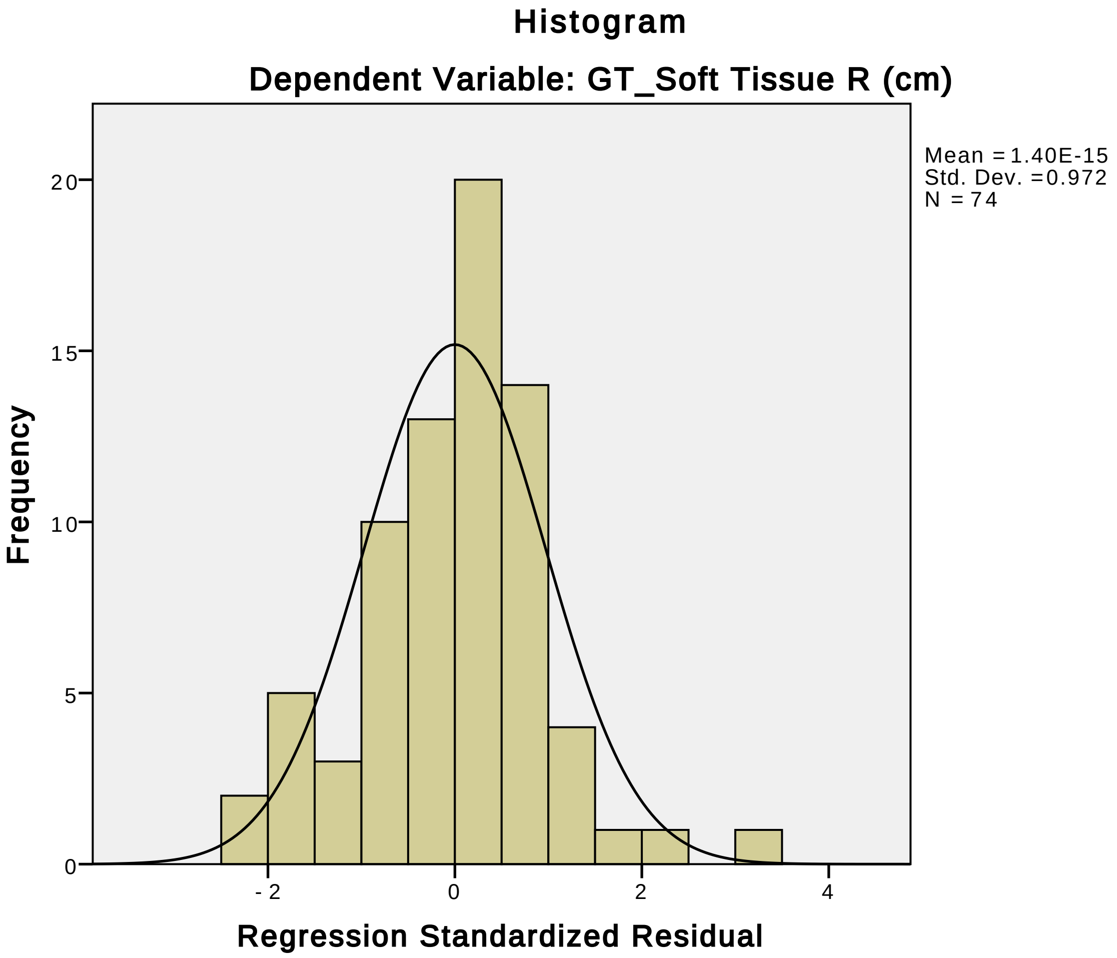
Histogram
Dependent Variable: GT_Soft Tissue R (cm)
Mean = 1.40E-15
Std. Dev. = 0.972
N = 74
Frequency
0
5
10
15
20
-2
0
2
4
Regression Standardized Residual

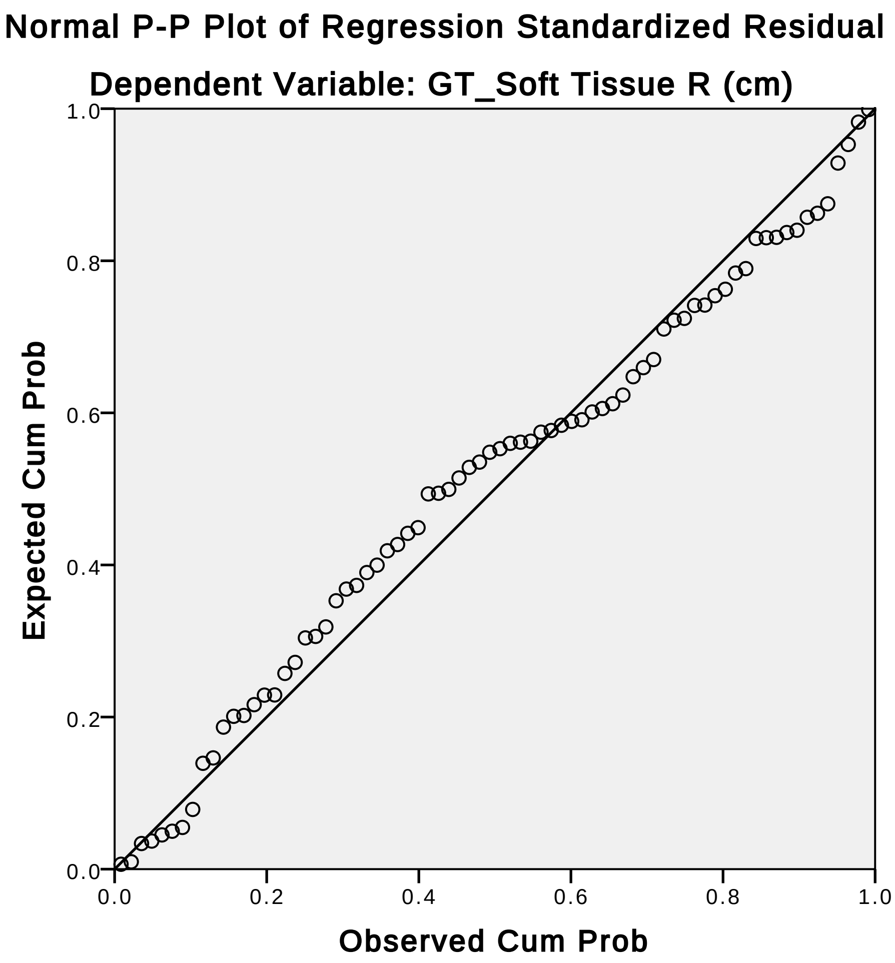
Normal P-P Plot of Regression Standardized Residual
Dependent Variable: GT_Soft Tissue R (cm)
Expected Cum Prob
Observed Cum Prob
0.0
0.2
0.4
0.6
0.8
1.0

Scatterplot

Dependent Variable: GT_Soft Tissue R (cm)

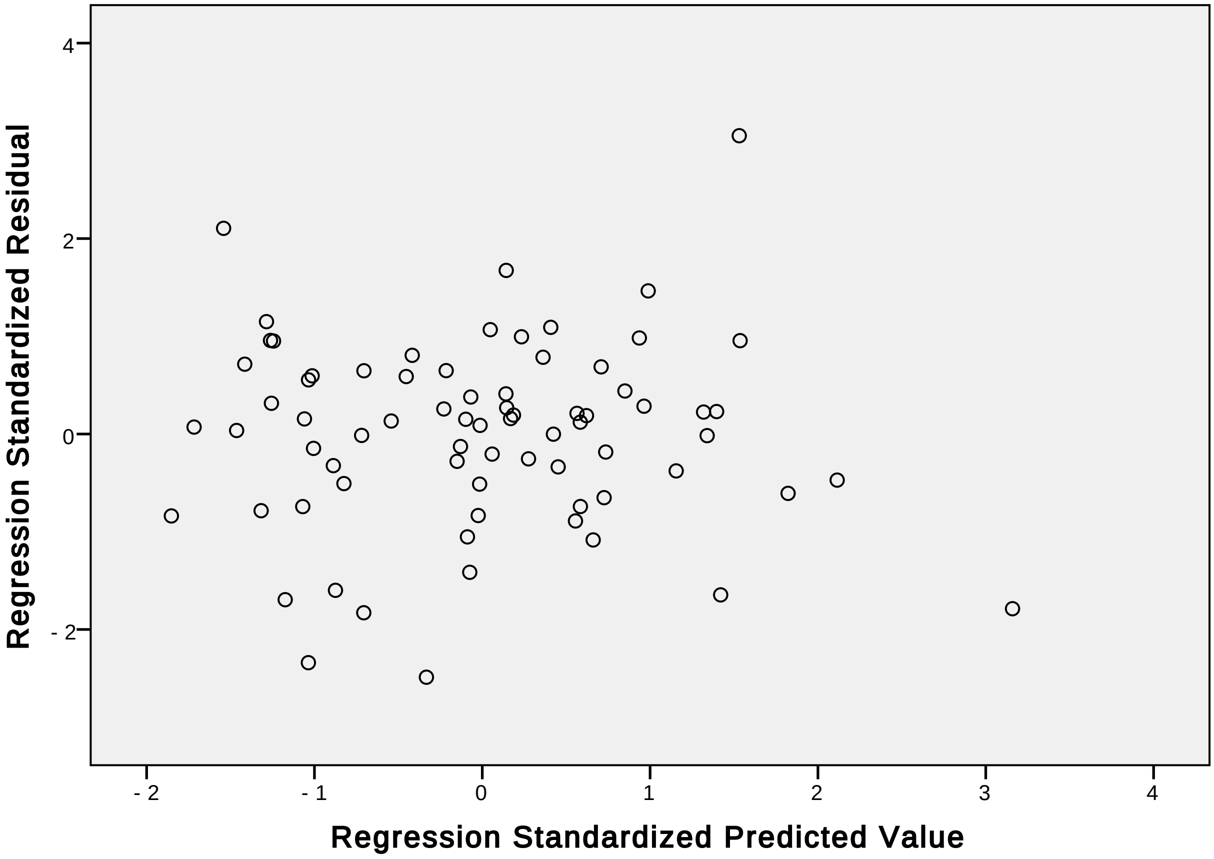


```
REGRESSION
  /MISSING LISTWISE
  /STATISTICS COEFF OUTS BCOV R ANOVA COLLIN TOL
  /CRITERIA=PIN(.05) POUT(.10)
  /NOORIGIN
  /DEPENDENT GT_SoftTissueRcm
  /METHOD=STEPWISE SexCode0F1M ant_midthigh_SF_Rupper_thigh_circumference_R
  /SCATTERPLOT=(*ZRESID ,*ZPRED)
  /RESIDUALS HISTOGRAM(ZRESID) NORMPROB(ZRESID)
  /CASEWISE PLOT(ZRESID) OUTLIERS(3)
  /SAVE ZPRED MAHAL COOK ZRESID.
```

Your license renewal date has passed. This product will stop working if a new license is not installed soon.
