# Supplementary material for: Predicting soft tissue thicknesses overlying the iliac crests and greater trochanters of younger and older adults
Source: PLoS One. 2023 Mar 14;18(3):e0283012. doi: 10.1371/journal.pone.0283012 (PMC10013917; doi:10.1371/journal.pone.0283012)

Regression

Notes

Output Created		21-NOV-2017 12:51:54
Comments		
Input	Data	F:\Claudia Regression\Danielle FIX\Generation-Validation Groups\SPSS Data_REGRESSION_G groups final_OLDER_no 0fm488+0mt203.sav
	Active Dataset	DataSet3
	Filter	<none>
	Weight	<none>
	Split File	<none>
	N of Rows in Working Data File	76
Missing Value Handling	Definition of Missing	User-defined missing values are treated as missing.
	Cases Used	Statistics are based on cases with no missing values for any variable used.

Notes

Syntax		REGRESSION<br>/MISSING LISTWISE<br>/STATISTICS COEFF OUTS BCOV R ANOVA COLLIN TOL<br>/CRITERIA=PIN(.05) POUT(.10)<br>/NOORIGIN<br>/DEPENDENT ICSofttissueRcm<br>/METHOD=STEPWISE Age SexCode0F1M Heightm BodyMasskg waist_circumference waist_breadth_ML<br>waist_breadth_AP suprailiac_SF abdomen_SF ant_pelvis_length lat_pelvis_length_right AVG_lat_pelvis<br>hip_circumference hip_breadth_ML pelvis_breadth_AP rightlat.thigh_length rightmed.thigh_length<br>rightmid.thigh_length upper_thigh_circumference_R midthigh_circumference_R upperthigh_AP_breadth_R<br><br>maxthigh_ML_breadth_R maxthigh_AP_breadth_R ant_midthigh_SF_R post_midthigh_SF_R<br>/SCATTERPLOT=(*ZRESID ,*ZPRED)<br>/RESIDUALS HISTOGRAM(ZRESID) NORMPROB(ZRESID)<br>/CASEWISE PLOT(ZRESID) OUTLIERS(3)<br>/SAVE ZPRED MAHAL COOK ZRESID.
Resources	Processor Time	00:00:00.33
	Elapsed Time	00:00:00.31

Notes

	Memory Required	19524 bytes
	Additional Memory Required for Residual Plots	720 bytes
Variables Created or Modified	ZPR_8	Standardized Predicted Value
	ZRE_8	Standardized Residual
	MAH_8	Mahalanobis Distance
	COO_8	Cook's Distance

Variables Entered/Removed[a]

Model	Variables Entered	Variables Removed	Method
1	hip_circumference	.	Stepwise (Criteria: Probability-of-F-to-enter <= .050, Probability-of-F-to-remove >= .100).
2	Sex Code (0=F,1=M)	.	Stepwise (Criteria: Probability-of-F-to-enter <= .050, Probability-of-F-to-remove >= .100).
3	Height (m)	.	Stepwise (Criteria: Probability-of-F-to-enter <= .050, Probability-of-F-to-remove >= .100).
4	abdomen_SF	.	Stepwise (Criteria: Probability-of-F-to-enter <= .050, Probability-of-F-to-remove >= .100).

a. Dependent Variable: ICSoft tissue R (cm)

Model Summary[e]

Model	R	R Square	Adjusted R Square	Std. Error of the Estimate
1	.734[a]	.539	.533	1.34398
2	.890[b]	.791	.786	.91040
3	.905[c]	.819	.811	.85450
4	.914[d]	.835	.825	.82142

a. Predictors: (Constant), hip_circumference

b. Predictors: (Constant), hip_circumference, Sex Code (0=F,1=M)

c. Predictors: (Constant), hip_circumference, Sex Code (0=F,1=M), Height (m)

d. Predictors: (Constant), hip_circumference, Sex Code (0=F,1=M), Height (m), abdomen_SF

e. Dependent Variable: ICSoft tissue R (cm)

ANOVA[a]

Model		Sum of Squares	df	Mean Square	F	Sig.
1	Regression	156.180	1	156.180	86.465	.000[b]
	Residual	133.665	74	1.806		
	Total	289.845	75			
2	Regression	229.341	2	114.670	138.352	.000[c]
	Residual	60.505	73	.829		
	Total	289.845	75			
3	Regression	237.272	3	79.091	108.317	.000[d]
	Residual	52.573	72	.730		
	Total	289.845	75			
4	Regression	241.939	4	60.485	89.643	.000[e]
	Residual	47.906	71	.675		
	Total	289.845	75			

a. Dependent Variable: ICSoft tissue R (cm)

b. Predictors: (Constant), hip_circumference

c. Predictors: (Constant), hip_circumference, Sex Code (0=F,1=M)

d. Predictors: (Constant), hip_circumference, Sex Code (0=F,1=M), Height (m)

e. Predictors: (Constant), hip_circumference, Sex Code (0=F,1=M), Height (m), abdomen_SF

Coefficients[a]

Model		Unstandardized Coefficients B	Unstandardized Coefficients Std. Error	Standardized Coefficients Beta	t	Sig.
1	(Constant)	-13.325	1.882		-7.079	.000
	hip_circumference	.170	.018	.734	9.299	.000
2	(Constant)	-11.336	1.293		-8.770	.000
	hip_circumference	.160	.012	.692	12.891	.000
	Sex Code (0=F,1=M)	-1.969	.210	-.504	-9.395	.000
3	(Constant)	-4.730	2.343		-2.019	.047
	hip_circumference	.171	.012	.741	14.106	.000
	Sex Code (0=F,1=M)	-1.196	.306	-.306	-3.909	.000
	Height (m)	-4.802	1.457	-.259	-3.296	.002
4	(Constant)	-2.689	2.382		-1.129	.263
	hip_circumference	.147	.015	.636	9.921	.000
	Sex Code (0=F,1=M)	-1.288	.296	-.330	-4.346	.000
	Height (m)	-5.039	1.403	-.272	-3.591	.001
	abdomen_SF	.031	.012	.167	2.630	.010

Coefficients[a]

Model		Collinearity Statistics Tolerance	Collinearity Statistics VIF
1	(Constant)		
	hip_circumference	1.000	1.000
2	(Constant)		
	hip_circumference	.993	1.007
	Sex Code (0=F,1=M)	.993	1.007
3	(Constant)		
	hip_circumference	.914	1.094
	Sex Code (0=F,1=M)	.410	2.438
	Height (m)	.407	2.458
4	(Constant)		
	hip_circumference	.566	1.767
	Sex Code (0=F,1=M)	.405	2.471
	Height (m)	.405	2.468
	abdomen_SF	.575	1.741

a. Dependent Variable: ICSoft tissue R (cm)

Excluded Variables[a]

Model		Beta In	t	Sig.	Partial Correlation	Collinearity .
						Tolerance
1	Age	.028[b]	.355	.724	.041	.979
	Sex Code (0=F,1=M)	-.504[b]	-9.395	.000	-.740	.993
	Height (m)	-.495[b]	-8.950	.000	-.723	.985
	Body Mass (kg)	-.620[b]	-6.948	.000	-.631	.477
	waist_circumference	-.252[b]	-2.173	.033	-.246	.441
	waist_breadth_M-L	-.356[b]	-3.563	.001	-.385	.539
	waist_breadth_A-P	-.052[b]	-.438	.663	-.051	.452
	suprailiac_SF	.151[b]	1.286	.202	.149	.449
	abdomen_SF	-.018[b]	-.176	.861	-.021	.616
	ant_pelvis_length	-.012[b]	-.149	.882	-.017	.957
	lat_pelvis_length_right	-.276[b]	-3.743	.000	-.401	.976
	AVG_lat_pelvis	-.282[b]	-3.847	.000	-.411	.981
	hip_breadth_M-L	-.098[b]	-.564	.574	-.066	.208
	pelvis_breadth_A-P	.025[b]	.218	.828	.026	.487
	right lat. thigh_length	-.331[b]	-4.733	.000	-.485	.991
	right med. thigh_length	-.407[b]	-6.251	.000	-.590	.969
	right mid. thigh_length	-.080[b]	-.993	.324	-.115	.955
	upper_thigh_circumference _R	.124[b]	.968	.336	.113	.381
	mid-thigh_circumference_R	.103[b]	1.077	.285	.125	.684
	upperthigh_A-P_breadth_R	-.045[b]	-.400	.690	-.047	.499
	max-thigh_M-L_breadth_R	.095[b]	1.003	.319	.117	.696
	max-thigh_A-P_breadth_R	-.122[b]	-1.128	.263	-.131	.530
	ant_mid-thigh_SF_R	.400[b]	5.241	.000	.523	.788
	post_mid-thigh_SF_R	.349[b]	4.585	.000	.473	.844
2	Age	.021[c]	.383	.703	.045	.978
	Height (m)	-.259[c]	-3.296	.002	-.362	.407
	Body Mass (kg)	-.035[c]	-.246	.807	-.029	.142
	waist_circumference	.195[c]	2.117	.038	.242	.321

Excluded Variables[a]

Model		Collinearity Statistics VIF	Minimum Tolerance
1	Age	1.022	.979
	Sex Code (0=F,1=M)	1.007	.993
	Height (m)	1.016	.985
	Body Mass (kg)	2.095	.477
	waist_circumference	2.267	.441
	waist_breadth_M-L	1.855	.539
	waist_breadth_A-P	2.213	.452
	suprailiac_SF	2.226	.449
	abdomen_SF	1.624	.616
	ant_pelvis_length	1.045	.957
	lat_pelvis_length_right	1.025	.976
	AVG_lat_pelvis	1.020	.981
	hip_breadth_M-L	4.797	.208
	pelvis_breadth_A-P	2.054	.487
	right lat. thigh_length	1.009	.991
	right med. thigh_length	1.032	.969
	right mid. thigh_length	1.047	.955
	upper_thigh_circumference_R	2.625	.381
	mid-thigh_circumference_R	1.461	.684
	upperthigh_A-P_breadth_R	2.006	.499
	max-thigh_M-L_breadth_R	1.436	.696
	max-thigh_A-P_breadth_R	1.888	.530
	ant_mid-thigh_SF_R	1.270	.788
	post_mid-thigh_SF_R	1.184	.844
2	Age	1.022	.972
	Height (m)	2.458	.407
	Body Mass (kg)	7.057	.142
	waist_circumference	3.112	.321

Excluded Variables[a]

Model		Beta In	t	Sig.	Partial Correlation	Collinearity . Tolerance
	waist_breadth_M-L	.117[c]	1.255	.214	.146	.329
	waist_breadth_A-P	.177[c]	2.188	.032	.250	.415
	suprailiac_SF	.145[c]	1.842	.070	.212	.449
	abdomen_SF	.153[c]	2.227	.029	.254	.577
	ant_pelvis_length	-.055[c]	-1.011	.315	-.118	.951
	lat_pelvis_length_right	-.073[c]	-1.220	.227	-.142	.799
	AVG_lat_pelvis	-.086[c]	-1.466	.147	-.170	.813
	hip_breadth_M-L	-.135[c]	-1.153	.253	-.135	.208
	pelvis_breadth_A-P	.098[c]	1.279	.205	.149	.482
	right lat. thigh_length	-.109[c]	-1.794	.077	-.207	.752
	right med. thigh_length	-.131[c]	-1.868	.066	-.215	.561
	right mid. thigh_length	-.060[c]	-1.089	.280	-.127	.953
	upper_thigh_circumference_R	.099[c]	1.145	.256	.134	.381
	mid-thigh_circumference_R	.095[c]	1.485	.142	.172	.684
	upperthigh_A-P_breadth_R	.062[c]	.805	.424	.094	.488
	max-thigh_M-L_breadth_R	.018[c]	.282	.779	.033	.685
	max-thigh_A-P_breadth_R	.052[c]	.682	.497	.080	.497
	ant_mid-thigh_SF_R	.113[c]	1.553	.125	.180	.529
	post_mid-thigh_SF_R	.082[c]	1.192	.237	.139	.605
3	Age	.013[d]	.245	.807	.029	.976
	Body Mass (kg)	.147[d]	1.026	.308	.121	.122
	waist_circumference	.146[d]	1.639	.106	.191	.310
	waist_breadth_M-L	.112[d]	1.287	.202	.151	.329
	waist_breadth_A-P	.146[d]	1.889	.063	.219	.408
	suprailiac_SF	.120[d]	1.613	.111	.188	.444
	abdomen_SF	.167[d]	2.630	.010	.298	.575
	ant_pelvis_length	-.038[d]	-.735	.465	-.087	.940
	lat_pelvis_length_right	-.051[d]	-.909	.367	-.107	.788

Excluded Variables[a]

Model		Collinearity Statistics VIF	Minimum Tolerance
	waist_breadth_M-L	3.039	.329
	waist_breadth_A-P	2.411	.415
	suprailiac_SF	2.226	.448
	abdomen_SF	1.733	.577
	ant_pelvis_length	1.052	.951
	lat_pelvis_length_right	1.251	.799
	AVG_lat_pelvis	1.231	.813
	hip_breadth_M-L	4.802	.208
	pelvis_breadth_A-P	2.074	.479
	right lat. thigh_length	1.330	.752
	right med. thigh_length	1.784	.561
	right mid. thigh_length	1.049	.947
	upper_thigh_circumference_R	2.627	.381
	mid-thigh_circumference_R	1.462	.682
	upperthigh_A-P_breadth_R	2.051	.485
	max-thigh_M-L_breadth_R	1.460	.685
	max-thigh_A-P_breadth_R	2.012	.497
	ant_mid-thigh_SF_R	1.889	.529
	post_mid-thigh_SF_R	1.652	.605
3	Age	1.025	.406
	Body Mass (kg)	8.170	.122
	waist_circumference	3.223	.310
	waist_breadth_M-L	3.040	.322
	waist_breadth_A-P	2.453	.377
	suprailiac_SF	2.250	.402
	abdomen_SF	1.741	.405
	ant_pelvis_length	1.064	.402
	lat_pelvis_length_right	1.269	.393

Excluded Variables[a]

Model		Beta In	t	Sig.	Partial Correlation	Collinearity . Tolerance
	AVG_lat_pelvis	-.059[d]	-1.056	.295	-.124	.793
	hip_breadth_M-L	-.181[d]	-1.654	.103	-.193	.205
	pelvis_breadth_A-P	.069[d]	.950	.345	.112	.474
	right lat. thigh_length	-.008[d]	-.110	.913	-.013	.530
	right med. thigh_length	-.047[d]	-.628	.532	-.074	.462
	right mid. thigh_length	-.055[d]	-1.065	.291	-.125	.952
	upper_thigh_circumference _R	.060[d]	.726	.470	.086	.372
	mid-thigh_circumference_R	.084[d]	1.389	.169	.163	.682
	upperthigh_A-P_breadth_R	.054[d]	.748	.457	.088	.487
	max-thigh_M-L_breadth_R	.016[d]	.267	.790	.032	.685
	max-thigh_A-P_breadth_R	.018[d]	.244	.808	.029	.486
	ant_mid-thigh_SF_R	.096[d]	1.400	.166	.164	.526
	post_mid-thigh_SF_R	.056[d]	.862	.391	.102	.596
4	Age	.023[e]	.465	.643	.056	.970
	Body Mass (kg)	.068[e]	.475	.636	.057	.116
	waist_circumference	.081[e]	.885	.379	.105	.278
	waist_breadth_M-L	.039[e]	.433	.667	.052	.288
	waist_breadth_A-P	.085[e]	1.050	.297	.125	.354
	suprailiac_SF	-.004[e]	-.038	.970	-.005	.261
	ant_pelvis_length	-.070[e]	-1.383	.171	-.163	.895
	lat_pelvis_length_right	-.087[e]	-1.583	.118	-.186	.749
	AVG_lat_pelvis	-.095[e]	-1.738	.087	-.203	.755
	hip_breadth_M-L	-.140[e]	-1.300	.198	-.154	.200
	pelvis_breadth_A-P	.080[e]	1.141	.258	.135	.473
	right lat. thigh_length	-.006[e]	-.095	.925	-.011	.530
	right med. thigh_length	-.032[e]	-.447	.656	-.053	.459
	right mid. thigh_length	-.044[e]	-.886	.378	-.105	.946
	upper_thigh_circumference _R	.059[e]	.746	.458	.089	.372

Excluded Variables[a]

Model		Collinearity Statistics VIF	Collinearity Statistics Minimum Tolerance
	AVG_lat_pelvis	1.261	.397
	hip_breadth_M-L	4.873	.197
	pelvis_breadth_A-P	2.108	.400
	right lat. thigh_length	1.886	.287
	right med. thigh_length	2.167	.335
	right mid. thigh_length	1.050	.406
	upper_thigh_circumference_R	2.688	.349
	mid-thigh_circumference_R	1.467	.405
	upperthigh_A-P_breadth_R	2.053	.404
	max-thigh_M-L_breadth_R	1.460	.407
	max-thigh_A-P_breadth_R	2.056	.380
	ant_mid-thigh_SF_R	1.900	.356
	post_mid-thigh_SF_R	1.678	.374
4	Age	1.031	.404
	Body Mass (kg)	8.622	.116
	waist_circumference	3.596	.278
	waist_breadth_M-L	3.469	.288
	waist_breadth_A-P	2.824	.354
	suprailiac_SF	3.831	.261
	ant_pelvis_length	1.117	.394
	lat_pelvis_length_right	1.334	.391
	AVG_lat_pelvis	1.324	.395
	hip_breadth_M-L	5.003	.156
	pelvis_breadth_A-P	2.114	.328
	right lat. thigh_length	1.886	.286
	right med. thigh_length	2.180	.332
	right mid. thigh_length	1.057	.405
	upper_thigh_circumference_R	2.688	.283

Excluded Variables[a]

Model		Beta In	t	Sig.	Partial Correlation	Collinearity . Tolerance
	mid-thigh_circumference_R	.076[e]	1.310	.194	.155	.680
	upperthigh_A-P_breadth_R	.086[e]	1.225	.225	.145	.474
	max-thigh_M-L_breadth_R	.043[e]	.726	.470	.086	.666
	max-thigh_A-P_breadth_R	.047[e]	.663	.510	.079	.475
	ant_mid-thigh_SF_R	.063[e]	.926	.358	.110	.504
	post_mid-thigh_SF_R	.043[e]	.687	.494	.082	.592

Excluded Variables[a]

Model		Collinearity Statistics	
		VIF	Minimum Tolerance
	mid-thigh_circumference_R	1.470	.404
	upperthigh_A-P_breadth_R	2.108	.315
	max-thigh_M-L_breadth_R	1.502	.403
	max-thigh_A-P_breadth_R	2.105	.314
	ant_mid-thigh_SF_R	1.983	.344
	post_mid-thigh_SF_R	1.689	.367

a. Dependent Variable: ICSoft tissue R (cm)

b. Predictors in the Model: (Constant), hip_circumference

c. Predictors in the Model: (Constant), hip_circumference, Sex Code (0=F,1=M)

d. Predictors in the Model: (Constant), hip_circumference, Sex Code (0=F,1=M), Height (m)

e. Predictors in the Model: (Constant), hip_circumference, Sex Code (0=F,1=M), Height (m), abdomen_SF

Coefficient Correlations[a]

Model			hip_circumference	Sex Code (0=F, 1=M)	Height (m)
1	Correlations	hip_circumference	1.000		
	Covariances	hip_circumference	.000		
2	Correlations	hip_circumference	1.000	.084	
		Sex Code (0=F,1=M)	.084	1.000	
	Covariances	hip_circumference	.000	.000	
		Sex Code (0=F,1=M)	.000	.044	
3	Correlations	hip_circumference	1.000	.268	-.282
		Sex Code (0=F,1=M)	.268	1.000	-.766
		Height (m)	-.282	-.766	1.000
	Covariances	hip_circumference	.000	.001	-.005
		Sex Code (0=F,1=M)	.001	.094	-.342
		Height (m)	-.005	-.342	2.122
4	Correlations	hip_circumference	1.000	.281	-.181
		Sex Code (0=F,1=M)	.281	1.000	-.752
		Height (m)	-.181	-.752	1.000
		abdomen_SF	-.617	-.117	-.064
	Covariances	hip_circumference	.000	.001	-.004
		Sex Code (0=F,1=M)	.001	.088	-.313
		Height (m)	-.004	-.313	1.969
		abdomen_SF	.000	.000	-.001

Coefficient Correlations[a]

Model			abdomen_SF
1	Correlations	hip_circumference	
	Covariances	hip_circumference	
2	Correlations	hip_circumference	
		Sex Code (0=F,1=M)	
	Covariances	hip_circumference	
		Sex Code (0=F,1=M)	
3	Correlations	hip_circumference	
		Sex Code (0=F,1=M)	
		Height (m)	
	Covariances	hip_circumference	
		Sex Code (0=F,1=M)	
		Height (m)	
4	Correlations	hip_circumference	-.617
		Sex Code (0=F,1=M)	-.117
		Height (m)	-.064
		abdomen_SF	1.000
	Covariances	hip_circumference	.000
		Sex Code (0=F,1=M)	.000
		Height (m)	-.001
		abdomen_SF	.000

a. Dependent Variable: ICSoft tissue R (cm)

Collinearity Diagnostics[a]

Model	Dimension	Eigenvalue	Condition Index	Variance Proportions (Constant)	hip_circumference	Sex Code (0=F, 1=M)
1	1	1.997	1.000	.00	.00	
	2	.003	24.379	1.00	1.00	
2	1	2.611	1.000	.00	.00	.05
	2	.386	2.602	.00	.00	.93
	3	.003	28.098	1.00	1.00	.02
3	1	3.587	1.000	.00	.00	.01
	2	.408	2.963	.00	.00	.41
	3	.004	29.926	.08	1.00	.07
	4	.001	63.547	.91	.00	.52
4	1	4.491	1.000	.00	.00	.01
	2	.422	3.260	.00	.00	.41
	3	.083	7.349	.00	.00	.00
	4	.003	40.841	.07	.98	.15
	5	.001	72.723	.93	.02	.43

Collinearity Diagnostics[a]

Model	Dimension	Variance Proportions Height (m)	abdomen_SF
1	1		
	2		
2	1		
	2		
	3		
3	1	.00	
	2	.00	
	3	.06	
	4	.94	
4	1	.00	.00
	2	.00	.01
	3	.00	.62
	4	.10	.32
	5	.89	.05

a. Dependent Variable: ICSoft tissue R (cm)

Residuals Statistics[a]

	Minimum	Maximum	Mean	Std. Deviation	N
Predicted Value	.2523	8.1826	4.1197	1.79607	76
Std. Predicted Value	-2.153	2.262	.000	1.000	76
Standard Error of Predicted Value	.134	.394	.204	.052	76
Adjusted Predicted Value	.0264	8.1229	4.1108	1.79397	76
Residual	-1.68408	2.15020	.00000	.79921	76
Std. Residual	-2.050	2.618	.000	.973	76
Stud. Residual	-2.111	2.983	.005	1.013	76
Deleted Residual	-1.79697	2.79266	.00891	.86899	76
Stud. Deleted Residual	-2.165	3.167	.008	1.030	76
Mahal. Distance	.998	16.267	3.947	2.794	76
Cook's Distance	.000	.532	.018	.062	76
Centered Leverage Value	.013	.217	.053	.037	76

a. Dependent Variable: ICSoft tissue R (cm)

Charts

Histogram

Dependent Variable: ICSoft tissue R (cm)

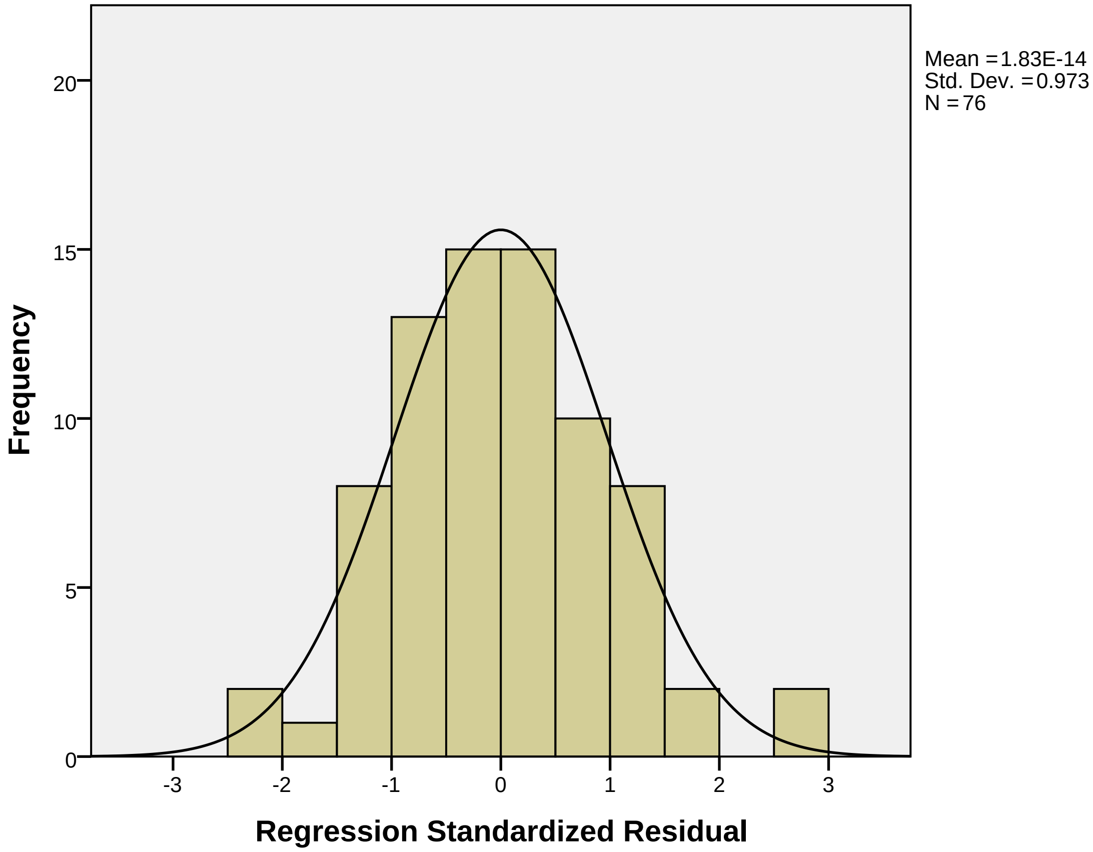

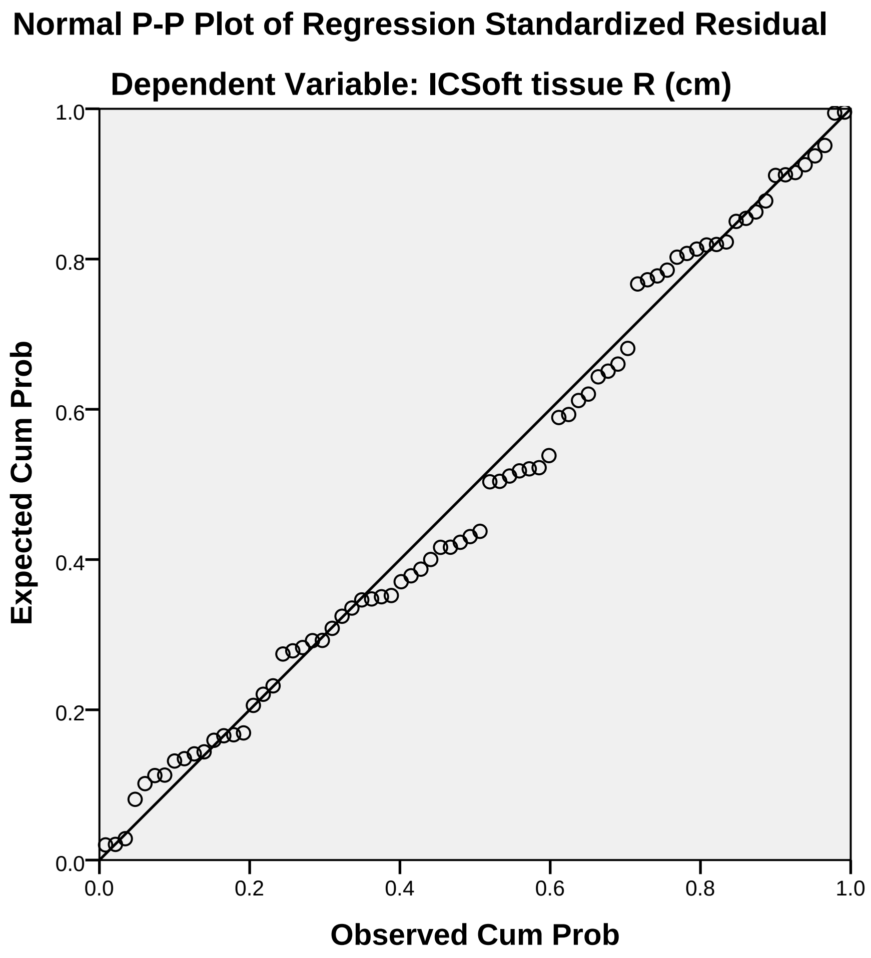
Normal P-P Plot of Regression Standardized Residual
Dependent Variable: ICSoft tissue R (cm)
Expected Cum Prob
Observed Cum Prob
0.0
0.2
0.4
0.6
0.8
1.0

Scatterplot

Dependent Variable: ICSoft tissue R (cm)

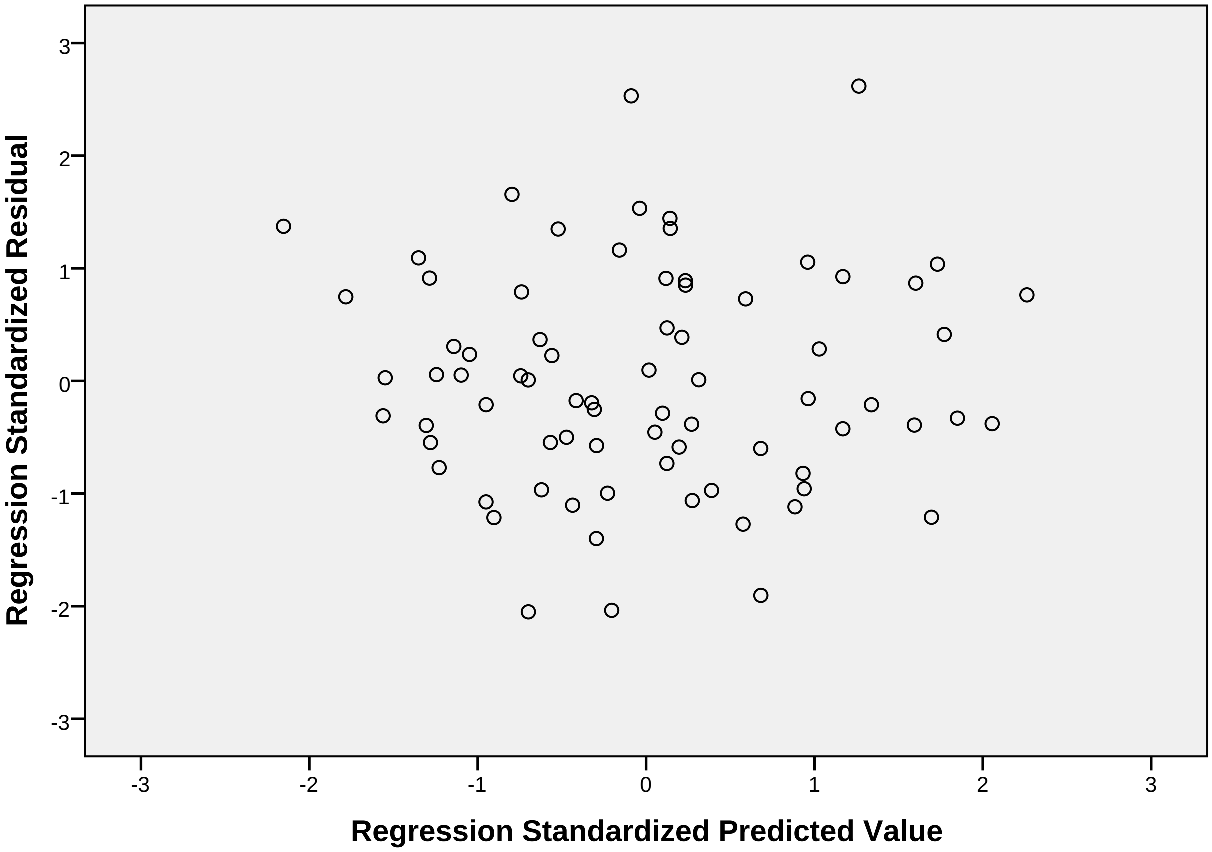

Regression

Notes

Output Created		21-NOV-2017 12:26:41
Comments		
Input	Data	F:\Claudia Regression\Danielle FIX\Generation-Validation Groups\SPSS Data_REGRESSION_G groups final_OLDER_no 0fm488+0mt203.sav
	Active Dataset	DataSet3
	Filter	<none>
	Weight	<none>
	Split File	<none>
	N of Rows in Working Data File	76
Missing Value Handling	Definition of Missing	User-defined missing values are treated as missing.
	Cases Used	Statistics are based on cases with no missing values for any variable used.

Notes

Syntax		REGRESSION<br>/MISSING LISTWISE<br>/STATISTICS COEFF OUTS BCOV R ANOVA COLLIN TOL<br>/CRITERIA=PIN(.05) POUT(.10)<br>/NOORIGIN<br>/DEPENDENT IC_SofttissueLcm<br>/METHOD=STEPWISE Age SexCode0F1M BodyMasskg BMI waist_circumference waist_breadth_ML<br>waist_breadth_AP suprailiac_SF abdomen_SF ant_pelvis_length lat_pelvis_length_left AVG_lat_pelvis<br>hip_circumference hip_breadth_ML pelvis_breadth_AP leftlat.thigh_length leftmed.thigh_length<br>leftmid.thigh_length upper_thigh_circumference_L midthigh_circumference_L upper_thigh_AP_breadth_L<br><br>maxthigh_ML_breadth_L maxthigh_AP_breadth_L ant_midthigh_SF_L post_midthigh_SF_L Heightm<br>/SCATTERPLOT=(*ZRESID ,*ZPRED)<br>/RESIDUALS HISTOGRAM(ZRESID) NORMPROB(ZRESID)<br>/CASEWISE PLOT(ZRESID) OUTLIERS(3)<br>/SAVE ZPRED MAHAL COOK ZRESID.
Resources	Processor Time	00:00:00.37
	Elapsed Time	00:00:00.29

Notes

	Memory Required	20220 bytes
	Additional Memory Required for Residual Plots	712 bytes
Variables Created or Modified	ZPR_3	Standardized Predicted Value
	ZRE_3	Standardized Residual
	MAH_3	Mahalanobis Distance
	COO_3	Cook's Distance

Variables Entered/Removed[a]

Model	Variables Entered	Variables Removed	Method
1	hip_circumference	.	Stepwise (Criteria: Probability-of-F-to-enter <= .050, Probability-of-F-to-remove >= .100).
2	Sex Code (0=F,1=M)	.	Stepwise (Criteria: Probability-of-F-to-enter <= .050, Probability-of-F-to-remove >= .100).
3	Height (m)	.	Stepwise (Criteria: Probability-of-F-to-enter <= .050, Probability-of-F-to-remove >= .100).
4	abdomen_SF	.	Stepwise (Criteria: Probability-of-F-to-enter <= .050, Probability-of-F-to-remove >= .100).

a. Dependent Variable: IC_Soft tissue L (cm)

Model Summary[e]

Model	R	R Square	Adjusted R Square	Std. Error of the Estimate
1	.710[a]	.504	.497	1.31994
2	.899[b]	.809	.804	.82466
3	.918[c]	.844	.837	.75115
4	.927[d]	.860	.852	.71546

a. Predictors: (Constant), hip_circumference

b. Predictors: (Constant), hip_circumference, Sex Code (0=F,1=M)

c. Predictors: (Constant), hip_circumference, Sex Code (0=F,1=M), Height (m)

d. Predictors: (Constant), hip_circumference, Sex Code (0=F,1=M), Height (m), abdomen_SF

e. Dependent Variable: IC_Soft tissue L (cm)

ANOVA[a]

Model		Sum of Squares	df	Mean Square	F	Sig.
1	Regression	130.783	1	130.783	75.066	.000[b]
	Residual	128.926	74	1.742		
	Total	259.709	75			
2	Regression	210.064	2	105.032	154.443	.000[c]
	Residual	49.645	73	.680		
	Total	259.709	75			
3	Regression	219.085	3	73.028	129.432	.000[d]
	Residual	40.624	72	.564		
	Total	259.709	75			
4	Regression	223.366	4	55.841	109.092	.000[e]
	Residual	36.343	71	.512		
	Total	259.709	75			

a. Dependent Variable: IC_Soft tissue L (cm)

b. Predictors: (Constant), hip_circumference

c. Predictors: (Constant), hip_circumference, Sex Code (0=F,1=M)

d. Predictors: (Constant), hip_circumference, Sex Code (0=F,1=M), Height (m)

e. Predictors: (Constant), hip_circumference, Sex Code (0=F,1=M), Height (m), abdomen_SF

Coefficients[a]

Model		Unstandardized Coefficients B	Std. Error	Standardized Coefficients Beta	t	Sig.
1	(Constant)	-12.170	1.849		-6.583	.000
	hip_circumference	.155	.018	.710	8.664	.000
2	(Constant)	-10.099	1.171		-8.626	.000
	hip_circumference	.145	.011	.663	12.913	.000
	Sex Code (0=F,1=M)	-2.050	.190	-.554	-10.797	.000
3	(Constant)	-3.054	2.060		-1.483	.142
	hip_circumference	.157	.011	.718	14.729	.000
	Sex Code (0=F,1=M)	-1.226	.269	-.332	-4.556	.000
	Height (m)	-5.121	1.281	-.292	-3.999	.000
4	(Constant)	-1.100	2.075		-.530	.598
	hip_circumference	.134	.013	.613	10.381	.000
	Sex Code (0=F,1=M)	-1.313	.258	-.355	-5.089	.000
	Height (m)	-5.348	1.222	-.305	-4.375	.000
	abdomen_SF	.030	.010	.169	2.892	.005

Coefficients[a]

Model		Collinearity Statistics Tolerance	VIF
1	(Constant)		
	hip_circumference	1.000	1.000
2	(Constant)		
	hip_circumference	.993	1.007
	Sex Code (0=F,1=M)	.993	1.007
3	(Constant)		
	hip_circumference	.914	1.094
	Sex Code (0=F,1=M)	.410	2.438
	Height (m)	.407	2.458
4	(Constant)		
	hip_circumference	.566	1.767
	Sex Code (0=F,1=M)	.405	2.471
	Height (m)	.405	2.468
	abdomen_SF	.575	1.741

a. Dependent Variable: IC_Soft tissue L (cm)

Excluded Variables[a]

Model		Beta In	t	Sig.	Partial Correlation	Collinearity . Tolerance
1	Age	.018[b]	.217	.829	.025	.979
	Sex Code (0=F,1=M)	-.554[b]	-10.797	.000	-.784	.993
	Body Mass (kg)	-.702[b]	-8.107	.000	-.688	.477
	BMI	-.128[b]	-.839	.404	-.098	.290
	waist_circumference	-.287[b]	-2.405	.019	-.271	.441
	waist_breadth_M-L	-.373[b]	-3.610	.001	-.389	.539
	waist_breadth_A-P	-.051[b]	-.419	.677	-.049	.452
	suprailiac_SF	.162[b]	1.332	.187	.154	.449
	abdomen_SF	-.034[b]	-.320	.750	-.037	.616
	ant_pelvis_length	.035[b]	.413	.681	.048	.957
	lat_pelvis_length_left	-.280[b]	-3.675	.000	-.395	.986
	AVG_lat_pelvis	-.280[b]	-3.658	.000	-.394	.981
	hip_breadth_M-L	.052[b]	.285	.776	.033	.208
	pelvis_breadth_A-P	-.032[b]	-.267	.790	-.031	.487
	left lat. thigh_length	-.394[b]	-5.764	.000	-.559	.998
	left med. thigh_length	-.411[b]	-6.040	.000	-.577	.980
	left mid. thigh_length	-.079[b]	-.933	.354	-.109	.943
	upper_thigh_circumference_L	.100[b]	.813	.419	.095	.445
	mid-thigh_circumference_L	.028[b]	.261	.795	.030	.577
	upper_thigh_A-P_breadth_L	-.003[b]	-.026	.979	-.003	.514
	max-thigh_M-L_breadth_L	.231[b]	2.449	.017	.276	.704
	max-thigh_A-P_breadth_L	-.042[b]	-.409	.684	-.048	.642
	ant_mid-thigh_SF_L	.435[b]	5.577	.000	.547	.784
	post_mid-thigh_SF_L	.384[b]	5.042	.000	.508	.868
	Height (m)	-.547[b]	-10.336	.000	-.771	.985
2	Age	.010[c]	.187	.852	.022	.978
	Body Mass (kg)	-.106[c]	-.776	.440	-.091	.142
	BMI	.275[c]	2.831	.006	.316	.252

Excluded Variables[a]

Model		Collinearity Statistics VIF	Minimum Tolerance
1	Age	1.022	.979
	Sex Code (0=F,1=M)	1.007	.993
	Body Mass (kg)	2.095	.477
	BMI	3.454	.290
	waist_circumference	2.267	.441
	waist_breadth_M-L	1.855	.539
	waist_breadth_A-P	2.213	.452
	suprailiac_SF	2.226	.449
	abdomen_SF	1.624	.616
	ant_pelvis_length	1.045	.957
	lat_pelvis_length_left	1.015	.986
	AVG_lat_pelvis	1.020	.981
	hip_breadth_M-L	4.797	.208
	pelvis_breadth_A-P	2.054	.487
	left lat. thigh_length	1.002	.998
	left med. thigh_length	1.021	.980
	left mid. thigh_length	1.061	.943
	upper_thigh_circumference_L	2.249	.445
	mid-thigh_circumference_L	1.732	.577
	upper_thigh_A-P_breadth_L	1.945	.514
	max-thigh_M-L_breadth_L	1.420	.704
	max-thigh_A-P_breadth_L	1.558	.642
	ant_mid-thigh_SF_L	1.276	.784
	post_mid-thigh_SF_L	1.152	.868
	Height (m)	1.016	.985
2	Age	1.022	.972
	Body Mass (kg)	7.057	.142
	BMI	3.963	.252

Excluded Variables[a]

Model		Beta In	t	Sig.	Partial Correlation	Collinearity . Tolerance
	waist_circumference	.200[c]	2.284	.025	.260	.321
	waist_breadth_M-L	.158[c]	1.794	.077	.207	.329
	waist_breadth_A-P	.201[c]	2.629	.010	.296	.415
	suprailiac_SF	.155[c]	2.077	.041	.238	.449
	abdomen_SF	.153[c]	2.338	.022	.266	.577
	ant_pelvis_length	-.013[c]	-.237	.813	-.028	.951
	lat_pelvis_length_left	-.072[c]	-1.285	.203	-.150	.832
	AVG_lat_pelvis	-.059[c]	-1.043	.300	-.122	.813
	hip_breadth_M-L	.011[c]	.099	.921	.012	.208
	pelvis_breadth_A-P	.048[c]	.653	.516	.077	.482
	left lat. thigh_length	-.151[c]	-2.634	.010	-.296	.737
	left med. thigh_length	-.128[c]	-2.054	.044	-.235	.644
	left mid. thigh_length	-.063[c]	-1.191	.238	-.139	.942
	upper_thigh_circumference_L	.087[c]	1.136	.260	.133	.444
	mid-thigh_circumference_L	.075[c]	1.110	.271	.130	.575
	upper_thigh_A-P_breadth_L	.069[c]	.961	.340	.113	.510
	max-thigh_M-L_breadth_L	.067[c]	1.065	.290	.125	.658
	max-thigh_A-P_breadth_L	.081[c]	1.260	.212	.147	.622
	ant_mid-thigh_SF_L	.084[c]	1.155	.252	.135	.489
	post_mid-thigh_SF_L	.070[c]	1.049	.298	.123	.588
	Height (m)	-.292[c]	-3.999	.000	-.426	.407
3	Age	.000[d]	.006	.995	.001	.976
	Body Mass (kg)	.089[d]	.666	.507	.079	.122
	BMI	.094[d]	.832	.408	.098	.170
	waist_circumference	.144[d]	1.751	.084	.203	.310
	waist_breadth_M-L	.153[d]	1.914	.060	.221	.329
	waist_breadth_A-P	.166[d]	2.341	.022	.268	.408
	suprailiac_SF	.127[d]	1.854	.068	.215	.444

Excluded Variables[a]

Model		Collinearity Statistics VIF	Collinearity Statistics Minimum Tolerance
	waist_circumference	3.112	.321
	waist_breadth_M-L	3.039	.329
	waist_breadth_A-P	2.411	.415
	suprailiac_SF	2.226	.448
	abdomen_SF	1.733	.577
	ant_pelvis_length	1.052	.951
	lat_pelvis_length_left	1.202	.832
	AVG_lat_pelvis	1.231	.813
	hip_breadth_M-L	4.802	.208
	pelvis_breadth_A-P	2.074	.479
	left lat. thigh_length	1.357	.733
	left med. thigh_length	1.552	.644
	left mid. thigh_length	1.061	.936
	upper_thigh_circumference_L	2.250	.444
	mid-thigh_circumference_L	1.739	.571
	upper_thigh_A-P_breadth_L	1.962	.506
	max-thigh_M-L_breadth_L	1.520	.658
	max-thigh_A-P_breadth_L	1.607	.622
	ant_mid-thigh_SF_L	2.043	.489
	post_mid-thigh_SF_L	1.701	.588
	Height (m)	2.458	.407
3	Age	1.025	.406
	Body Mass (kg)	8.170	.122
	BMI	5.883	.158
	waist_circumference	3.223	.310
	waist_breadth_M-L	3.040	.322
	waist_breadth_A-P	2.453	.377
	suprailiac_SF	2.250	.402

Excluded Variables[a]

Model		Beta In	t	Sig.	Partial Correlation	Collinearity . Tolerance
	abdomen_SF	.169[d]	2.892	.005	.325	.575
	ant_pelvis_length	.008[d]	.155	.877	.018	.940
	lat_pelvis_length_left	-.035[d]	-.676	.501	-.080	.803
	AVG_lat_pelvis	-.028[d]	-.533	.596	-.063	.793
	hip_breadth_M-L	-.039[d]	-.373	.711	-.044	.205
	pelvis_breadth_A-P	.015[d]	.219	.827	.026	.474
	left lat. thigh_length	-.045[d]	-.690	.492	-.082	.513
	left med. thigh_length	-.046[d]	-.722	.473	-.085	.549
	left mid. thigh_length	-.054[d]	-1.119	.267	-.132	.940
	upper_thigh_circumference_L	.042[d]	.597	.553	.071	.433
	mid-thigh_circumference_L	.059[d]	.958	.341	.113	.573
	upper_thigh_A-P_breadth_L	.068[d]	1.037	.303	.122	.510
	max-thigh_M-L_breadth_L	.066[d]	1.146	.256	.135	.658
	max-thigh_A-P_breadth_L	.051[d]	.857	.394	.101	.611
	ant_mid-thigh_SF_L	.061[d]	.918	.362	.108	.486
	post_mid-thigh_SF_L	.048[d]	.787	.434	.093	.583
4	Age	.011[e]	.237	.813	.028	.970
	Body Mass (kg)	.005[e]	.041	.967	.005	.116
	BMI	.052[e]	.471	.639	.056	.167
	waist_circumference	.078[e]	.930	.356	.110	.278
	waist_breadth_M-L	.084[e]	1.021	.311	.121	.288
	waist_breadth_A-P	.107[e]	1.445	.153	.170	.354
	suprailiac_SF	.006[e]	.073	.942	.009	.261
	ant_pelvis_length	-.022[e]	-.476	.635	-.057	.895
	lat_pelvis_length_left	-.069[e]	-1.363	.177	-.161	.768
	AVG_lat_pelvis	-.063[e]	-1.228	.223	-.145	.755
	hip_breadth_M-L	.007[e]	.072	.943	.009	.200
	pelvis_breadth_A-P	.025[e]	.392	.696	.047	.473

Excluded Variables[a]

Model		Collinearity Statistics VIF	Minimum Tolerance
	abdomen_SF	1.741	.405
	ant_pelvis_length	1.064	.402
	lat_pelvis_length_left	1.245	.393
	AVG_lat_pelvis	1.261	.397
	hip_breadth_M-L	4.873	.197
	pelvis_breadth_A-P	2.108	.400
	left lat. thigh_length	1.949	.283
	left med. thigh_length	1.822	.347
	left mid. thigh_length	1.064	.406
	upper_thigh_circumference_L	2.312	.396
	mid-thigh_circumference_L	1.747	.405
	upper_thigh_A-P_breadth_L	1.962	.407
	max-thigh_M-L_breadth_L	1.520	.399
	max-thigh_A-P_breadth_L	1.636	.392
	ant_mid-thigh_SF_L	2.059	.345
	post_mid-thigh_SF_L	1.715	.360
4	Age	1.031	.404
	Body Mass (kg)	8.622	.116
	BMI	6.002	.152
	waist_circumference	3.596	.278
	waist_breadth_M-L	3.469	.288
	waist_breadth_A-P	2.824	.354
	suprailiac_SF	3.831	.261
	ant_pelvis_length	1.117	.394
	lat_pelvis_length_left	1.303	.393
	AVG_lat_pelvis	1.324	.395
	hip_breadth_M-L	5.003	.156
	pelvis_breadth_A-P	2.114	.328

Excluded Variables[a]

Model		Beta In	t	Sig.	Partial Correlation	Collinearity .
						Tolerance
	left lat. thigh_length	-.034[e]	-.539	.592	-.064	.511
	left med. thigh_length	-.026[e]	-.424	.673	-.051	.542
	left mid. thigh_length	-.040[e]	-.859	.393	-.102	.929
	upper_thigh_circumference _L	.044[e]	.656	.514	.078	.433
	mid-thigh_circumference_L	.064[e]	1.095	.277	.130	.572
	upper_thigh_A-P_breadth_L	.076[e]	1.221	.226	.144	.509
	max-thigh_M-L_breadth_L	.075[e]	1.378	.173	.163	.656
	max-thigh_A-P_breadth_L	.068[e]	1.195	.236	.141	.606
	ant_mid-thigh_SF_L	.022[e]	.340	.735	.041	.463
	post_mid-thigh_SF_L	.025[e]	.426	.671	.051	.572

Excluded Variables[a]

Model		Collinearity Statistics	
		VIF	Minimum Tolerance
	left lat. thigh_length	1.957	.281
	left med. thigh_length	1.847	.343
	left mid. thigh_length	1.077	.404
	upper_thigh_circumference _L	2.312	.314
	mid-thigh_circumference_L	1.748	.386
	upper_thigh_A-P_breadth_L	1.966	.355
	max-thigh_M-L_breadth_L	1.525	.395
	max-thigh_A-P_breadth_L	1.651	.385
	ant_mid-thigh_SF_L	2.162	.332
	post_mid-thigh_SF_L	1.749	.350

a. Dependent Variable: IC_Soft tissue L (cm)

b. Predictors in the Model: (Constant), hip_circumference

c. Predictors in the Model: (Constant), hip_circumference, Sex Code (0=F,1=M)

d. Predictors in the Model: (Constant), hip_circumference, Sex Code (0=F,1=M), Height (m)

e. Predictors in the Model: (Constant), hip_circumference, Sex Code (0=F,1=M), Height (m), abdomen_SF

Coefficient Correlations[a]

Model			hip_circumference	Sex Code (0=F, 1=M)	Height (m)
1	Correlations	hip_circumference	1.000		
	Covariances	hip_circumference	.000		
2	Correlations	hip_circumference	1.000	.084	
		Sex Code (0=F,1=M)	.084	1.000	
	Covariances	hip_circumference	.000	.000	
		Sex Code (0=F,1=M)	.000	.036	
3	Correlations	hip_circumference	1.000	.268	-.282
		Sex Code (0=F,1=M)	.268	1.000	-.766
		Height (m)	-.282	-.766	1.000
	Covariances	hip_circumference	.000	.001	-.004
		Sex Code (0=F,1=M)	.001	.072	-.264
		Height (m)	-.004	-.264	1.640
4	Correlations	hip_circumference	1.000	.281	-.181
		Sex Code (0=F,1=M)	.281	1.000	-.752
		Height (m)	-.181	-.752	1.000
		abdomen_SF	-.617	-.117	-.064
	Covariances	hip_circumference	.000	.001	-.003
		Sex Code (0=F,1=M)	.001	.067	-.237
		Height (m)	-.003	-.237	1.494
		abdomen_SF	-8.284E-5	.000	-.001

Coefficient Correlations[a]

Model			abdomen_SF
1	Correlations	hip_circumference	
	Covariances	hip_circumference	
2	Correlations	hip_circumference	
		Sex Code (0=F,1=M)	
	Covariances	hip_circumference	
		Sex Code (0=F,1=M)	
3	Correlations	hip_circumference	
		Sex Code (0=F,1=M)	
		Height (m)	
	Covariances	hip_circumference	
		Sex Code (0=F,1=M)	
		Height (m)	
4	Correlations	hip_circumference	-.617
		Sex Code (0=F,1=M)	-.117
		Height (m)	-.064
		abdomen_SF	1.000
	Covariances	hip_circumference	-8.284E-5
		Sex Code (0=F,1=M)	.000
		Height (m)	-.001
		abdomen_SF	.000

a. Dependent Variable: IC_Soft tissue L (cm)

Collinearity Diagnostics[a]

Model	Dimension	Eigenvalue	Condition Index	Variance Proportions (Constant)	hip_circumference	Sex Code (0=F, 1=M)
1	1	1.997	1.000	.00	.00	
	2	.003	24.379	1.00	1.00	
2	1	2.611	1.000	.00	.00	.05
	2	.386	2.602	.00	.00	.93
	3	.003	28.098	1.00	1.00	.02
3	1	3.587	1.000	.00	.00	.01
	2	.408	2.963	.00	.00	.41
	3	.004	29.926	.08	1.00	.07
	4	.001	63.547	.91	.00	.52
4	1	4.491	1.000	.00	.00	.01
	2	.422	3.260	.00	.00	.41
	3	.083	7.349	.00	.00	.00
	4	.003	40.841	.07	.98	.15
	5	.001	72.723	.93	.02	.43

Collinearity Diagnostics[a]

Model	Dimension	Variance Proportions Height (m)	abdomen_SF
1	1		
	2		
2	1		
	2		
	3		
3	1	.00	
	2	.00	
	3	.06	
	4	.94	
4	1	.00	.00
	2	.00	.01
	3	.00	.62
	4	.10	.32
	5	.89	.05

a. Dependent Variable: IC_Soft tissue L (cm)

Residuals Statistics[a]

	Minimum	Maximum	Mean	Std. Deviation	N
Predicted Value	.0000	7.6409	3.7930	1.72575	76
Std. Predicted Value	-2.198	2.230	.000	1.000	76
Standard Error of Predicted Value	.116	.343	.178	.046	76
Adjusted Predicted Value	-.2083	7.5687	3.7834	1.72391	76
Residual	-1.40243	1.86556	.00000	.69612	76
Std. Residual	-1.960	2.608	.000	.973	76
Stud. Residual	-2.032	2.669	.006	1.011	76
Deleted Residual	-1.50638	2.17136	.00959	.75250	76
Stud. Deleted Residual	-2.078	2.794	.009	1.027	76
Mahal. Distance	.998	16.267	3.947	2.794	76
Cook's Distance	.000	.424	.017	.051	76
Centered Leverage Value	.013	.217	.053	.037	76

a. Dependent Variable: IC_Soft tissue L (cm)

Regression

Notes

Output Created		24-NOV-2017 13:16:03
Comments		
Input	Data	C:\Users\User\Desktop\Danielle FIX\Generation-Validation Groups\SPSS Data_REGRESSION_G groups final_OLDER_no 0fm488+0mt203.sav
	Active Dataset	DataSet2
	Filter	<none>
	Weight	<none>
	Split File	<none>
	N of Rows in Working Data File	76
Missing Value Handling	Definition of Missing	User-defined missing values are treated as missing.
	Cases Used	Statistics are based on cases with no missing values for any variable used.
Syntax		REGRESSION /MISSING LISTWISE /STATISTICS COEFF OUTS BCOV R ANOVA COLLIN TOL /CRITERIA=PIN(.05) POUT(.10) /NOORIGIN /DEPENDENT GT_SoftTissueRcm /METHOD=STEPWISE SexCode0F1M ant_midthigh_SF_R hip_circumference Heightm maxthigh_ML_breadth_R /SCATTERPLOT=(*ZRESID ,*ZPRED) /RESIDUALS HISTOGRAM(ZRESID) NORMPROB(ZRESID) /CASEWISE PLOT(ZRESID) OUTLIERS(3) /SAVE ZPRED MAHAL COOK ZRESID.

Notes

Resources	Processor Time	00:00:00.41
	Elapsed Time	00:00:00.31
	Memory Required	4644 bytes
	Additional Memory Required for Residual Plots	880 bytes
Variables Created or Modified	ZPR_11	Standardized Predicted Value
	ZRE_11	Standardized Residual
	MAH_11	Mahalanobis Distance
	COO_11	Cook's Distance

Variables Entered/Removed[a]

Model	Variables Entered	Variables Removed	Method
1	ant_mid-thigh_SF_R	.	Stepwise (Criteria: Probability-of-F-to-enter <= .050, Probability-of-F-to-remove >= .100).
2	max-thigh_M-L_breadth_R	.	Stepwise (Criteria: Probability-of-F-to-enter <= .050, Probability-of-F-to-remove >= .100).

Variables Entered/Removed[a]

Model	Variables Entered	Variables Removed	Method
3	Sex Code (0=F,1=M)	.	Stepwise (Criteria: Probability-of-F-to-enter <= .050, Probability-of-F-to-remove >= .100).
4	hip_circumference	.	Stepwise (Criteria: Probability-of-F-to-enter <= .050, Probability-of-F-to-remove >= .100).
5	Height (m)	.	Stepwise (Criteria: Probability-of-F-to-enter <= .050, Probability-of-F-to-remove >= .100).

a. Dependent Variable: GT_Soft Tissue R (cm)

Model Summary[f]

Model	R	R Square	Adjusted R Square	Std. Error of the Estimate
1	.765[a]	.585	.580	1.36223
2	.830[b]	.688	.680	1.18938
3	.884[c]	.782	.773	1.00076
4	.911[d]	.829	.820	.89244
5	.919[e]	.844	.833	.85889

a. Predictors: (Constant), ant_mid-thigh_SF_R

b. Predictors: (Constant), ant_mid-thigh_SF_R, max-thigh_M-L_breadth_R

c. Predictors: (Constant), ant_mid-thigh_SF_R, max-thigh_M-L_breadth_R, Sex Code (0=F,1=M)

d. Predictors: (Constant), ant_mid-thigh_SF_R, max-thigh_M-L_breadth_R, Sex Code (0=F,1=M), hip_circumference

e. Predictors: (Constant), ant_mid-thigh_SF_R, max-thigh_M-L_breadth_R, Sex Code (0=F,1=M), hip_circumference, Height (m)

f. Dependent Variable: GT_Soft Tissue R (cm)

ANOVA[a]

Model		Sum of Squares	df	Mean Square	F	Sig.
1	Regression	193.748	1	193.748	104.409	.000[b]
	Residual	137.319	74	1.856		
	Total	331.067	75			
2	Regression	227.799	2	113.900	80.515	.000[c]
	Residual	103.268	73	1.415		
	Total	331.067	75			
3	Regression	258.958	3	86.319	86.188	.000[d]
	Residual	72.110	72	1.002		
	Total	331.067	75			
4	Regression	274.519	4	68.630	86.170	.000[e]
	Residual	56.548	71	.796		
	Total	331.067	75			
5	Regression	279.428	5	55.886	75.757	.000[f]
	Residual	51.639	70	.738		
	Total	331.067	75			

a. Dependent Variable: GT_Soft Tissue R (cm)

b. Predictors: (Constant), ant_mid-thigh_SF_R

c. Predictors: (Constant), ant_mid-thigh_SF_R, max-thigh_M-L_breadth_R

d. Predictors: (Constant), ant_mid-thigh_SF_R, max-thigh_M-L_breadth_R, Sex Code (0=F,1=M)

e. Predictors: (Constant), ant_mid-thigh_SF_R, max-thigh_M-L_breadth_R, Sex Code (0=F,1=M), hip_circumference

f. Predictors: (Constant), ant_mid-thigh_SF_R, max-thigh_M-L_breadth_R, Sex Code (0=F,1=M), hip_circumference, Height (m)

Coefficients[a]

Model		Unstandardized Coefficients B	Unstandardized Coefficients Std. Error	Standardized Coefficients Beta	t	Sig.
1	(Constant)	1.620	.340		4.769	.000
	ant_mid-thigh_SF_R	.125	.012	.765	10.218	.000
2	(Constant)	-4.434	1.269		-3.494	.001
	ant_mid-thigh_SF_R	.108	.011	.660	9.603	.000
	max-thigh_M-L_breadth_R	.379	.077	.337	4.906	.000
3	(Constant)	-2.988	1.099		-2.719	.008
	ant_mid-thigh_SF_R	.075	.011	.459	6.726	.000
	max-thigh_M-L_breadth_R	.387	.065	.344	5.946	.000
	Sex Code (0=F,1=M)	-1.528	.274	-.366	-5.578	.000
4	(Constant)	-7.174	1.363		-5.264	.000
	ant_mid-thigh_SF_R	.054	.011	.330	4.902	.000
	max-thigh_M-L_breadth_R	.242	.067	.215	3.632	.001
	Sex Code (0=F,1=M)	-1.801	.252	-.431	-7.148	.000
	hip_circumference	.071	.016	.288	4.420	.000
5	(Constant)	-2.025	2.389		-.848	.399
	ant_mid-thigh_SF_R	.052	.011	.318	4.880	.000
	max-thigh_M-L_breadth_R	.241	.064	.214	3.749	.000
	Sex Code (0=F,1=M)	-1.219	.331	-.292	-3.682	.000
	hip_circumference	.082	.016	.330	5.095	.000
	Height (m)	-3.789	1.469	-.191	-2.580	.012

Coefficients[a]

Model		Collinearity Statistics Tolerance	VIF
1	(Constant)		
	ant_mid-thigh_SF_R	1.000	1.000
2	(Constant)		
	ant_mid-thigh_SF_R	.904	1.107
	max-thigh_M-L_breadth_R	.904	1.107
3	(Constant)		
	ant_mid-thigh_SF_R	.650	1.538
	max-thigh_M-L_breadth_R	.903	1.107
	Sex Code (0=F,1=M)	.703	1.423
4	(Constant)		
	ant_mid-thigh_SF_R	.529	1.889
	max-thigh_M-L_breadth_R	.685	1.460
	Sex Code (0=F,1=M)	.660	1.514
	hip_circumference	.567	1.764
5	(Constant)		
	ant_mid-thigh_SF_R	.526	1.900
	max-thigh_M-L_breadth_R	.685	1.460
	Sex Code (0=F,1=M)	.354	2.824
	hip_circumference	.531	1.883
	Height (m)	.404	2.473

a. Dependent Variable: GT_Soft Tissue R (cm)

Excluded Variables[a]

Model		Beta In	t	Sig.	Partial Correlation	Collinearity . Tolerance
1	Sex Code (0=F,1=M)	-.358[b]	-4.498	.000	-.466	.703
	hip_circumference	.308[b]	4.002	.000	.424	.788
	Height (m)	-.242[b]	-3.184	.002	-.349	.867
	max-thigh_M-L_breadth_R	.337[b]	4.906	.000	.498	.904
2	Sex Code (0=F,1=M)	-.366[c]	-5.578	.000	-.549	.703
	hip_circumference	.174[c]	2.112	.038	.242	.603
	Height (m)	-.282[c]	-4.483	.000	-.467	.857
3	hip_circumference	.288[d]	4.420	.000	.465	.567
	Height (m)	-.096[d]	-1.151	.254	-.135	.432
4	Height (m)	-.191[e]	-2.580	.012	-.295	.404

Excluded Variables[a]

Model		Collinearity Statistics VIF	Minimum Tolerance
1	Sex Code (0=F,1=M)	1.422	.703
	hip_circumference	1.270	.788
	Height (m)	1.153	.867
	max-thigh_M-L_breadth_R	1.107	.904
2	Sex Code (0=F,1=M)	1.423	.650
	hip_circumference	1.657	.603
	Height (m)	1.167	.775
3	hip_circumference	1.764	.529
	Height (m)	2.316	.354
4	Height (m)	2.473	.354

a. Dependent Variable: GT_Soft Tissue R (cm)

b. Predictors in the Model: (Constant), ant_mid-thigh_SF_R

c. Predictors in the Model: (Constant), ant_mid-thigh_SF_R, max-thigh_M-L_breadth_R

d. Predictors in the Model: (Constant), ant_mid-thigh_SF_R, max-thigh_M-L_breadth_R, Sex Code (0=F, 1=M)

e. Predictors in the Model: (Constant), ant_mid-thigh_SF_R, max-thigh_M-L_breadth_R, Sex Code (0=F,

Coefficient Correlations[a]

Model			ant_mid-thigh_SF_R	max-thigh_M-L_breadth_R	Sex Code (0=F, 1=M)
1	Correlations	ant_mid-thigh_SF_R	1.000		
	Covariances	ant_mid-thigh_SF_R	.000		
2	Correlations	ant_mid-thigh_SF_R	1.000	-.310	
		max-thigh_M-L_breadth_R	-.310	1.000	
	Covariances	ant_mid-thigh_SF_R	.000	.000	
		max-thigh_M-L_breadth_R	.000	.006	
3	Correlations	ant_mid-thigh_SF_R	1.000	-.274	.530
		max-thigh_M-L_breadth_R	-.274	1.000	-.021
		Sex Code (0=F,1=M)	.530	-.021	1.000
	Covariances	ant_mid-thigh_SF_R	.000	.000	.002
		max-thigh_M-L_breadth_R	.000	.004	.000
		Sex Code (0=F,1=M)	.002	.000	.075
4	Correlations	ant_mid-thigh_SF_R	1.000	-.004	.569
		max-thigh_M-L_breadth_R	-.004	1.000	.103
		Sex Code (0=F,1=M)	.569	.103	1.000
		hip_circumference	-.431	-.492	-.245
	Covariances	ant_mid-thigh_SF_R	.000	-2.594E-6	.002
		max-thigh_M-L_breadth_R	-2.594E-6	.004	.002
		Sex Code (0=F,1=M)	.002	.002	.063
		hip_circumference	-7.630E-5	-.001	-.001
5	Correlations	ant_mid-thigh_SF_R	1.000	-.003	.363
		max-thigh_M-L_breadth_R	-.003	1.000	.069
		Sex Code (0=F,1=M)	.363	.069	1.000
		hip_circumference	-.435	-.478	-.002
		Height (m)	.077	.010	-.681
	Covariances	ant_mid-thigh_SF_R	.000	-1.897E-6	.001
		max-thigh_M-L_breadth_R	-1.897E-6	.004	.001

Coefficient Correlations[a]

Model			hip_circumference	Height (m)
1	Correlations	ant_mid-thigh_SF_R		
	Covariances	ant_mid-thigh_SF_R		
2	Correlations	ant_mid-thigh_SF_R		
		max-thigh_M-L_breadth_R		
	Covariances	ant_mid-thigh_SF_R		
		max-thigh_M-L_breadth_R		
3	Correlations	ant_mid-thigh_SF_R		
		max-thigh_M-L_breadth_R		
		Sex Code (0=F,1=M)		
	Covariances	ant_mid-thigh_SF_R		
		max-thigh_M-L_breadth_R		
		Sex Code (0=F,1=M)		
4	Correlations	ant_mid-thigh_SF_R	-.431	
		max-thigh_M-L_breadth_R	-.492	
		Sex Code (0=F,1=M)	-.245	
		hip_circumference	1.000	
	Covariances	ant_mid-thigh_SF_R	-7.630E-5	
		max-thigh_M-L_breadth_R	-.001	
		Sex Code (0=F,1=M)	-.001	
		hip_circumference	.000	
5	Correlations	ant_mid-thigh_SF_R	-.435	.077
		max-thigh_M-L_breadth_R	-.478	.010
		Sex Code (0=F,1=M)	-.002	-.681
		hip_circumference	1.000	-.252
		Height (m)	-.252	1.000
	Covariances	ant_mid-thigh_SF_R	-7.395E-5	.001
		max-thigh_M-L_breadth_R	.000	.001

Coefficient Correlations[a]

Model		ant_mid-thigh_SF_R	max-thigh_M-L_breadth_R	Sex Code (0=F, 1=M)
	Sex Code (0=F,1=M)	.001	.001	.110
	hip_circumference	-7.395E-5	.000	-1.138E-5
	Height (m)	.001	.001	-.331

Coefficient Correlations[a]

Model		hip_circumference	Height (m)
	Sex Code (0=F,1=M)	-1.138E-5	-.331
	hip_circumference	.000	-.006
	Height (m)	-.006	2.157

a. Dependent Variable: GT_Soft Tissue R (cm)

Collinearity Diagnostics[a]

Model	Dimension	Eigenvalue	Condition Index	Variance Proportions (Constant)	Variance Proportions ant_mid-thigh_SF_R	Variance Proportions max-thigh_M-L_breadth_R
1	1	1.888	1.000	.06	.06	
	2	.112	4.104	.94	.94	
2	1	2.854	1.000	.00	.02	.00
	2	.140	4.513	.02	.93	.01
	3	.006	22.601	.98	.05	.99
3	1	3.345	1.000	.00	.01	.00
	2	.575	2.412	.00	.06	.00
	3	.074	6.732	.03	.91	.03
	4	.006	24.601	.97	.02	.97
4	1	4.332	1.000	.00	.00	.00
	2	.579	2.735	.00	.05	.00
	3	.081	7.322	.01	.79	.01
	4	.006	27.541	.34	.00	.87
	5	.003	40.976	.65	.16	.12
5	1	5.323	1.000	.00	.00	.00
	2	.579	3.031	.00	.05	.00
	3	.087	7.800	.00	.76	.01
	4	.007	27.448	.04	.02	.75

Collinearity Diagnostics[a]

Model	Dimension	Variance Proportions: Sex Code (0=F, 1=M)	hip_circumference	Height (m)
1	1			
	2			
2	1			
	2			
	3			
3	1	.02		
	2	.44		
	3	.53		
	4	.01		
4	1	.01	.00	
	2	.42	.00	
	3	.52	.00	
	4	.02	.03	
	5	.03	.97	
5	1	.00	.00	.00
	2	.23	.00	.00
	3	.30	.00	.00
	4	.03	.00	.04

Collinearity Diagnostics[a]

Model	Dimension	Eigenvalue	Condition Index	Variance Proportions: (Constant)	ant_mid-thigh_SF_R	max-thigh_M-L_breadth_R
	5	.003	43.866	.06	.16	.23
	6	.001	77.710	.90	.00	.01

Collinearity Diagnostics[a]

Model	Dimension	Variance Proportions: Sex Code (0=F, 1=M)	hip_circumference	Height (m)
	5	.00	.99	.03
	6	.43	.01	.93

a. Dependent Variable: GT_Soft Tissue R (cm)

Residuals Statistics[a]

	Minimum	Maximum	Mean	Std. Deviation	N
Predicted Value	1.2240	9.2725	4.7012	1.93021	76
Std. Predicted Value	-1.801	2.368	.000	1.000	76
Standard Error of Predicted Value	.143	.410	.234	.060	76
Adjusted Predicted Value	1.2197	9.2488	4.6967	1.92907	76
Residual	-2.25602	2.01755	.00000	.82977	76
Std. Residual	-2.627	2.349	.000	.966	76
Stud. Residual	-2.990	2.655	.002	1.021	76
Deleted Residual	-2.92334	2.57658	.00444	.93015	76
Stud. Deleted Residual	-3.178	2.779	.003	1.044	76
Mahal. Distance	1.101	16.134	4.934	3.210	76
Cook's Distance	.000	.441	.021	.063	76
Centered Leverage Value	.015	.215	.066	.043	76

a. Dependent Variable: GT_Soft Tissue R (cm)

Charts

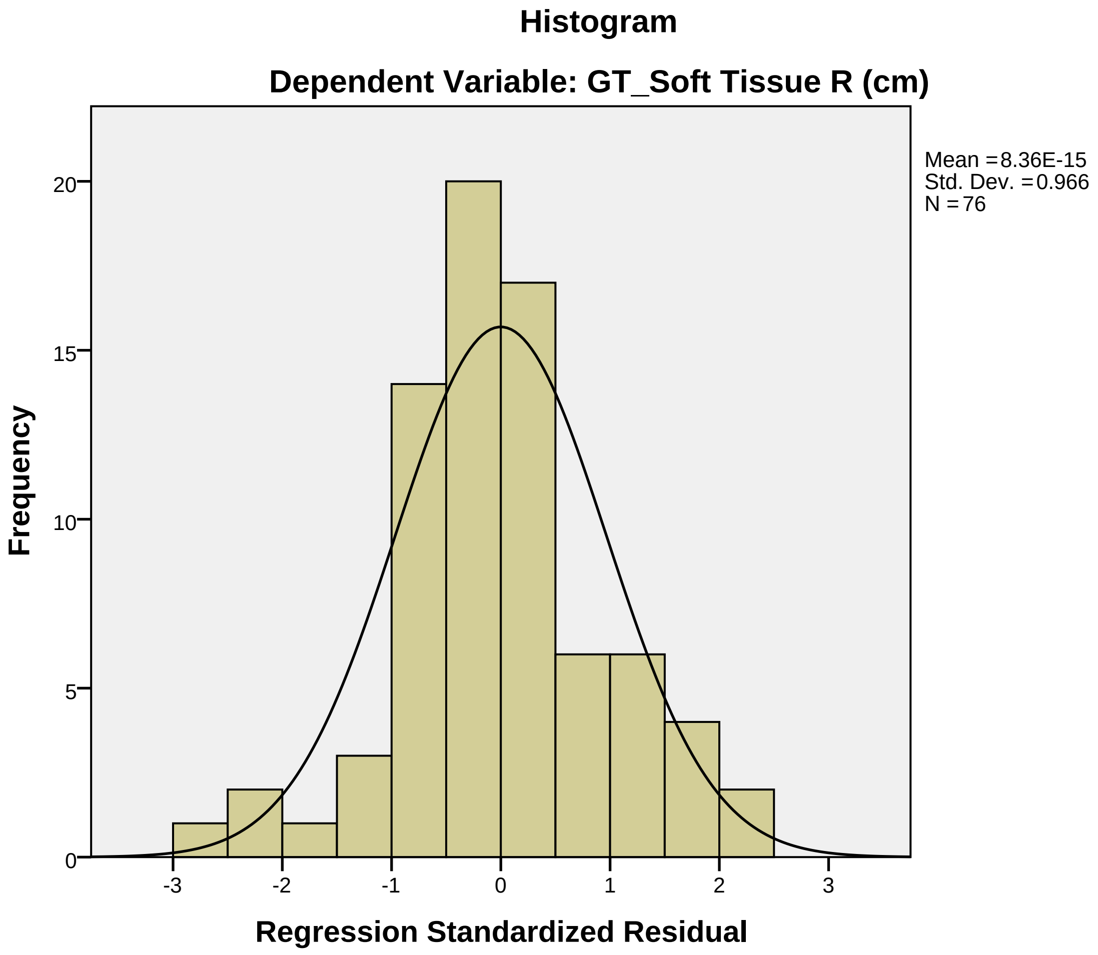
Histogram
Dependent Variable: GT_Soft Tissue R (cm)
Mean =8.36E-15
Std. Dev. =0.966
N =76
Frequency
0
5
10
15
20
-3
-2
-1
0
1
2
3
Regression Standardized Residual

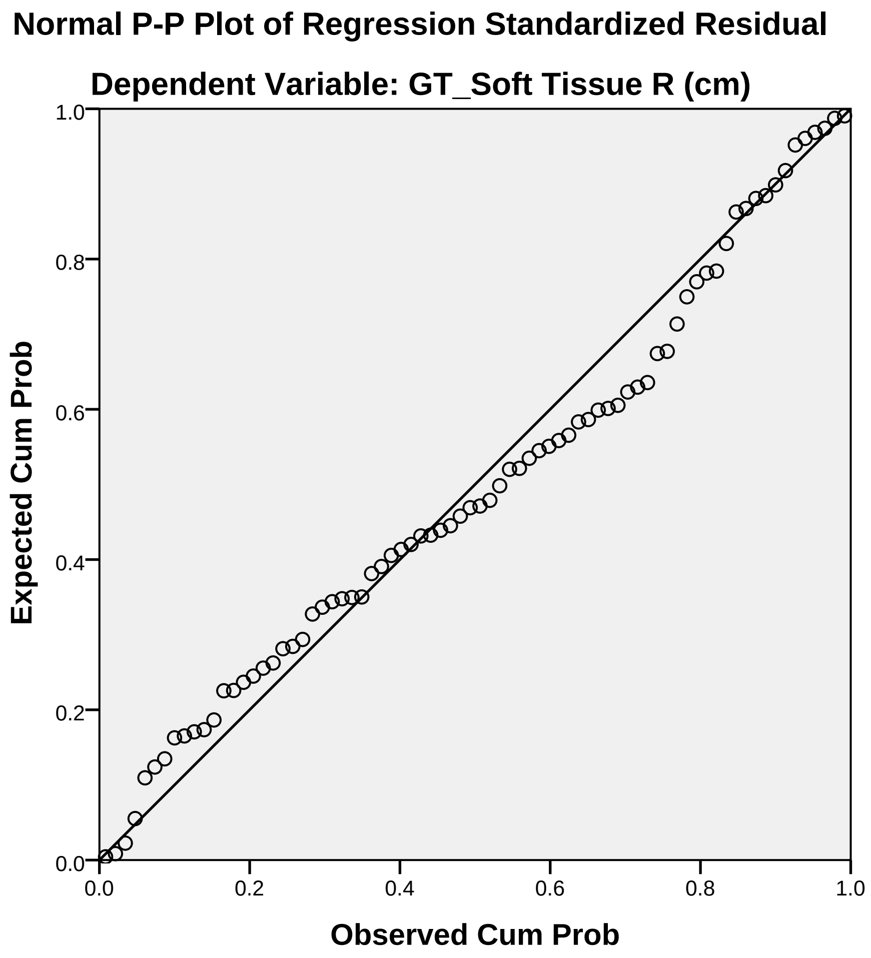

Normal P-P Plot of Regression Standardized Residual
Dependent Variable: GT_Soft Tissue R (cm)
Expected Cum Prob
Observed Cum Prob
0.0
0.2
0.4
0.6
0.8
1.0

Scatterplot

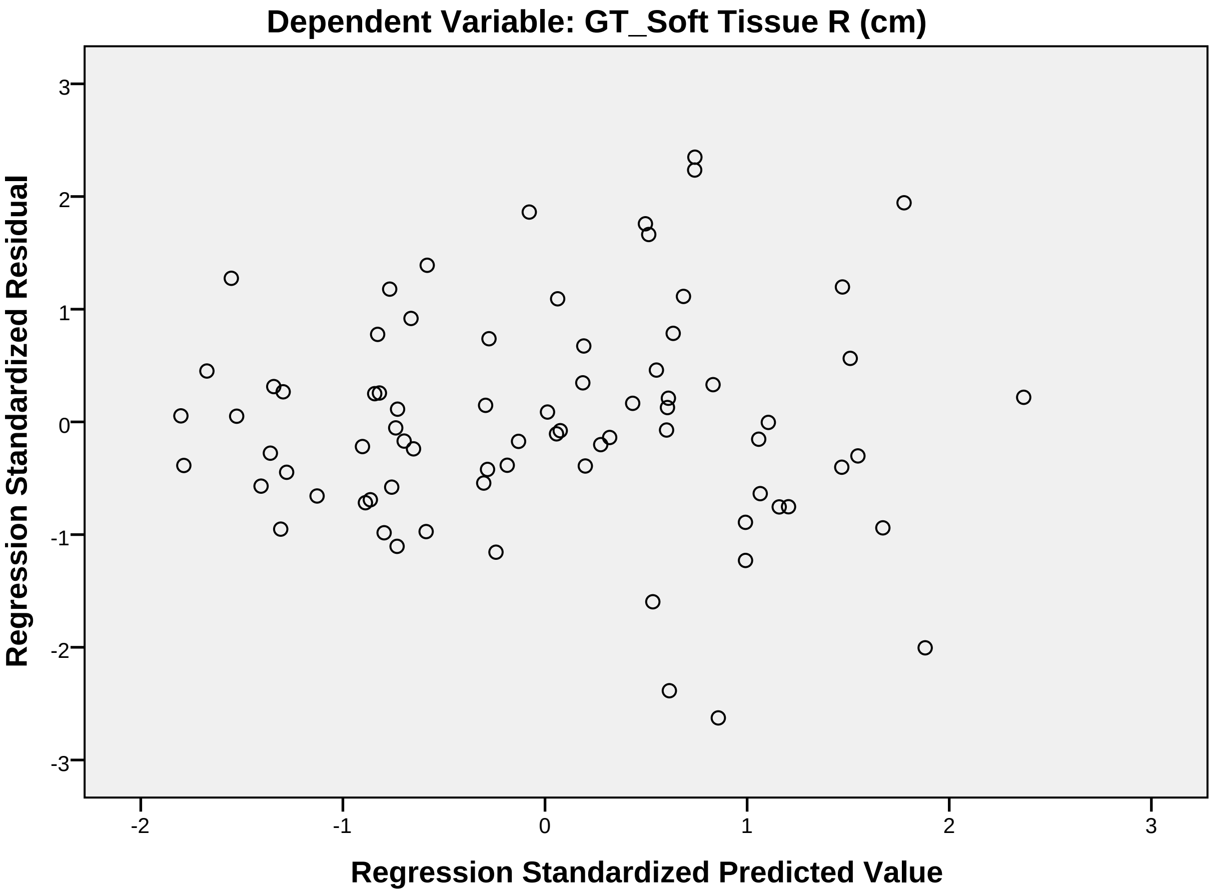

Regression

Notes

Output Created		22-NOV-2017 13:25:15
Comments		
Input	Data	F:\Claudia Regression\Danielle FIX\Generation-Validation Groups\SPSS Data_REGRESSION_G groups final_OLDER_no 0fm488+0mt203.sav
	Active Dataset	DataSet1
	Filter	<none>
	Weight	<none>
	Split File	<none>
	N of Rows in Working Data File	76
Missing Value Handling	Definition of Missing	User-defined missing values are treated as missing.
	Cases Used	Statistics are based on cases with no missing values for any variable used.

Notes

Syntax		REGRESSION<br>/MISSING LISTWISE<br>/STATISTICS COEFF OUTS BCOV R ANOVA COLLIN TOL<br>/CRITERIA=PIN(.05) POUT(.10)<br>/NOORIGIN<br>/DEPENDENT GT_SoftTissueLcm<br>/METHOD=STEPWISE Age SexCode0F1M Heightm BodyMasskg waist_circumference waist_breadth_ML<br>waist_breadth_AP suprailiac_SF abdomen_SF ant_pelvis_length lat_pelvis_length_left AVG_lat_pelvis<br>pelvis_circumference hip_breadth_ML pelvis_breadth_AP leftlat.thigh_length leftmed.thigh_length<br>leftmid.thigh_length midthigh_circumference_L upper_thigh_AP_breadth_L maxthigh_ML_breadth_L<br><br>maxthigh_AP_breadth_L ant_midthigh_SF_L post_midthigh_SF_L hip_circumference<br>/SCATTERPLOT=(*ZRESID ,*ZPRED)<br>/RESIDUALS HISTOGRAM(ZRESID) NORMPROB(ZRESID)<br>/CASEWISE PLOT(ZRESID) OUTLIERS(3)<br>/SAVE ZPRED MAHAL COOK ZRESID.
Resources	Processor Time	00:00:00.45
	Elapsed Time	00:00:00.30

Notes

	Memory Required	19764 bytes
	Additional Memory Required for Residual Plots	720 bytes
Variables Created or Modified	ZPR_11	Standardized Predicted Value
	ZRE_11	Standardized Residual
	MAH_11	Mahalanobis Distance
	COO_11	Cook's Distance

Variables Entered/Removed[a]

Model	Variables Entered	Variables Removed	Method
1	ant_mid-thigh_SF_L	.	Stepwise (Criteria: Probability-of-F-to-enter <= .050, Probability-of-F-to-remove >= .100).
2	Sex Code (0=F,1=M)	.	Stepwise (Criteria: Probability-of-F-to-enter <= .050, Probability-of-F-to-remove >= .100).
3	hip_circumference	.	Stepwise (Criteria: Probability-of-F-to-enter <= .050, Probability-of-F-to-remove >= .100).

Variables Entered/Removed[a]

Model	Variables Entered	Variables Removed	Method
4	max-thigh_M-L_breadth_L	.	Stepwise (Criteria: Probability-of-F-to-enter <= .050, Probability-of-F-to-remove >= .100).
5	Height (m)	.	Stepwise (Criteria: Probability-of-F-to-enter <= .050, Probability-of-F-to-remove >= .100).

a. Dependent Variable: GT_Soft Tissue L (cm)

Model Summary[f]

Model	R	R Square	Adjusted R Square	Std. Error of the Estimate
1	.774[a]	.599	.593	1.28854
2	.834[b]	.696	.688	1.12849
3	.891[c]	.794	.786	.93482
4	.905[d]	.818	.808	.88510
5	.911[e]	.831	.819	.86007

a. Predictors: (Constant), ant_mid-thigh_SF_L

b. Predictors: (Constant), ant_mid-thigh_SF_L, Sex Code (0=F,1=M)

c. Predictors: (Constant), ant_mid-thigh_SF_L, Sex Code (0=F,1=M), hip_circumference

d. Predictors: (Constant), ant_mid-thigh_SF_L, Sex Code (0=F,1=M), hip_circumference, max-thigh_M-L_breadth_L

e. Predictors: (Constant), ant_mid-thigh_SF_L, Sex Code (0=F,1=M), hip_circumference, max-thigh_M-L_breadth_L, Height (m)

f. Dependent Variable: GT_Soft Tissue L (cm)

ANOVA[a]

Model		Sum of Squares	df	Mean Square	F	Sig.
1	Regression	183.157	1	183.157	110.314	.000[b]
	Residual	122.864	74	1.660		
	Total	306.021	75			
2	Regression	213.056	2	106.528	83.650	.000[c]
	Residual	92.965	73	1.273		
	Total	306.021	75			
3	Regression	243.102	3	81.034	92.728	.000[d]
	Residual	62.920	72	.874		
	Total	306.021	75			
4	Regression	250.399	4	62.600	79.907	.000[e]
	Residual	55.622	71	.783		
	Total	306.021	75			
5	Regression	254.241	5	50.848	68.740	.000[f]
	Residual	51.781	70	.740		
	Total	306.021	75			

a. Dependent Variable: GT_Soft Tissue L (cm)

b. Predictors: (Constant), ant_mid-thigh_SF_L

c. Predictors: (Constant), ant_mid-thigh_SF_L, Sex Code (0=F,1=M)

d. Predictors: (Constant), ant_mid-thigh_SF_L, Sex Code (0=F,1=M), hip_circumference

e. Predictors: (Constant), ant_mid-thigh_SF_L, Sex Code (0=F,1=M), hip_circumference, max-thigh_M-L_breadth_L

f. Predictors: (Constant), ant_mid-thigh_SF_L, Sex Code (0=F,1=M), hip_circumference, max-thigh_M-L_breadth_L, Height (m)

Coefficients[a]

Model		Unstandardized Coefficients B	Std. Error	Standardized Coefficients Beta	t	Sig.
1	(Constant)	1.307	.332		3.933	.000
	ant_mid-thigh_SF_L	.123	.012	.774	10.503	.000
2	(Constant)	2.974	.451		6.599	.000
	ant_mid-thigh_SF_L	.088	.013	.551	6.966	.000
	Sex Code (0=F,1=M)	-1.539	.318	-.384	-4.845	.000
3	(Constant)	-4.837	1.383		-3.496	.001
	ant_mid-thigh_SF_L	.051	.012	.322	4.211	.000
	Sex Code (0=F,1=M)	-1.950	.272	-.486	-7.161	.000
	hip_circumference	.087	.015	.366	5.864	.000
4	(Constant)	-6.187	1.383		-4.475	.000
	ant_mid-thigh_SF_L	.049	.012	.306	4.221	.000
	Sex Code (0=F,1=M)	-1.819	.261	-.453	-6.959	.000
	hip_circumference	.065	.016	.272	4.087	.000
	max-thigh_M-L_breadth_L	.212	.070	.191	3.052	.003
5	(Constant)	-1.644	2.404		-.684	.496
	ant_mid-thigh_SF_L	.047	.011	.292	4.129	.000
	Sex Code (0=F,1=M)	-1.310	.338	-.326	-3.872	.000
	hip_circumference	.074	.016	.312	4.649	.000
	max-thigh_M-L_breadth_L	.212	.068	.191	3.141	.002
	Height (m)	-3.354	1.472	-.176	-2.279	.026

Coefficients[a]

Model		Collinearity Statistics	
		Tolerance	VIF
1	(Constant)		
	ant_mid-thigh_SF_L	1.000	1.000
2	(Constant)		
	ant_mid-thigh_SF_L	.664	1.506
	Sex Code (0=F,1=M)	.664	1.506
3	(Constant)		
	ant_mid-thigh_SF_L	.489	2.043
	Sex Code (0=F,1=M)	.620	1.612
	hip_circumference	.732	1.367
4	(Constant)		
	ant_mid-thigh_SF_L	.487	2.053
	Sex Code (0=F,1=M)	.603	1.657
	hip_circumference	.576	1.736
	max-thigh_M-L_breadth_L	.655	1.528
5	(Constant)		
	ant_mid-thigh_SF_L	.483	2.069
	Sex Code (0=F,1=M)	.340	2.939
	hip_circumference	.538	1.857
	max-thigh_M-L_breadth_L	.655	1.528
	Height (m)	.404	2.477

a. Dependent Variable: GT_Soft Tissue L (cm)

Excluded Variables[a]

Model		Beta In	t	Sig.	Partial Correlation	Collinearity . Tolerance
1	Age	-.020[b]	-.264	.793	-.031	.992
	Sex Code (0=F,1=M)	-.384[b]	-4.845	.000	-.493	.664
	Height (m)	-.250[b]	-3.319	.001	-.362	.845
	Body Mass (kg)	-.007[b]	-.093	.926	-.011	1.000
	waist_circumference	.038[b]	.511	.611	.060	.982
	waist_breadth_M-L	-.038[b]	-.514	.608	-.060	.993
	waist_breadth_A-P	.070[b]	.937	.352	.109	.963
	suprailiac_SF	.063[b]	.753	.454	.088	.771
	abdomen_SF	-.003[b]	-.042	.966	-.005	.914
	ant_pelvis_length	-.005[b]	-.072	.943	-.008	.992
	lat_pelvis_length_left	-.175[b]	-2.394	.019	-.270	.951
	AVG_lat_pelvis	-.192[b]	-2.641	.010	-.295	.951
	pelvis_circumference	.181[b]	2.311	.024	.261	.832
	hip_breadth_M-L	.243[b]	3.145	.002	.345	.814
	pelvis_breadth_A-P	.138[b]	1.845	.069	.211	.933
	left lat. thigh_length	-.214[b]	-2.834	.006	-.315	.869
	left med. thigh_length	-.147[b]	-1.954	.055	-.223	.921
	left mid. thigh_length	-.017[b]	-.233	.817	-.027	.978
	mid-thigh_circumference_L	.202[b]	2.616	.011	.293	.844
	upper_thigh_A-P_breadth_L	.165[b]	2.070	.042	.235	.813
	max-thigh_M-L_breadth_L	.319[b]	4.414	.000	.459	.832
	max-thigh_A-P_breadth_L	.176[b]	2.354	.021	.266	.912
	post_mid-thigh_SF_L	.054[b]	.360	.720	.042	.240
	hip_circumference	.251[b]	3.201	.002	.351	.784
2	Age	-.011[c]	-.164	.870	-.019	.992
	Height (m)	-.027[c]	-.276	.783	-.033	.440
	Body Mass (kg)	.332[c]	4.408	.000	.461	.585
	waist_circumference	.224[c]	3.269	.002	.360	.786
	waist_breadth_M-L	.195[c]	2.612	.011	.294	.688

Excluded Variables[a]

Model		Collinearity Statistics VIF	Minimum Tolerance
1	Age	1.008	.992
	Sex Code (0=F,1=M)	1.506	.664
	Height (m)	1.183	.845
	Body Mass (kg)	1.000	1.000
	waist_circumference	1.019	.982
	waist_breadth_M-L	1.007	.993
	waist_breadth_A-P	1.038	.963
	suprailiac_SF	1.298	.771
	abdomen_SF	1.094	.914
	ant_pelvis_length	1.009	.992
	lat_pelvis_length_left	1.051	.951
	AVG_lat_pelvis	1.052	.951
	pelvis_circumference	1.202	.832
	hip_breadth_M-L	1.228	.814
	pelvis_breadth_A-P	1.071	.933
	left lat. thigh_length	1.151	.869
	left med. thigh_length	1.086	.921
	left mid. thigh_length	1.023	.978
	mid-thigh_circumference_L	1.185	.844
	upper_thigh_A-P_breadth_L	1.230	.813
	max-thigh_M-L_breadth_L	1.202	.832
	max-thigh_A-P_breadth_L	1.096	.912
	post_mid-thigh_SF_L	4.166	.240
	hip_circumference	1.276	.784
2	Age	1.008	.659
	Height (m)	2.275	.345
	Body Mass (kg)	1.709	.389
	waist_circumference	1.272	.532
	waist_breadth_M-L	1.453	.460

Excluded Variables[a]

Model		Beta In	t	Sig.	Partial Correlation	Collinearity . Tolerance
	waist_breadth_A-P	.183[c]	2.776	.007	.311	.875
	suprailiac_SF	.183[c]	2.469	.016	.279	.704
	abdomen_SF	.154[c]	2.137	.036	.244	.765
	ant_pelvis_length	-.023[c]	-.354	.724	-.042	.988
	lat_pelvis_length_left	-.074[c]	-1.050	.297	-.123	.839
	AVG_lat_pelvis	-.086[c]	-1.211	.230	-.141	.823
	pelvis_circumference	.296[c]	4.540	.000	.472	.771
	hip_breadth_M-L	.334[c]	5.348	.000	.533	.776
	pelvis_breadth_A-P	.213[c]	3.325	.001	.365	.895
	left lat. thigh_length	-.095[c]	-1.269	.208	-.148	.731
	left med. thigh_length	.028[c]	.352	.726	.041	.677
	left mid. thigh_length	.020[c]	.301	.764	.035	.964
	mid-thigh_circumference_L	.332[c]	5.291	.000	.529	.769
	upper_thigh_A-P_breadth_L	.328[c]	4.926	.000	.502	.713
	max-thigh_M-L_breadth_L	.309[c]	5.042	.000	.511	.832
	max-thigh_A-P_breadth_L	.323[c]	5.258	.000	.527	.809
	post_mid-thigh_SF_L	-.031[c]	-.229	.820	-.027	.236
	hip_circumference	.366[c]	5.864	.000	.568	.732
3	Age	-.048[d]	-.889	.377	-.105	.978
	Height (m)	-.176[d]	-2.149	.035	-.247	.404
	Body Mass (kg)	-.101[d]	-.710	.480	-.084	.142
	waist_circumference	-.119[d]	-1.269	.208	-.149	.320
	waist_breadth_M-L	-.163[d]	-1.771	.081	-.206	.328
	waist_breadth_A-P	-.130[d]	-1.575	.120	-.184	.410
	suprailiac_SF	-.095[d]	-1.152	.253	-.135	.414
	abdomen_SF	-.049[d]	-.680	.499	-.080	.551
	ant_pelvis_length	-.093[d]	-1.709	.092	-.199	.946
	lat_pelvis_length_left	-.033[d]	-.565	.574	-.067	.827
	AVG_lat_pelvis	-.034[d]	-.571	.570	-.068	.804

Excluded Variables[a]

Model		Collinearity Statistics VIF	Minimum Tolerance
	waist_breadth_A-P	1.142	.591
	suprailiac_SF	1.419	.470
	abdomen_SF	1.308	.519
	ant_pelvis_length	1.012	.662
	lat_pelvis_length_left	1.192	.586
	AVG_lat_pelvis	1.215	.575
	pelvis_circumference	1.298	.513
	hip_breadth_M-L	1.289	.520
	pelvis_breadth_A-P	1.117	.595
	left lat. thigh_length	1.369	.558
	left med. thigh_length	1.477	.488
	left mid. thigh_length	1.037	.640
	mid-thigh_circumference_L	1.300	.511
	upper_thigh_A-P_breadth_L	1.403	.473
	max-thigh_M-L_breadth_L	1.203	.592
	max-thigh_A-P_breadth_L	1.236	.542
	post_mid-thigh_SF_L	4.240	.227
	hip_circumference	1.367	.489
3	Age	1.022	.489
	Height (m)	2.477	.345
	Body Mass (kg)	7.063	.142
	waist_circumference	3.125	.298
	waist_breadth_M-L	3.046	.328
	waist_breadth_A-P	2.438	.343
	suprailiac_SF	2.413	.414
	abdomen_SF	1.814	.468
	ant_pelvis_length	1.057	.487
	lat_pelvis_length_left	1.209	.487
	AVG_lat_pelvis	1.244	.484

Excluded Variables[a]

Model		Beta In	t	Sig.	Partial Correlation	Collinearity . Tolerance
	pelvis_circumference	-.019[d]	-.168	.867	-.020	.220
	hip_breadth_M-L	.109[d]	.932	.354	.110	.208
	pelvis_breadth_A-P	-.025[d]	-.317	.752	-.038	.479
	left lat. thigh_length	-.116[d]	-1.883	.064	-.218	.728
	left med. thigh_length	-.062[d]	-.929	.356	-.110	.642
	left mid. thigh_length	-.035[d]	-.638	.526	-.075	.936
	mid-thigh_circumference_L	.197[d]	2.861	.006	.322	.546
	upper_thigh_A-P_breadth_L	.168[d]	2.203	.031	.253	.467
	max-thigh_M-L_breadth_L	.191[d]	3.052	.003	.341	.655
	max-thigh_A-P_breadth_L	.197[d]	3.030	.003	.338	.604
	post_mid-thigh_SF_L	.013[d]	.114	.909	.014	.235
4	Age	-.014[e]	-.262	.794	-.031	.929
	Height (m)	-.176[e]	-2.279	.026	-.263	.404
	Body Mass (kg)	-.165[e]	-1.221	.226	-.144	.138
	waist_circumference	-.055[e]	-.589	.558	-.070	.300
	waist_breadth_M-L	-.127[e]	-1.437	.155	-.169	.322
	waist_breadth_A-P	-.050[e]	-.586	.560	-.070	.357
	suprailiac_SF	-.080[e]	-1.022	.310	-.121	.413
	abdomen_SF	-.034[e]	-.491	.625	-.059	.548
	ant_pelvis_length	-.069[e]	-1.317	.192	-.155	.922
	lat_pelvis_length_left	-.022[e]	-.389	.699	-.046	.823
	AVG_lat_pelvis	-.022[e]	-.386	.700	-.046	.800
	pelvis_circumference	.012[e]	.110	.913	.013	.218
	hip_breadth_M-L	.087[e]	.783	.436	.093	.207
	pelvis_breadth_A-P	-.021[e]	-.285	.776	-.034	.479
	left lat. thigh_length	-.096[e]	-1.630	.108	-.191	.718
	left med. thigh_length	-.083[e]	-1.318	.192	-.156	.635
	left mid. thigh_length	-.038[e]	-.724	.472	-.086	.936

Excluded Variables[a]

Model		Collinearity Statistics VIF	Minimum Tolerance
	pelvis_circumference	4.543	.209
	hip_breadth_M-L	4.806	.196
	pelvis_breadth_A-P	2.087	.392
	left lat. thigh_length	1.373	.484
	left med. thigh_length	1.557	.482
	left mid. thigh_length	1.068	.486
	mid-thigh_circumference_L	1.831	.465
	upper_thigh_A-P_breadth_L	2.140	.449
	max-thigh_M-L_breadth_L	1.528	.487
	max-thigh_A-P_breadth_L	1.656	.475
	post_mid-thigh_SF_L	4.260	.195
4	Age	1.076	.487
	Height (m)	2.477	.340
	Body Mass (kg)	7.221	.138
	waist_circumference	3.332	.230
	waist_breadth_M-L	3.108	.287
	waist_breadth_A-P	2.801	.238
	suprailiac_SF	2.423	.361
	abdomen_SF	1.825	.428
	ant_pelvis_length	1.085	.485
	lat_pelvis_length_left	1.215	.484
	AVG_lat_pelvis	1.250	.481
	pelvis_circumference	4.584	.185
	hip_breadth_M-L	4.827	.188
	pelvis_breadth_A-P	2.087	.340
	left lat. thigh_length	1.392	.482
	left med. thigh_length	1.575	.464
	left mid. thigh_length	1.068	.484

Excluded Variables[a]

Model		Beta In	t	Sig.	Partial Correlation	Collinearity . Tolerance
	mid-thigh_circumference_L	.109[e]	1.254	.214	.148	.333
	upper_thigh_A-P_breadth_L	.083[e]	1.002	.320	.119	.373
	max-thigh_A-P_breadth_L	.136[e]	1.906	.061	.222	.485
	post_mid-thigh_SF_L	-.055[e]	-.516	.608	-.062	.225
5	Age	-.020[f]	-.384	.702	-.046	.927
	Body Mass (kg)	-.062[f]	-.433	.667	-.052	.119
	waist_circumference	-.099[f]	-1.084	.282	-.129	.288
	waist_breadth_M-L	-.129[f]	-1.507	.136	-.179	.322
	waist_breadth_A-P	-.080[f]	-.963	.339	-.115	.349
	suprailiac_SF	-.096[f]	-1.251	.215	-.149	.410
	abdomen_SF	-.021[f]	-.313	.755	-.038	.544
	ant_pelvis_length	-.058[f]	-1.128	.263	-.135	.912
	lat_pelvis_length_left	.002[f]	.037	.971	.004	.793
	AVG_lat_pelvis	-.001[f]	-.025	.980	-.003	.778
	pelvis_circumference	-.010[f]	-.097	.923	-.012	.216
	hip_breadth_M-L	.059[f]	.538	.592	.065	.204
	pelvis_breadth_A-P	-.043[f]	-.601	.550	-.072	.471
	left lat. thigh_length	-.034[f]	-.480	.632	-.058	.501
	left med. thigh_length	-.034[f]	-.511	.611	-.061	.541
	left mid. thigh_length	-.032[f]	-.622	.536	-.075	.933
	mid-thigh_circumference_L	.098[f]	1.154	.252	.138	.332
	upper_thigh_A-P_breadth_L	.087[f]	1.087	.281	.130	.373
	max-thigh_A-P_breadth_L	.117[f]	1.658	.102	.196	.476
	post_mid-thigh_SF_L	-.064[f]	-.618	.538	-.074	.224

Excluded Variables[a]

Model		Collinearity Statistics VIF	Minimum Tolerance
	mid-thigh_circumference_L	3.002	.333
	upper_thigh_A-P_breadth_L	2.683	.373
	max-thigh_A-P_breadth_L	2.063	.475
	post_mid-thigh_SF_L	4.448	.193
5	Age	1.079	.340
	Body Mass (kg)	8.381	.119
	waist_circumference	3.469	.208
	waist_breadth_M-L	3.108	.276
	waist_breadth_A-P	2.867	.220
	suprailiac_SF	2.440	.330
	abdomen_SF	1.838	.328
	ant_pelvis_length	1.096	.331
	lat_pelvis_length_left	1.262	.332
	AVG_lat_pelvis	1.285	.327
	pelvis_circumference	4.624	.176
	hip_breadth_M-L	4.896	.177
	pelvis_breadth_A-P	2.125	.313
	left lat. thigh_length	1.997	.281
	left med. thigh_length	1.847	.330
	left mid. thigh_length	1.072	.340
	mid-thigh_circumference_L	3.013	.308
	upper_thigh_A-P_breadth_L	2.684	.318
	max-thigh_A-P_breadth_L	2.100	.302
	post_mid-thigh_SF_L	4.455	.193

a. Dependent Variable: GT_Soft Tissue L (cm)

b. Predictors in the Model: (Constant), ant_mid-thigh_SF_L

c. Predictors in the Model: (Constant), ant_mid-thigh_SF_L, Sex Code (0=F,1=M)

d. Predictors in the Model: (Constant), ant_mid-thigh_SF_L, Sex Code (0=F,1=M), hip_circumference

e. Predictors in the Model: (Constant), ant_mid-thigh_SF_L, Sex Code (0=F,1=M), hip_circumference, max-thigh_M-L_breadth_L

f. Predictors in the Model: (Constant), ant_mid-thigh_SF_L, Sex Code (0=F,1=M), hip_circumference, max-thigh_M-L_breadth_L, Height (m)

Coefficient Correlations[a]

Model			ant_mid-thigh_SF_L	Sex Code (0=F, 1=M)	hip_circumference
1	Correlations	ant_mid-thigh_SF_L	1.000		
	Covariances	ant_mid-thigh_SF_L	.000		
2	Correlations	ant_mid-thigh_SF_L	1.000	.580	
		Sex Code (0=F,1=M)	.580	1.000	
	Covariances	ant_mid-thigh_SF_L	.000	.002	
		Sex Code (0=F,1=M)	.002	.101	
3	Correlations	ant_mid-thigh_SF_L	1.000	.613	-.513
		Sex Code (0=F,1=M)	.613	1.000	-.257
		hip_circumference	-.513	-.257	1.000
	Covariances	ant_mid-thigh_SF_L	.000	.002	-9.264E-5
		Sex Code (0=F,1=M)	.002	.074	-.001
		hip_circumference	-9.264E-5	-.001	.000
4	Correlations	ant_mid-thigh_SF_L	1.000	.591	-.421
		Sex Code (0=F,1=M)	.591	1.000	-.301
		hip_circumference	-.421	-.301	1.000
		max-thigh_M-L_breadth_L	-.070	.164	-.461
	Covariances	ant_mid-thigh_SF_L	.000	.002	-7.711E-5
		Sex Code (0=F,1=M)	.002	.068	-.001
		hip_circumference	-7.711E-5	-.001	.000
		max-thigh_M-L_breadth_L	-5.659E-5	.003	-.001
5	Correlations	ant_mid-thigh_SF_L	1.000	.385	-.428
		Sex Code (0=F,1=M)	.385	1.000	-.050
		hip_circumference	-.428	-.050	1.000
		max-thigh_M-L_breadth_L	-.070	.123	-.446
		Height (m)	.087	-.660	-.256
	Covariances	ant_mid-thigh_SF_L	.000	.001	-7.680E-5
		Sex Code (0=F,1=M)	.001	.114	.000
		hip_circumference	-7.680E-5	.000	.000

Coefficient Correlations[a]

Model			max-thigh_M-L_breadth_L	Height (m)
1	Correlations	ant_mid-thigh_SF_L		
	Covariances	ant_mid-thigh_SF_L		
2	Correlations	ant_mid-thigh_SF_L		
		Sex Code (0=F,1=M)		
	Covariances	ant_mid-thigh_SF_L		
		Sex Code (0=F,1=M)		
3	Correlations	ant_mid-thigh_SF_L		
		Sex Code (0=F,1=M)		
		hip_circumference		
	Covariances	ant_mid-thigh_SF_L		
		Sex Code (0=F,1=M)		
		hip_circumference		
4	Correlations	ant_mid-thigh_SF_L	-.070	
		Sex Code (0=F,1=M)	.164	
		hip_circumference	-.461	
		max-thigh_M-L_breadth_L	1.000	
	Covariances	ant_mid-thigh_SF_L	-5.659E-5	
		Sex Code (0=F,1=M)	.003	
		hip_circumference	-.001	
		max-thigh_M-L_breadth_L	.005	
5	Correlations	ant_mid-thigh_SF_L	-.070	.087
		Sex Code (0=F,1=M)	.123	-.660
		hip_circumference	-.446	-.256
		max-thigh_M-L_breadth_L	1.000	.000
		Height (m)	.000	1.000
	Covariances	ant_mid-thigh_SF_L	-5.342E-5	.001
		Sex Code (0=F,1=M)	.003	-.329
		hip_circumference	.000	-.006

Coefficient Correlations[a]

Model		ant_mid-thigh_SF_L	Sex Code (0=F, 1=M)	hip_circumference
	max-thigh_M-L_breadth_L	-5.342E-5	.003	.000
	Height (m)	.001	-.329	-.006

Coefficient Correlations[a]

Model		max-thigh_M-L_breadth_L	Height (m)
	max-thigh_M-L_breadth_L	.005	1.598E-5
	Height (m)	1.598E-5	2.167

a. Dependent Variable: GT_Soft Tissue L (cm)

Collinearity Diagnostics[a]

Model	Dimension	Eigenvalue	Condition Index	Variance Proportions		
				(Constant)	ant_mid-thigh_SF_L	Sex Code (0=F, 1=M)
1	1	1.896	1.000	.05	.05	
	2	.104	4.261	.95	.95	
2	1	2.385	1.000	.01	.02	.04
	2	.566	2.052	.00	.08	.38
	3	.049	7.001	.98	.90	.58
3	1	3.361	1.000	.00	.01	.02
	2	.575	2.417	.00	.05	.39
	3	.061	7.426	.03	.77	.57
	4	.003	35.040	.97	.18	.03
4	1	4.341	1.000	.00	.00	.01
	2	.582	2.731	.00	.04	.39
	3	.070	7.875	.01	.79	.52
	4	.005	29.535	.35	.01	.04
	5	.003	40.621	.64	.16	.05
5	1	5.332	1.000	.00	.00	.00
	2	.582	3.027	.00	.04	.22

Collinearity Diagnostics[a]

Model	Dimension	Variance Proportions hip_circumference	max-thigh_M-L_breadth_L	Height (m)
1	1			
	2			
2	1			
	2			
	3			
3	1	.00		
	2	.00		
	3	.01		
	4	.99		
4	1	.00	.00	
	2	.00	.00	
	3	.00	.01	
	4	.04	.91	
	5	.96	.08	
5	1	.00	.00	.00
	2	.00	.00	.00

Collinearity Diagnostics[a]

Model	Dimension	Eigenvalue	Condition Index	Variance Proportions (Constant)	ant_mid-thigh_SF_L	Sex Code (0=F, 1=M)
	3	.076	8.367	.00	.76	.32
	4	.006	29.486	.04	.04	.05
	5	.003	43.475	.06	.15	.01
	6	.001	77.892	.90	.00	.40

Collinearity Diagnostics[a]

Model	Dimension	Variance Proportions hip_circumference	max-thigh_M-L_breadth_L	Height (m)
	3	.00	.01	.00
	4	.00	.78	.04
	5	.99	.20	.03
	6	.01	.01	.93

a. Dependent Variable: GT_Soft Tissue L (cm)

Residuals Statistics[a]

	Minimum	Maximum	Mean	Std. Deviation	N
Predicted Value	1.2097	8.7628	4.4318	1.84116	76
Std. Predicted Value	-1.750	2.352	.000	1.000	76
Standard Error of Predicted Value	.160	.441	.234	.061	76
Adjusted Predicted Value	1.1952	8.6340	4.4331	1.83925	76
Residual	-1.98921	2.28192	.00000	.83091	76
Std. Residual	-2.313	2.653	.000	.966	76
Stud. Residual	-2.584	2.991	-.001	1.028	76
Deleted Residual	-2.48287	2.90845	-.00129	.94394	76
Stud. Deleted Residual	-2.697	3.180	.003	1.052	76
Mahal. Distance	1.593	18.687	4.934	3.384	76
Cook's Distance	.000	.415	.024	.066	76
Centered Leverage Value	.021	.249	.066	.045	76

a. Dependent Variable: GT_Soft Tissue L (cm)

Charts

Histogram

Dependent Variable: GT_Soft Tissue L (cm)

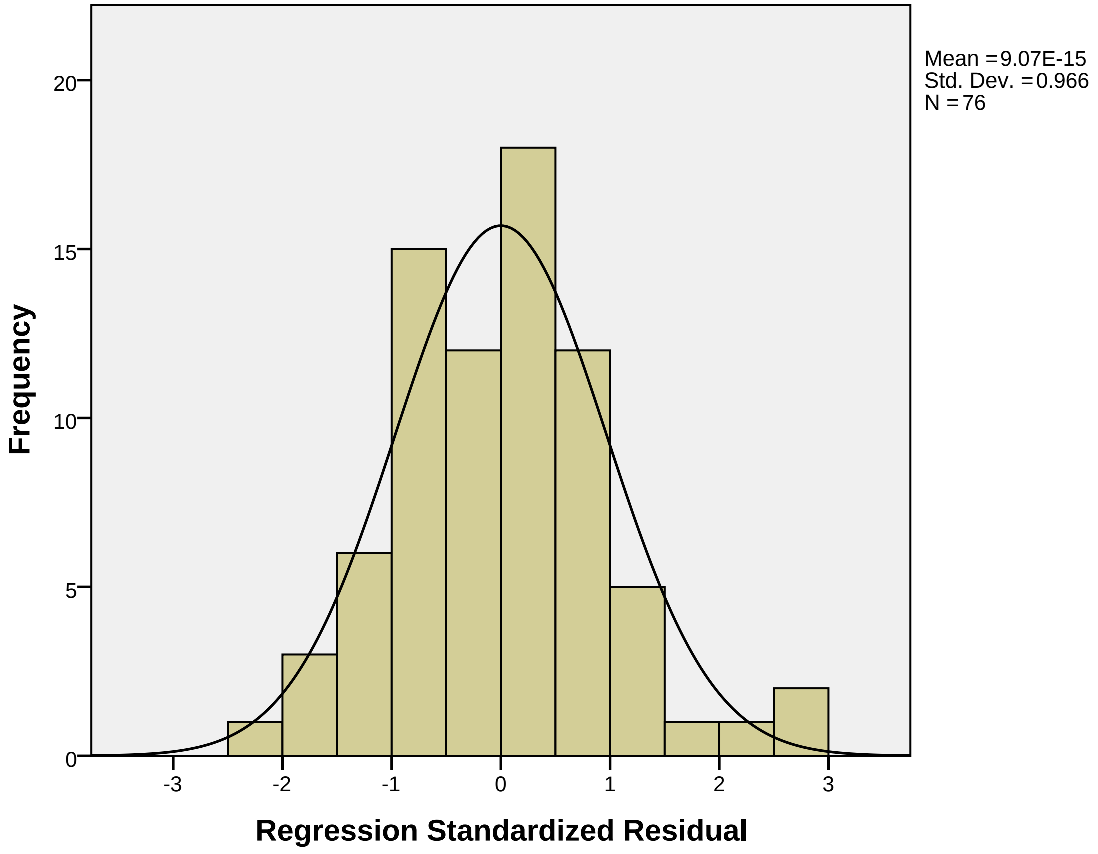

Normal P-P Plot of Regression Standardized Residual

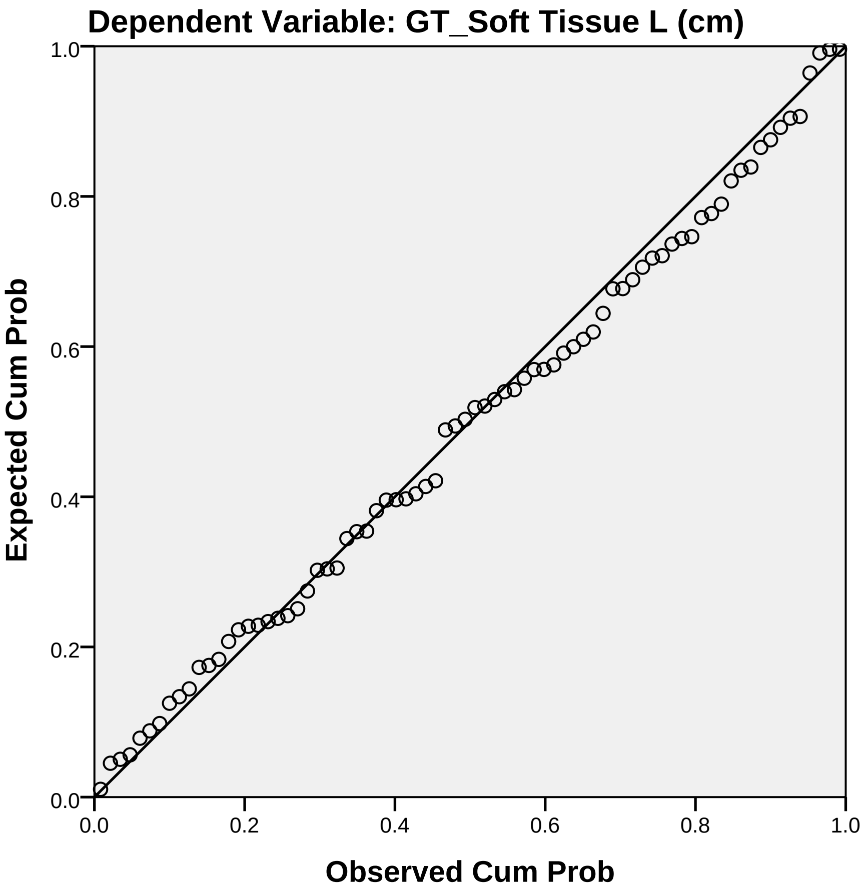

Scatterplot

Dependent Variable: GT_Soft Tissue L (cm)

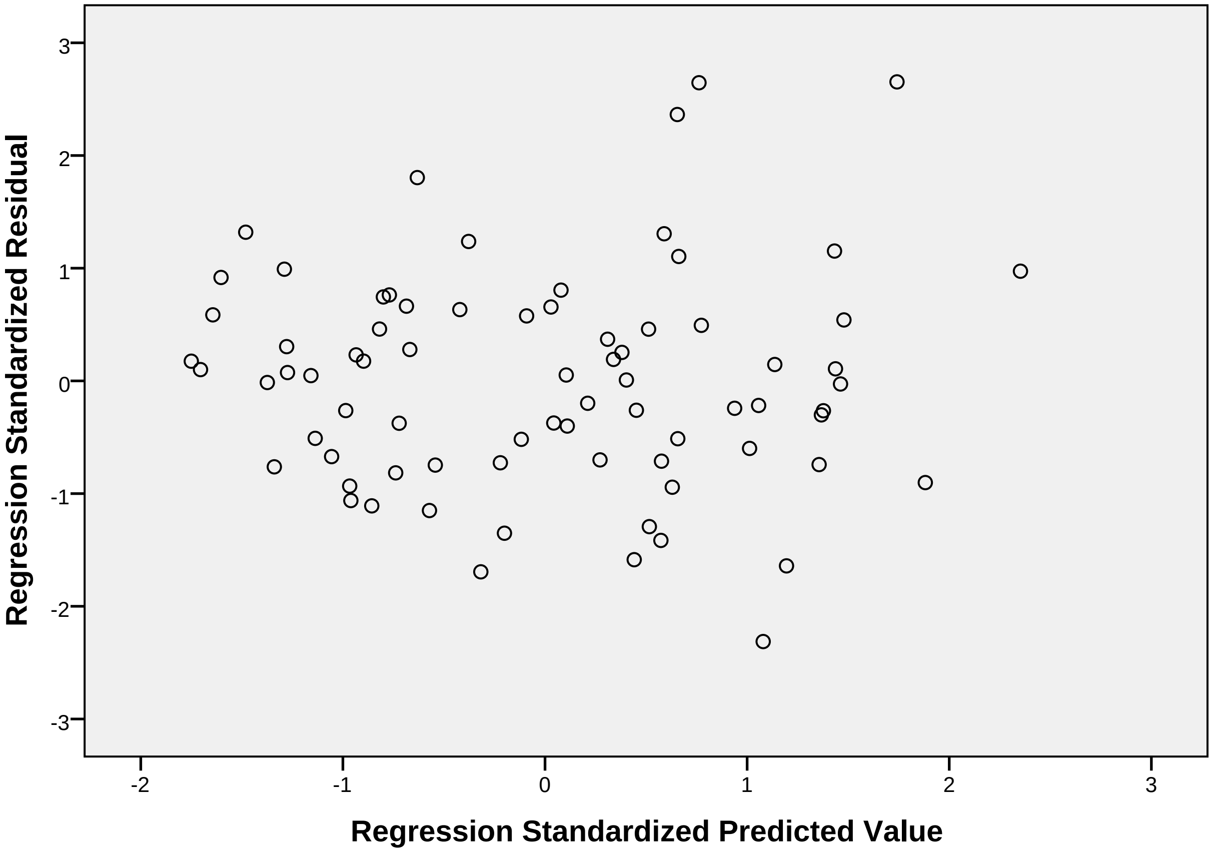

Supplement: S2 File — (PDF) [file pone.0283012.s002.pdf]
