# Supplementary material for: Predicting soft tissue thicknesses overlying the iliac crests and greater trochanters of younger and older adults
Source: PLoS One. 2023 Mar 14;18(3):e0283012. doi: 10.1371/journal.pone.0283012 (PMC10013917; doi:10.1371/journal.pone.0283012)
Supplement: Attachment — Submitted filename: Response to Reviewers.docx [file pone.0283012.s003.docx]

| **Reviewers’ and Editors’ Comments** | **Authors’ Response** | **Page number/section in manuscript** |
| --- | --- | --- |
| Editor |  |  |
| *Please ensure that your manuscript meets PLOS ONE's style requirements, including those for file naming.* | Thank you for your comment. The necessary changes have been made. | See revised manuscript and updated files for proper file naming. |
| *Please provide additional details regarding participant consent. In the ethics statement in the Methods and online submission information, please ensure that you have specified what type you obtained (for instance, written or verbal, and if verbal, how it was documented and witnessed). If your study included minors, state whether you obtained consent from parents or guardians. If the need for consent was waived by the ethics committee, please include this information.* | Thank you for your comment. The type of consent obtained was written consent and has been specified. | See methods section (p. 4) |
| *Please include captions for your Supporting Information files at the end of your manuscript, and update any in-text citations to match accordingly.* | Thank you for your comment. This has been included. | See supporting information section (p. 19) |
| *Please review your reference list to ensure that it is complete and correct. If you have cited papers that have been retracted, please include the rationale for doing so in the manuscript text, or remove these references and replace them with relevant current references. Any changes to the reference list should be mentioned in the rebuttal letter that accompanies your revised manuscript. If you need to cite a retracted article, indicate the article’s retracted status in the References list and also include a citation and full reference for the retraction notice.* | Thank you for your comment. The reference list has been reviewed and updated due to shortening the introduction. | See revised manuscript and references section (p. 18-19) |
| *The introduction section is lengthy. It needs to be shortened* | Thank you for your comment. The introduction section has been shortened. | See introduction section (pg. 2-3) |
| Reviewer #1 |  |  |
| *I only have one comment: in Tables 1 and 2, the mean values of the skin fold should be presented in mm, not in cm as mentioned in the two tables.* | Thank you for your comment. We have revised the tables to present skin fold measurements in mm. | See methods section (pg. 5-6) |
